# Simultaneous detection of novel genes and SNPs by adaptive *p*-value combination

**DOI:** 10.3389/fgene.2022.1009428

**Published:** 2022-11-17

**Authors:** Xiaohui Chen, Hong Zhang, Ming Liu, Hong-Wen Deng, Zheyang Wu

**Affiliations:** ^1^ Department of Mathematical Sciences, Worcester Polytechnic Institute, Worcester, MA, United States; ^2^ Translational Biomarker Statistics, Global Biometrics and Data Management, Pfizer Inc., Cambridge, MA, United States; ^3^ Bioinformatics and Computational Biology Program, Worcester Polytechnic Institute, Worcester, MA, United States; ^4^ Division of Biomedical Informatics & Genomics, School of Medicine, Tulane University, New Orleans, LA, United States

**Keywords:** GWAS summary statistics, SNP-set analysis, *p*-value combination, Fisher’s method, global hypothesis test, osteoporosis, bone density, genetic association

## Abstract

Combining SNP *p*-values from GWAS summary data is a promising strategy for detecting novel genetic factors. Existing statistical methods for the *p*-value-based SNP-set testing confront two challenges. First, the statistical power of different methods depends on unknown patterns of genetic effects that could drastically vary over different SNP sets. Second, they do not identify which SNPs primarily contribute to the global association of the whole set. We propose a new signal-adaptive analysis pipeline to address these challenges using the omnibus thresholding Fisher’s method (oTFisher). The oTFisher remains robustly powerful over various patterns of genetic effects. Its adaptive thresholding can be applied to estimate important SNPs contributing to the overall significance of the given SNP set. We develop efficient calculation algorithms to control the type I error rate, which accounts for the linkage disequilibrium among SNPs. Extensive simulations show that the oTFisher has robustly high power and provides a higher balanced accuracy in screening SNPs than the traditional Bonferroni and FDR procedures. We applied the oTFisher to study the genetic association of genes and haplotype blocks of the bone density-related traits using the summary data of the Genetic Factors for Osteoporosis Consortium. The oTFisher identified more novel and literature-reported genetic factors than existing *p*-value combination methods. Relevant computation has been implemented into the R package TFisher to support similar data analysis.

## 1 Introduction

GWAS summary data is an important resource for dissecting the genetics of complex traits. In contrast to the individual-level genotype and phenotype data, summary data allows much broader access because of less privacy risk (NIH, 2018). The summary statistics are often sufficient for typical genetic association studies with the same efficiency as individual-level data (Lin and Zeng, 2010a,b). Furthermore, it is convenient to integrate summary data from different studies, e.g., through meta-analysis, to cumulate information to increase the power of detecting genetic factors. Many summary data analyses have been carried out and resulted in new genetic findings (Evangelou and Ioannidis, 2013; Pasaniuc and Price, 2017; Guo and Wu, 2019).

GWAS summary data is often used to test the association between a trait and sets of SNPs in genes or other genomic segments. Such SNP-set test can reveal weak genetic effects that are unidentifiable by individual SNPs (Hoh et al., 2001; Xiong et al., 2002; Wu et al., 2010; Wu et al., 2014; Sun et al., 2019; Sun and Lin, 2019). Many methods have been developed based on the combination of SNP statistics (e.g., z-scores) or their *p*-values. Combining the *p*-values has multiple advantages. The *p*-values are the direct measure of statistical significance. Combining them does not concern the problem of signal cancellation in adding SNP z-scores of opposite directions (Pan, 2009). Furthermore, *p*-values are homogeneously Uniform (0, 1) distributed under the null as long as the statistics are continuous. Therefore, *p*-values from statistics of different types or scales can be directly combined.

The SNP-set test is essentially a global hypothesis testing procedure for detecting the existence of “signals” of genetic effects. Optimal signal-detection tests depend on the signal patterns (Donoho and Jin, 2004; Zhang et al., 2020a; Zhang et al., 2020b; Zhang and Wu, 2022a). For example, Fisher’s method (Fisher, 1925) is optimal for detecting dense signals (e.g., in the sense of Bahadur efficiency (Littell and Folks, 1971, 1973)). Meanwhile, the minimal *p*-value test is preferred for detecting sparse and strong signals (Donoho and Jin, 2004). In GWAS, signal patterns depend on the fraction of causal SNPs, the strength of their effects, the linkage disequilibrium (LD) among SNPs, and other potential factors (e.g., covariates) (Zhang and Wu, 2022a). The collective signal patterns are often unknown and drastically vary over different SNP sets. One strategy to address this issue is the omnibus testing procedure. An excellent approach is the ACAT-O, which includes three different tests, the ACAT, the SKAT, and the burden test (Liu et al., 2019). The ACAT is more powerful than SKAT and burden tests for sparse signals when the fraction of causal SNPs is small and the LDs are weak. On the contrary, SKAT and burden tests are more powerful for dense signals. The ACAT-O becomes robust by adapting to the power of these three tests. However, SKAT and burden tests are not *p*-value combination methods. The SKAT requires the marginal score statistics, which may not be provided in summary data (Wu et al., 2011).

We propose an adaptive *p*-value combination procedure based on the thresholding Fisher’s method (TFisher) (Zhang et al., 2020b). The TFisher provides a flexible mechanism for truncating and weighting SNP *p*-values in the testing procedure. When signals are sparse, the TFisher statistic is powerful by including a few smallest *p*-values that are most likely associated with signals; when signals are dense, more *p*-values can be included to improve power. Therefore, the corresponding omnibus testing procedure (the oTFisher) remains robustly high power for various signal patterns by automatically adapting to a subset of important SNPs. Unlike the ACAT-O, which involves different types of test statistics, the oTFisher restricts to the same family of statistics. The adaptation is through truncating and weighting SNP *p*-values, which provides a vehicle for screening important SNPs. If the SNP-set is significantly associated, the important SNPs selected by oTFisher are likely trait relevant. This feature is useful for two reasons. First, important SNP screening based on the SNP-set test could help to identify SNPs with weak genetic effects because the SNP-set test has the potential to detect the totality of genetic effects that single-SNP analysis cannot. Second, the important SNPs that drive the association of a SNP set, e.g., a gene, could help reveal genetic architecture, disease mechanism, and other downstream analyses of the gene.

The exact distribution of oTFisher is challenging to obtain when SNP *p*-values are dependent because of the LD among SNPs. For controlling the type I error rate, we could rely on a re-sampling-based strategy to get the empirical *p*-value of the oTFisher. However, this strategy is computationally expensive, especially for moderate to large SNP sets. We design an efficient algorithm to calculate the *p*-value of the TFisher and the oTFisher. It is a hybrid of the generalization of Brown’s method (GB) (Brown, 1975) and a more advanced skewness-kurtosis-ratio matching method (SKRM) (Zhang and Wu, 2022b; Zhang et al., 2022). The GB is fast and reasonably accurate for larger *p*-values (≥0.01). The SKRM can significantly improve calculation accuracy for smaller *p*-value.

The oTFisher is shown robustly powerful through extensive simulations. The type I error rate is adequately controlled even at a stringent significance level. We applied the oTFisher to analyze the summary data from the Genetic Factors for Osteoporosis Consortium (GEFOS). The oTFisher systematically identified more literature disease genes than the current *p*-value combination methods. The results contributed more insights into the genetics of osteoporosis.

## 2 Materials and methods

### 2.1 SNP-set testing statistics

Let a set of *n* SNPs have *p*-values *P*_*i*_, *i* = 1, *…* , *n*. The TFisher statistic tests the genetic association between a trait and the SNP set by combining these *p*-values while allowing for a general scheme of truncation and weighting (Zhang et al., 2020b):

$$T_{n}\left(\tau_{1},\tau_{2}\right)=-2\overset{n}{\underset{i=1}{\sum }}\log\left(\frac{P_{i}}{\tau_{2}}\right)I\left(P_{i}\leq \tau_{1}\right),$$ (1)

where *I* () is the indicator function, *τ*_1_ > 0 is a truncation parameter that includes *p*-values equal or smaller than *τ*_1_ into the statistic, and *τ*_2_ > 0 is a weighting parameter for selected *p*-values. When *τ*_1_ = *τ*_2_ = 1, the TFisher statistic combines all *p*-values, which is the classic Fisher’s combination statistic: $T_{n}\left(1,1\right)=-2\sum_{i=1}^{n}\log\left(P_{i}\right)$ . The TFisher family include the statistic of truncation-product method (TPM) (Zaykin et al., 2002, 2007): $T_{n}\left(\tau_{1},1\right)=-2\sum_{i=1}^{n}\log\left(P_{i}\right)I\left(P_{i}\leq \tau_{1}\right)$ , i.e., a special case of the TFisher with *τ*_2_ = 1. Our previous study has shown that statistical power and computation efficiency can be improved by weighting the truncated *p*-values through *τ*_2_. An optimality can be reached at *τ*_1_ = *τ*_2_ = *τ* ∈ (0, 1], which gives the soft-thresholding statistic:

$$T_{n}^{s}\left(\tau \right)=2\overset{n}{\underset{i=1}{\sum }}\max\left\{-\log\left(\frac{P_{i}}{\tau }\right),0\right\}.$$

An analogous version of the TPM is the rank-truncation product (RTP) method (Dudbridge and Koeleman, 2003). Let *P*_(1)_ ≤ ⋯ ≤ *P*_(*n*)_ be the ordered input *p*-values. The RTP statistic is $RTP=-2\sum_{i=1}^{k}\log\left(P_{\left(i\right)}\right)$ for some predetermined *k*. The RTP statistic can also be written in consistency with the TPM with *τ*_1_ = *P*_(*k*)_. Calculating the *p*-value of the RTP is more challenging, especially for SNP *p*-values are dependent due to the LD.

The TFisher is a flexible framework to maximize the detection of SNP-set associations over a broad spectrum of signal patterns. Different signal patterns are in favor of different truncating and weighting parameters. For example, when association signals are dense, more SNP *p*-values should be included in the test statistic by large *τ*_1_ and *τ*_2_ so that the test is closer to Fisher’s method. Dense signals happen under the polygenic model with a substantial number of causal SNPs, or when the LD is strong so that many SNPs in LD with the causal SNPs also show association signals. On the other hand, if association signals are sparse (i.e., only a small number of SNP *p*-values are linked to the causal genetic factor), the smallest SNP *p*-values should be included in the statistic by small *τ*_1_ and *τ*_2_.

In reality, the signal patterns are often unknown and substantially differ over traits and loci. Therefore, we rely on the data-adaptive omnibus testing procedure to automatically select appropriate parameters. Specifically, we consider a discrete search domain over {(*τ*_1*k*_, *τ*_2*k*_), *k* = 1, *…* , *K*}, where *K* is the total number of *τ* values to search on. Denote *P*(*k*) the test *p*-value of *T*_*n*_ (*τ*_1*k*_, *τ*_2*k*_). The omnibus statistic is defined as the smallest *P*(*k*), which indicates the maximal association evidence for the whole SNP-set:

$$\text{oTFisher\_minp}=\underset{k=1,\ldots ,K}{\min}P\left(k\right).$$ (2)

Moreover, we define a second omnibus test by Cauchy combination test (CCT) of *P*(*k*)’s (Liu and Xie, 2020):

$$\text{oTFisher\_cct}=\frac{1}{K}\overset{K}{\underset{k=1}{\sum }}\tan\left(\left(0.5-P\left(k\right)\right)\pi \right).$$ (3)

The summands of oTFisher_cct are the transformation of *P*(*k*)’s by the inverse cumulative distribution function (CDF) of the standard Cauchy distribution. Because of the heavy tail of Cauchy distribution, oTFisher_cct is dominated by *p*-values closer to 0 or 1. In practice, we truncate *P*(*k*) = 1 to be 0.9 so that oTFisher_cct is dominated by small *p*-values and performs similarly to oTFisher_minp. Note that since *P*(*k*) depends on the LD (see its calculation below), the oTFisher implicitly accounts for the LD information.

### 2.2 SNP-screening procedures

We can utilize the oTFisher as a procedure to screen for important SNPs. The oTFisher procedure has three steps:

1) SNP-set testing: Identify the significantly associated SNP-sets by their oTFisher *p*-values ≤α/g , where *α* is the adjusted significance level, *g* is the number of SNP sets (e.g., genes) studied simultaneously.
2) Screening: From the *t* SNPs contained in the significant SNP sets, get *s* candidate SNPs with their *p*-value less than a threshold *p* ^⋆^.
3) Validation: Use an independent data to get new *p*-values of the *s* candidate SNPs. Get *s*_1_ validated SNPs with their *p*-values less than *α*/*s*.

A natural choice of the threshold is *p* ^⋆^ = τ*, where $\tau^{*}\equiv \tau_{1k^{*}}$ corresponding to the oTFisher_minp in (2) (i.e., *P* (k*) is the minimal *P*(*k*)). Meanwhile, *P*(*k*) could have similar values over different *k*. To be conservative and reduce the false discoveries, we recommend *p* ^⋆^ = min{τ*, 0.1} (denoted by oTFisher_r as a restricted version).

In practice, SNP screening is commonly based on the Bonferroni procedure or the Benjamini–Hochberg (BH) procedure:

• Bonferroni procedure: The screened SNPs are those with their *p*-values less than *p* ^⋆^ = *α*/*L*, where *L* is the total number of SNPs.
• BH procedure: The screened SNPs are those with *p*-value less than $p^{⋆}=P_{\left(k^{⋆}\right)}$ , where *k* ^⋆^ is the largest *k* such that the ordered SNP *p*-values *P*_(*k*)_ ≤ *αk*/*L*.

The SNPs screened by Bonferroni and BH are validated in the same way as the validation stage for the oTFisher.

There are two potential benefits of utilizing the oTFisher procedure over Bonferroni and FDR procedures. First, as a set-testing method, the oTFisher can potentially increase the discovery of weakly associated SNPs. It is because the SNP-set test can detect the collective existence of weak genetic effects that are indistinguishable from individual SNPs (Donoho and Jin, 2004; Wu et al., 2014; Jin and Ke, 2016). Therefore, the oTFisher could better reveal SNPs with weak genetic effects than the Bonferroni and FDR procedures, which only rely on individual SNP tests. Second, τ* is influenced by the proportion of genetic signals (Zhang et al., 2020b). This information could also contribute to identifying important SNPs.

### 2.3 Algorithms for *p*-value calculation

Following the literature (Brown, 1975; Zhang and Wu, 2022b), we account for the dependence of SNP *p*-values by assuming that the vector of their z-score statistics $Z={\left(Z_{1},\ldots ,Z_{n}\right)}^{\prime }$ is approximately normal:

$$Z∼N\left(\mu ,\Sigma \right),$$ (4)

where the mean vector *μ* corresponds to the association hypotheses: *H*_0_: ***μ*** = **0**, i.e., no SNPs are associated, and *H*_1_: ***μ*** ≠ **0**, i.e., at least one SNP is associated. The correlation matrix **Σ** is assumed to be estimable but otherwise arbitrary. These assumptions are reasonably satisfied in practice when the sample size is reasonably large (e.g., by the linear model-based association tests (Shao, 2010)). As one example, the estimation of **Σ** among the marginal score statistics is given in Section 3.1. For analyzing GWAS summary data where the individual-level genotype data are unavailable, **Σ** can often be estimated by the LD matrix based on reference genome panel data, such as the 1,000 Genome and the UK10K projects (Hu et al., 2013). Although most GWAS summary data contains two-sided *p*-values, we allow they are one-sided for the completeness of statistical development:

$$\text{ One}-\text{sided: }P_{i}=\bar{\Phi }\left(Z_{i}\right);\;\text{ Two}-\text{sided: }P_{i}=2\bar{\Phi }\left(\left|Z_{i}\right|\right),$$ (5)

where $\bar{\Phi }\left(x\right)=P\left(N\left(0,1\right)>x\right)$ denotes the survival function of *N* (0, 1).

### 2.3.1 *p*-value calculation for TFisher

At given *τ*_1_, *τ*_2_ and *n*, the TFisher statistic *T*_*n*_ (*τ*_1_, *τ*_2_) in Eq. 1 has a point probability mass at 0: $p_{0}=P\left(T_{n}\left(\tau_{1},\tau_{2}\right)=0\right)=P\left(\min_{i}P_{i}>\tau_{1}\right)$ corresponding to all *p*-values are truncated. Define *s* = −2 log (*τ*_1_/*τ*_2_) ≥ 0 when *τ*_2_ ≥ *τ*_1_, and *s* = −2*n* log (*τ*_1_/*τ*_2_) < 0 when *τ*_2_ < *τ*_1_. In either case *T*_*n*_ (*τ*_1_, *τ*_2_) ≥ *s* and its distribution is a mixture of point mass at 0 and a continuous distribution defined in [*s*, *∞*). That is, *T*_*n*_ (*τ*_1_, *τ*_2_) ∼ *p*_0_ ⋅ 0 + (1 − *p*_0_) ⋅ *T*′, where *T*′ denotes an appropriate continuous random variable.

The exact value of *p*_0_ is easy to calculate under normality in Eq. 4. The exact distribution of *T*′ is challenging to obtain. We propose to use the gamma distribution model to approximate it for a few reasons. First, the model is consistent with the distribution of the TFisher under independence, which is a weighted gamma distribution (Zhang et al., 2020b). Second, the literature has been using gamma distribution to approximate Fisher’s method under dependence (Brown, 1975; Zhang and Wu, 2022b). Third, when the shape parameter of the gamma distribution is large, it converges to the normal distribution, which is appropriate for the TFisher statistic when *n* is large (see details below). Overall, gamma distribution provides a flexible and straightforward distribution model, vital for computational speed and accuracy.

Specifically, we approximate the distribution of the TFisher statistic by

$$T_{n}\left(\tau_{1},\tau_{2}\right)\overset{D}{\approx }X∼p_{0}\cdot 0+\left(1-p_{0}\right)\cdot X^{\prime },$$ (6)

where *X*′ − *b* ∼Γ(*a*, *θ*), the gamma distribution with shape parameter *a* and scale parameter *θ*. We consider a shift parameter *b* so that *X*′ ∈ [*b*, *∞*). Let *F*_Γ(*a*,*θ*) (_ *x*) denote the CDF of Γ(*a*, *θ*). The CDF of *X* is

$$F_{X}\left(x\right)=P\left(X\leq x\right)=p_{0}I\left(x\geq 0\right)+\left(1-p_{0}\right)F_{\Gamma \left(a,\theta \right)}\left(x-b\right).$$ (7)

Based on (7), the *p*-value of the TFisher at an observed statistic *t* is

$$P\left(T_{n}\left(\tau_{1},\tau_{2}\right)>t\right)\approx 1-F_{X}\left(t\right).$$ (8)

We discuss the methods to calculate *F*_*X*_(*t*) in the following.

Method 1: The generalized Brown’s method (GB): This method follows the essential idea of Brown’s method (Brown, 1975) to match the first two moments of *T* and *X*. Specifically, we set the shift parameter *b* = *s* so that *X*′ and *T*′ have the same domain. The parameters *a* and *θ* are determined by matching the means and the variances of *T*_*n*_ (*τ*_1_, *τ*_2_) and *X*. Denote *μ*_*T*_ = E (*T*_*n*_ (*τ*_1_, *τ*_2_)) and $\sigma_{T}^{2}=Var\left(T_{n}\left(\tau_{1},\tau_{2}\right)\right)$ . We have

$$\left\{\begin{array}{c} \mu_{T}=\mu_{X}=\left(1-p_{0}\right)\left(a\theta +b\right)\;\\ \sigma_{T}^{2}=\sigma_{X}^{2}=\left(1-p_{0}\right)\left(a\theta^{2}+p_{0}{\left(a\theta +b\right)}^{2}\right)\; \end{array}\right.\Rightarrow \left\{\begin{array}{c} a=\frac{{\left(\mu_{T}-b\left(1-p_{0}\right)\right)}^{2}}{\left(1-p_{0}\right)\sigma_{T}^{2}-p_{0}\mu_{T}^{2}}\;\\ \theta =\frac{\left(1-p_{0}\right)\sigma_{T}^{2}-p_{0}\mu_{T}^{2}}{\left(1-p_{0}\right)\left(\mu_{T}-b\left(1-p_{0}\right)\right)}.\; \end{array}\right.$$

Note that the gamma approximation is consistent with the asymptotic normal distribution of *T*_*n*_ (*τ*_1_, *τ*_2_) for large *n* by the Central Limit Theorem (CLT). Specifically, when *n* → *∞*, *p*_0_ → 0, so *μ*_*T*_ = *μ*_*X*_ ≈ *aθ* + *b* and $\sigma_{T}^{2}=\sigma_{X}^{2}\approx a\theta^{2}$ . Because Γ(*a*, *θ*) ≈ *N* (*aθ*, *aθ* ^2^) for large *a*, the distribution model in (6) leads to $T_{n}\left(\tau_{1},\tau_{2}\right)\overset{D}{\approx }\Gamma \left(a,\theta \right)+b\approx N\left(a\theta +b,a\theta^{2}\right)\approx N\left(\mu_{T},\sigma_{T}^{2}\right)$ . However, for finite *n*, the distribution model in Eq. 6 is more accurate for *p*-value calculation.

Straightforward calculation gives *μ*_*T*_ = 2*nτ*_1_ (1 − log  *τ*_1_ + log  *τ*_2_). For the variance $\sigma_{T}^{2}$ , we deduce its analytical formula given in Lemma 1 in Supplementary Material. The formula involves a summation of infinite terms. However, in practice, a summation of two or three terms over *k* would give sufficient accuracy for $\sigma_{T}^{2}$ (Zhang and Wu, 2022b). The proof is based on Mehler’s theorem (Patel and Read, 1996) and is given in Supplementary Material.

Method 2: Skewness-kurtosis-ratio matching method (SKRM). Accurate calculation of small *p*-value highly depends on the precise approximation of the right tail of the null distribution. In this method, we do not require the shifting parameter *b* = *s* but treat it as additional freedom to capture the right-tail information of the TFisher statistic. That is, in addition to the first two moments, we further match the skewness-kurtosis ratios of *T*_*n*_ (*τ*_1_, *τ*_2_) and *X*. Engaging higher-order moments could provide more flexibility in the distribution and thus improve the accuracy of *p*-value calculation. In particular, matching the skewness-kurtosis ratios is a cost-efficient method–it captures two higher moments using only one extra parameter.

Specifically, let $\gamma_{T^{\prime }}=E{\left(T^{\prime }-\mu_{T^{\prime }}\right)}^{3}/\sigma_{T^{\prime }}^{3}$ and $\kappa_{T^{\prime }}=E{\left(T^{\prime }-\mu_{T^{\prime }}\right)}^{4}/\sigma_{T^{\prime }}^{4}$ be the skewness and kurtosis of *T*′, and *γ*_*X*′_ and *κ*_*X*′_ be the skewness and kurtosis of *X*′, respectively. By matching the ratio between skewness and excess kurtosis

$$\frac{\gamma_{T^{\prime }}}{\kappa_{T^{\prime }}-3}=\frac{\gamma_{X^{\prime }}}{\kappa_{X^{\prime }}-3},$$

we can obtain a simple closed form $a=\frac{9\gamma_{T^{\prime }}^{2}}{{\left(\kappa_{T^{\prime }}-3\right)}^{2}}$ . Subsequently, by matching the mean and variance *μ*_*T*′_ = *μ*_*X*′_ and $\sigma_{T^{\prime }}^{2}=\sigma_{X^{\prime }}^{2}$ , we have $\theta =\frac{\sigma_{T^{\prime }}}{\sqrt{a}}$ and $b=\mu_{T^{\prime }}-\sigma_{T^{\prime }}^{2}\sqrt{a}$ . After *a*, *θ* and *b* are determined, the *p*-value of the TFisher can be calculated by Eqs 7, 8.

Exact calculation of *γ*_*T*′_ and *κ*_*T*′_ would be intricate due to the complexity of the high moments of the summational terms in the TFisher statistic. We rely on simulation by Eq. 4 to obtain these values. The number of simulations needed for estimating these parameters is much smaller than that required for obtaining a small empirical *p*-value directly. Therefore, the SKRM is still computationally more efficient than getting *p*-values solely based on the re-sampling strategy.

Method 3: Hybrid method. To balance computational speed and accuracy, we recommend a simple two-stage calculation of TFisher’s *p*-value. Because the GB is fast and accurate for controlling the type I error rate at *α* ≥ 0.01 (see the numerical results below), the GB is applied in the first stage. If the obtained *p*-value is less than 0.01 (the chance is about 1% under the null), the SKRM method will obtain the final *p*-value in the second stage. With a single core of 2.80 GHz AMD EPYC 7543 CPU and 20G memory, the computation times for calculating TFisher’s *p*-values for SNP sets of 30/50/100/200 SNPs are about 0.07/0.13/0.30/0.74 s by the GB method (implemented in R version 4.2.0). Correspondingly, the expected times by the hybrid method (assuming 1% chance of engaging the SKRM method that takes 10^5^ simulations to obtain *γ*_*T*′_ and *κ*_*T*′_ values) are 0.09/0.15/0.33/0.78 s.

### 2.3.2 *p*-value calculation for oTFisher

For the oTFisher_minp in Eq. 2, we can apply asymptotic distribution to approximate its *p*-value by

$$1-\Phi_{R}\left({\bar{\Phi }}^{-1}\left(p_{0}\right),\ldots ,{\bar{\Phi }}^{-1}\left(p_{0}\right)\right),$$ (9)

where *p*_0_ is the observed statistic of oTFisher_minp, Φ_**R**_ denotes the CDF of a multivariate normal distribution with mean zero and correlation matrix **R**. We obtain **R** by scaling **Ω**, the covariance matrix of *T*_*n*_ (*τ*_1*k*_, *τ*_2*k*_), *k* = 1, … , *K*, given by Lemma 2 in Supplementary Material. That is, **R** = **ΛΩΛ** with the diagonal matrix $\Lambda =\text{diag}\left\{1/\sqrt{\Omega_{kk}},\;k=1,\ldots ,N\right\}$ .

As for oTFisher_cct in Eq. 3, following the property of the CCT, its distribution is robust to the correlations as long as *T*_*n*_ (*τ*_1*k*_, *τ*_2*k*_)’s are roughly normal distributed. This requirement is justifiable because *T*_*n*_ (*τ*_1*k*_, *τ*_2*k*_) is in the format of summation so that it is roughly normal distributed by the CLT when *n* is moderately large and *τ*_1_ is not too small. Denote the observed statistic by *cct*_*o*_. We directly apply the result by (Liu and Xie, 2020) to approximate its test *p*-value by $P\left(oTFisher\text{\_}cct>cct_{o}|H_{0}\right)\approx \frac{1}{2}-\tan^{-1}\left(cct_{o}\right)/\pi$ . For the computation time of obtaining the oTFisher’s *p*-value, the dominant part is to get the *p*-values of the TFisher statistics *T*_*n*_ (*τ*_1*k*_, *τ*_2*k*_), *k* = 1, … , *K*.

## 3 Simulation studies

### 3.1 Simulation design

Simulations were applied to verify the accuracy of the *p*-value calculation, statistical power, and SNP-screening performance of the oTFisher procedures. The genotype data were generated by the Cosi2 package (Shlyakhter et al., 2014). Specifically, 1,290 haplotypes were generated according to a coalescent model based on chromosome 1 of the European population. Two haplotypes were randomly picked with replacement to form the genotypes of one diploid individual. In each simulation, we obtained SNPs of *N* individuals. Both rare variants (0.05% < MAF < 5%) and common variants (MAF ≥5%) were considered.

We simulated continuous and binary traits by the regression and the logit model, respectively:

$$\begin{array}{c} Y_{k}=G_{k\cdot }^{\prime }\beta +X_{k\cdot }^{\prime }\gamma +\epsilon_{k},\text{ where }\epsilon_{k}\overset{i.i.d.}{∼}N\left(0,1\right),\\ \text{logit}\left(P\left(Y_{k}=1\right)\right)=G_{k\cdot }^{\prime }\beta +X_{k\cdot }^{\prime }\gamma , \end{array}$$ (10)

where *Y*_*k*_ quantifies the phenotypic trait of the *k*th subject, *k* = 1, … , *N*, with the sample size *N*. $G_{k\cdot }={\left(G_{k1},\ldots ,G_{kn}\right)}^{\prime }$ is the genotype vector of *n* SNPs, $X_{k\cdot }={\left(X_{k1},\ldots ,X_{kl}\right)}^{\prime }$ is the vector of *l* controlling covariates. The nonzero elements of $\beta ={\left(\beta_{1},\ldots ,\beta_{n}\right)}^{\prime }$ are the causal genetic effects of the corresponding SNPs. The SNP-set analysis concerns testing the global hypotheses

$$H_{0}:\beta =0\text{ versus }H_{1}:\beta \neq 0.$$

We mimic a balanced case-control study for the binary traits. That is, a large number of outcomes were generated based on the probability of the logit model, then we randomly selected subjects so that the numbers of cases and controls are *N*/2 each.

Based on the simulated data, we calculate the marginal score test statistic following literature (McCullagh and Nelder, 1989; Schaid et al., 2002; Barnett et al., 2017). Specifically, the score of the *i*th SNP is $M_{i}=\sum_{k=1}^{N}G_{ki}\left(Y_{k}-{\tilde{Y}}_{k}\right),\;i=1,\ldots ,n,$ where $\tilde{Y_{k}}$ are the fitted trait values by the maximum likelihood estimation under *H*_0_. It can be shown that under *H*_0_, $M={\left(M_{1},\ldots ,M_{n}\right)}^{\prime }\overset{D}{\to }N\left(0,\Sigma \right),$ as *N* → *∞*. The covariance matrix **Σ** can be estimated by $\hat{\Sigma }=G^{\prime }WG-G^{\prime }WX{\left(X^{\prime }WX\right)}^{-1}X^{\prime }WG,$ where (**G**, **X**) is the design matrix corresponding to (10), and **W** is a diagonal matrix: $W={\hat{\sigma }}^{2}I$ for continuous trait (where ${\hat{\sigma }}^{2}$ is the estimate of the residual variance); $W=\text{diag}\left\{{\tilde{Y}}_{k}\left(1-{\tilde{Y}}_{k}\right),\;k=1,\ldots ,N\right\}$ for binary trait. Each *M*_*i*_ is standardized to get the marginal score statistic $Z_{i}=M_{i}/\sqrt{{\hat{\Sigma }}_{ii}}\overset{D}{\to }N\left(0,1\right)$ as *N* → *∞* under *H*_0_. The correlation matrix of $Z={\left(Z_{1},\ldots ,Z_{n}\right)}^{\prime }$ is estimated by $\hat{D}\hat{\Sigma }\hat{D}$ , where $\hat{D}=\text{diag}\left\{\sqrt{{\hat{\Sigma }}_{ii}},\;i=1,\ldots ,n\right\}$ . Thus, the marginal score statistics satisfy the assumption (4) asymptotically. We used the two-sided SNP *p*-values in Eq. 5. For the rare-variant analysis of binary traits, the saddle point approximation (SPA) was applied to obtain SNP *p*-values, which corrects the bias due to the unbalanced distribution of rare variants’ genotype data (Dey et al., 2017).

### 3.2 Accuracy of *p*-value calculation

Under the null, quantitative and binary trait values were generated by setting ***β*** = **0** and **Z** = **1** in Eq. 10. We simulated 10^7^ oTFisher statistics with the adapting domain $\tau_{1}=\tau_{2}=\tau \in T=\left\{0.001,0.005,0.01,0.05,0.1,0.2,0.5,0.7,1\right\}$ . The simulations included 100 randomly generated genotype data to mimic various minor allele frequencies and LD structures of SNPs in various genes. The empirical type I error rate was obtained by proportionating all calculated *p*-values smaller than a given nominal level *α*. Table 1 lists the ratios of empirical type I error rates and the nominal *α* levels under common and rare variants for quantitative and binary traits. A ratio around 1 indicates accurate calculation. A more stringent *α* is harder to control. The GB method well controls type I error rate up to *α* ≥ 0.005, but becomes liberal at smaller *α*, where the SKRM method (using 10^5^ simulations to obtain the third and fourth moments) controls the error much better. Therefore, the hybrid method combining the GB and the SKRM balances accuracy and computational speed. Consistent simulation results with various *n* and T settings (with starting *τ* = 10^–5^, 10^–3^ or 10^–2^ in T ) are given in Supplementary Tables S1–S4 in Supplementary Material.

**TABLE 1:** Type I error control for SNP-set testing by the oTFisher under quantitative and binary traits. SNP-set size *n* = 100, sample size *N* = 1,000.

*α*	rare variants				common variants			
	oTFisher_cct		oTFisher_minp		oTFisher_cct		oTFisher_minp	
	GB	Hybrid	GB	Hybrid	GB	Hybrid	GB	hybrid
Continuous trait								
0.1	0.93	0.94	0.84	0.85	0.89	0.90	0.83	0.83
0.05	0.98	0.99	0.80	0.81	0.95	0.96	0.77	0.77
0.01	1.15	0.9	0.88	0.64	1.13	0.94	0.84	0.64
0.005	1.27	0.86	1.00	0.57	1.28	0.90	0.97	0.56
0.001	1.80	0.87	1.48	0.56	1.82	0.89	1.50	0.52
0.0005	2.17	0.89	1.84	0.58	2.21	0.91	1.89	0.53
0.0001	3.69	0.95	3.61	0.70	3.66	1.00	3.65	0.73
0.00005	4.82	1.05	4.99	0.87	4.66	1.08	4.96	0.79
0.00001	9.54	1.11	11.00	1.06	8.89	1.25	11.33	1.29
Binary trait								
0.1	0.93	0.93	0.85	0.85	0.88	0.88	0.82	0.82
0.05	0.97	0.98	0.81	0.81	0.94	0.94	0.76	0.76
0.01	1.10	0.86	0.85	0.60	1.10	0.9	0.82	0.61
0.005	1.22	0.81	0.94	0.53	1.23	0.84	0.93	0.52
0.001	1.71	0.75	1.39	0.48	1.75	0.8	1.44	0.46
0.0005	2.07	0.77	1.75	0.50	2.11	0.81	1.82	0.47
0.0001	3.50	0.87	3.38	0.61	3.52	0.85	3.54	0.63
0.00005	4.59	0.86	4.68	0.67	4.53	0.93	4.82	0.71
0.00001	8.94	1.09	10.32	0.98	8.70	1.16	11.09	1.02

### 3.3 Statistical power

Through simulations, we assessed the statistical power of the oTFisher tests in comparison with other *p*-value combination tests in GWAS summary data analysis: the GATES (extended Simes procedure, using GATES2 function in R library aSPU (Li et al., 2011)), the CCT (i.e., the ACAT with equal weights), Fisher’s method, the soft-thresholding TFisher at fixed *τ*_1_ = *τ*_2_ = 0.05, the adaptive TPM (ATPM), and the adaptive RTP (ARTP). The ATPM follows (1) and (2) with fixed *τ*_2_ = 1, and adapts over $\tau_{1}\in T$ . The ARTP adapts over *τ*_1_ ∈ {*P*_(*k*)_}, where k∈nT (rounding to the nearest integers) to be consistent with the oTFisher and the ATPM.

We considered that the causal SNPs were randomly located, and their effect sizes were given by the nonzero elements of ***β*** in Eq. 10. For a fair comparison, we empirically controlled the type I error rate *α* to avoid potentially unavailable or inaccurate *p*-value calculation for some tests. For example, there are no *p*-value calculation methods for the RTP and the ARTP under dependence. We got the critical value of one statistic by the upper 100*α%* percentile of its values generated from 10,000 simulations under the null. The statistical power was obtained by the percentage surpassing the critical value among the statistics generated from 1,000 simulations of the alternative. For the RTP and ARTP, we applied a one-level simulation algorithm consistent with literature (Yu et al., 2009) except that we directly simulated the Z-scores for faster computation instead of permuting the genotype data (details see Supplementary Material).

**FIGURE 1:**
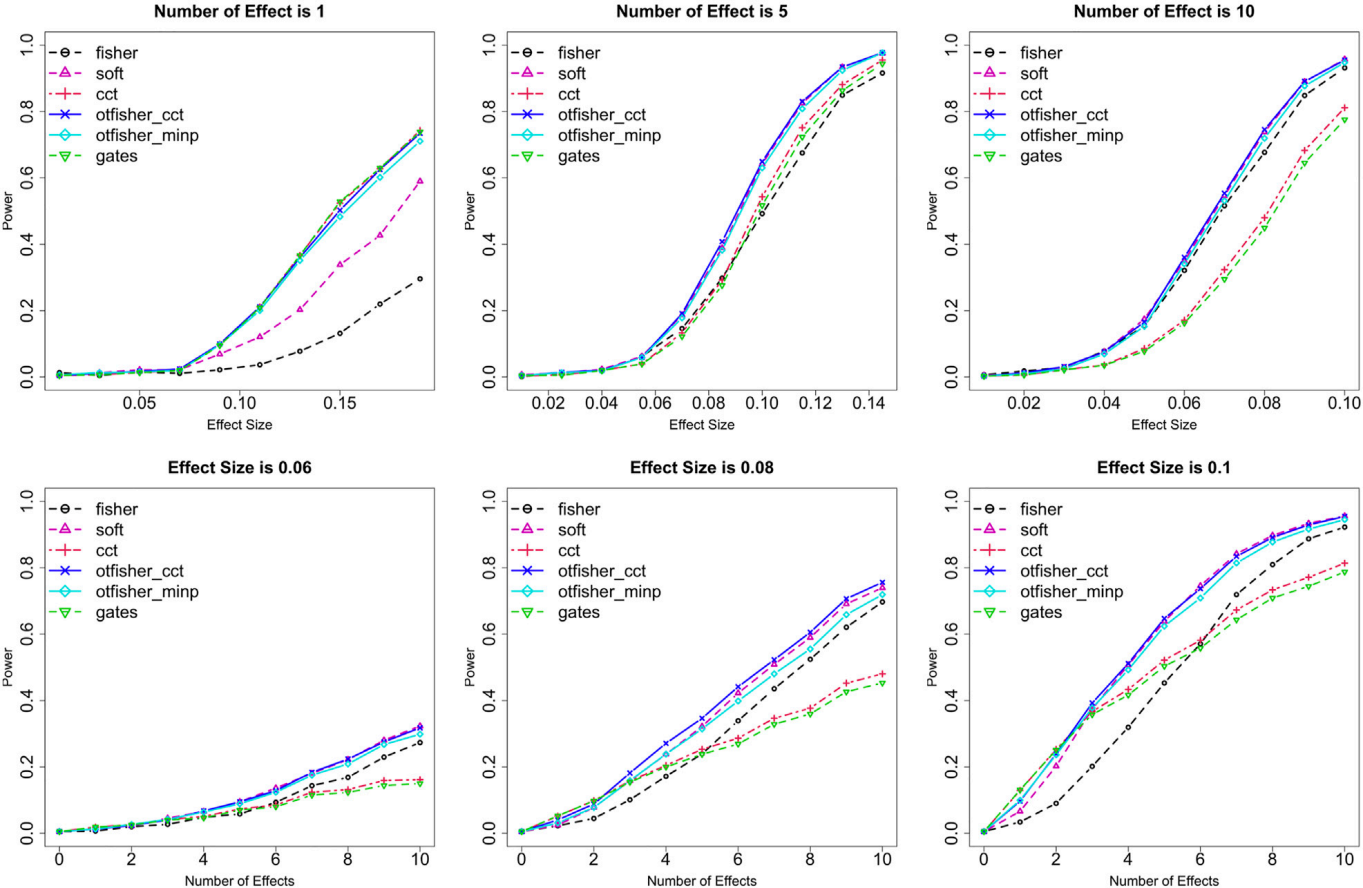
Statistical power for binary traits from common SNPs. Row 1: Fixing the number of causal SNPs *m* = 1, 5, and 10, and varying effect size *β* on *x*-axis. Row 2: Fixing *β* = 0.06, 0.08, and 0.1, and varying *m* on *x*-axis. Testing methods: fisher: Fisher’s method; soft: soft-thresholding TFisher with *τ*_1_ = *τ*_2_ = 0.05; cct: Cauchy combination test; otfisher_minp: oTFisher in Eq. 2; otfisher_cct: oTFisher in Eq. 3; gates: extended Simes procedure.

**FIGURE 2:**
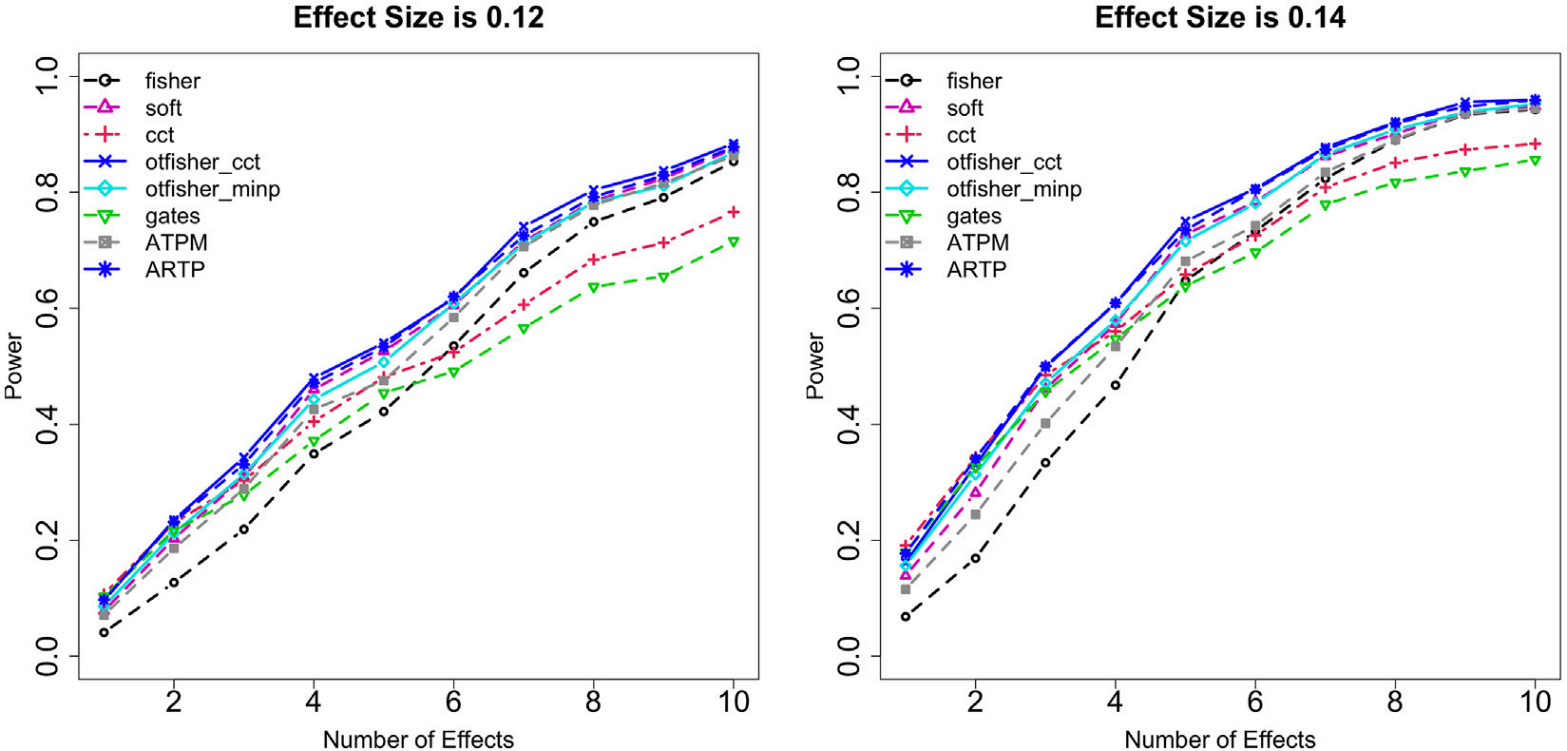
Power comparison among the data-adaptive tests. Quantitative traits from rare variants. *α* = 0.01. Fixing the effect size to be 0.12 (left panel) and 0.14 (right). *X*-axis: The number of causal SNPs. Testing methods: fisher: Fisher’s method; soft: soft-thresholding TFisher with *τ*_1_ = *τ*_2_ = 0.05; cct: Cauchy combination test; otfisher_minp: oTFisher in Eq. 2; otfisher_cct: oTFisher in Eq. 3; gates: extended Simes procedure; ATPM: adaptive TPM; ARTP: adaptive RTP.

Statistical power was systematically studied under various settings regarding trait type (quantitative or binary), SNP type (common or rare), the number of causal SNPs *m*, genetic effect size *β*, SNP-set size *n*, sample size *N*, and type I error rate *α*. Figure 1 shows the power comparison under binary traits from common SNPs at *α* = 0.005 with *n* = 100 and *N* = 10,000. A few interesting observations can be made. First, the GATES and the CCT have similar performances. They are advantageous when causal SNPs are sparse (e.g., *m* ≤ 3) and their effects are strong. Fisher’s method shows an opposite pattern—it is preferred if causal SNPs are dense, especially when effects are weak. These patterns are consistent with literature results (Zhang and Wu, 2022a). TFisher at fixed *τ*_1_ = *τ*_2_ = 0.05 is more robust over sparse and dense causal SNPs. However, it could still be less satisfactory (e.g., when *m* ≤ 3). The oTFisher_cct and oTFisher_minp are similar; their power is the best in most scenarios, showing an overall advantage over unknown genetic architectures. In comparison with the ATPM and the ARTP, Figure 2 shows that the oTFisher is uniformly better than the ATPM. This observation is well-supported by a theoretical optimality study (Zhang et al., 2020b). The ARTP and the oTFisher have very similar power. Meanwhile, our *p*-value calculation algorithm provides a practical advantage for applying the oTFisher over the ARTP in computation. These comparison patterns remain similar for quantitative traits, rare variants, and different *α* levels. Comparisons under other settings are given in Supplementary Figures S1–S13 in Supplementary Material.

### 3.4 SNP screening

We studied the performance of SNP-screening procedures measured by the accuracy of detecting causal SNPs. Because non-causal SNPs in LD with causal SNPs also show statistical associations, the study focused on rare-variant analysis with weak LDs for simplicity. To mimic a gene-based SNP-set analysis, we simulated *L* = 1,000 SNPs (with the LD *r* ^2^ < 0.3) in *g* = 10 genes of equal size. Two causal genes contained causal SNPs with random locations. The continuous and binary traits were obtained using models in Eq. 10 that included all causal SNPs. We systematically varied the genetic effect *β* and the proportion of causal SNPs in the two causal genes. The sample size *N* = 1,000; the cases and controls were balanced for binary traits.

We considered accuracy by the sensitivity, specificity, and balanced accuracy (BA, the average of sensitivity and specificity) based on the true positives (TP, the picked SNPs that are causal), false positives (FP, the picked SNPs that are non-causal), true negatives (TN, the unpicked SNPs that are non-causal), and false negatives (FN, the unpicked SNPs that are causal). These numbers are determined after defining the “picked” and “unpicked” SNPs. At the screening stage of the oTFisher procedure described in Section 2.2, we consider the *s* candidate SNPs as being picked from in total *t* SNPs in the significant genes; the rest *t* − *s* SNPs are unpicked. At the validation stage, the *s*_1_ validated SNPs are picked, and the rest *t* − *s*_1_ SNPs in the significant genes are unpicked. The accuracy measures were averaged over 1,000 simulations.

The oTFisher procedure was compared with Bonferroni and BH procedures for SNP screening. We further considered an oracle procedure:

• Oracle procedure: Assume the number of causal SNPs *m* is known, the oracle, i.e., the best possible, SNP screening procedure is to pick SNPs by setting *p* ^⋆^ = *P*_(*m*)_, the *m*th smallest SNP *p*-value.

Certainly, *m* is unknown in reality, so this procedure is a hypothetically optimal procedure serving as an indicative accuracy for comparison purposes. The validation process is the same for all procedures.

**FIGURE 3:**
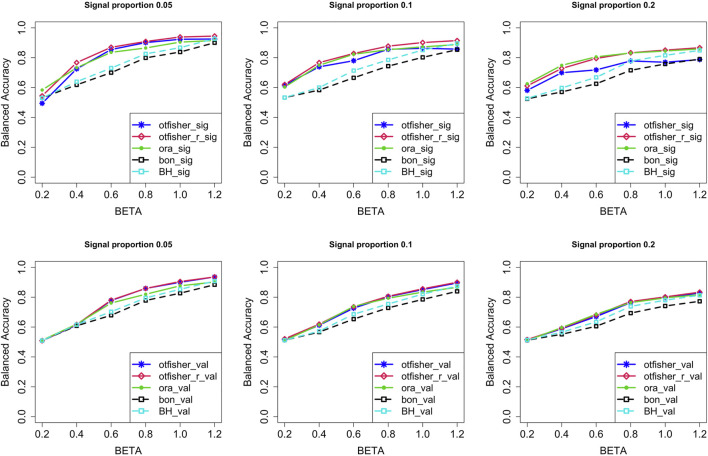
The balanced accuracy at the SNP screening (row 1) and validation (row 2) stages under the continuous trait model. Code for methods: otfisher: the oTFisher procedure with threshold *p* ^⋆^ = τ*; otfisher_r, the oTFisher procedure with *p* ^⋆^ = min{τ*, 0.1}; ora, the oracle procedure; bon, Bonferroni procedure; BH, Benjamini–Hochberg procedure; sig, screening stage; val, validation stage. Signal proportion is the proportion of causal SNPs in the two causal genes.

Figure 3 compares the BA of the screened SNPs and validation of them. Overall, in both screening and validation stages, the oTFisher_r gave a higher BA than Bonferroni and the BH, sometimes even the Oracle. In the screening stage, the oTFisher using p* = τ* had higher sensitivity but lower specificity. Restricting the threshold to *p* ^⋆^ = min{τ*, 0.1} significantly increased the specificity. The validation stage helped further control the type I error. For comparisons of sensitivity and specificity and results under more settings, see Supplementary Figures S14–S21 in Supplementary Material.

## 4 Real-data analysis

We conducted a comprehensive study of nine GWAS summary data sets from the GEFOS (Estrada et al., 2012; Zheng et al., 2015; Kemp et al., 2017; Medina-Gomez et al., 2017; Medina-Gomez et al., 2018; Trajanoska et al., 2018, 2020; Morris et al., 2019). A description of these studies and data is given in Supplementary Material. Using the SNP *p*-values, we carried out gene and haplotype-block (haploblock) analyses for hunting putative genetic factors associated with bone mineral density (BMD) related traits and fall risk. The Supplementary Material gives details on our data pre-processing, including the pipeline to map SNPs to genes and haploblocks (Gabriel et al., 2002; Chang et al., 2015; Deng et al., 2016), correlation estimation by reference genome panel of the 1,000 Genome project (Higham, 2002; Lin and Zeng, 2010b), and SNP *p*-value adjustment based on the LD score regression (Bulik-Sullivan et al., 2015; Lee et al., 2018). For stable numerical computation without losing much associative information, SNPs with high LDs are pruned—if a SNP pair has the LD *r* ^2^ > 0.9, the variants with a lower MAF would be removed (following the default setting of PLINK’s SNP pruning function (Chang et al., 2015)). Furthermore, for GEFOS2017_TBBMD data, it contains a large number of genome-wide significant SNPs (*p*-values < 5E-8). These SNPs were removed from our SNP-set analyses for this data. The purpose is to reduce the false positive rate and study how many genes and haploblocks could still be detected by SNP-set analysis. The Q-Q plots for raw SNP *p*-values are given in Supplementary Figure S22. The summary statistics on the features of SNP, genes, and haploblocks are given in Supplementary Tables S5–S8.

We extensively searched the literature and obtained comprehensive lists of 2,179 “literature” genes and 4,802 literature SNPs reported to be associated with osteoporosis, bone fracture, and various traits of bone mineral density (BMD). For the falling risk, we took the 16 genes reported by (Trajanoska et al., 2020) as literature genes since it is the only large-scale study we found regarding this trait. The searching strategies and resources are described in Supplementary Material (last update: 15 May 2022). The lists are in supplementary files literature_genes.xls and literature_snps.xlsx, including the gene and SNP information, associated phenotypes, resources, references, etc. These literature genes and SNPs are enriched with true disease genetic factors. Therefore, including literature genes and SNPs among the top hits can evidence the credible performance of a good data analysis method. At the same time, top hits that are not among these literature findings but are functionally relevant to the given trait can be reasonably considered as putative novel genetic discoveries.

### 4.1 Gene-based analysis

We studied the genetic associations between genes and traits using the SNP sets grouped by genes. Four methods were applied. First, the Bonferroni procedure represents the single-SNP method applied in the original GEFOS studies. The top-hit genes contained significant SNPs with *p*-values less than Bonferroni adjusted threshold: 0.05 divided by the total number of SNPs in all genes. Second, we applied two broadly applied SNP-set methods using SNP *p*-values: the ACAT (Liu et al., 2019) and the GATES. Third, we applied our proposed oTFisher method with the adapting domain $\tau_{1}=\tau_{2}=\tau \in T=\left\{10^{-5},0.001,0.005,0.01,0.05,0.1,0.2,0.5,0.7,1\right\}$ .

The oTFisher_cct and the oTFisher_minp yielded similar results, so the former is reported below for simplicity. The Q-Q plots of the gene-based association *p*-values are given in Supplementary Figure S24. The top-hit genes are given in the supplementary file top_gene.xlsx. The overlaps of the top-hit genes and the top-hit literature genes among these four analysis methods are given in Supplementary Figures S25–S26.

**FIGURE 4:**
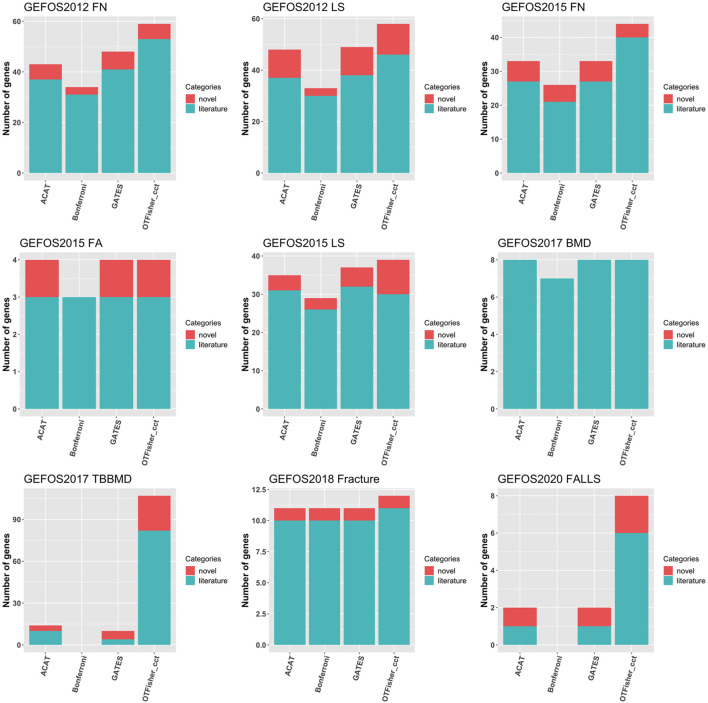
Bar chart for top-hit genes by various SNP-set analysis methods. Note: Bonferroni procedure corresponds to zero genes for GEFOS2017_TBBMD (because genome-wide significant SNPs were removed from the gene-based analysis of this data) and GEFOS2020_FALLS (because no genome-wide significant SNPs were mapped to genes).

Figure 4 summarizes the numbers of top gene hits, from which we can make a few interesting observations. First, the Bonferroni procedure systematically led to fewer top hits and literature genes than the other methods. The result indicates that SNP-set tests could have higher statistical power than single-SNP analysis in detecting disease genes. Second, the oTFisher yielded similar or more gene hits, and most of the hits are literature genes indicating a reliable discovery and potentially higher statistical power. In particular, the oTFisher could have the advantage of detecting polygenic genes that contain relatively dense genetic signals. For examples, the oTFisher detected significantly more genes in the studies of GEFOS2017_TBBMD and GEFOS2020_FALLS, where top-hit genes often contain multiple SNPs with relatively small *p*-values (see Supplementary Tables S9 and S10 for the distribution of SNP *p*-values within these top-hit genes). The polygenic genetic architecture is possible for complex human diseases, including the BMD-related traits (Kemp et al., 2017; Morris et al., 2019).

**FIGURE 5:**
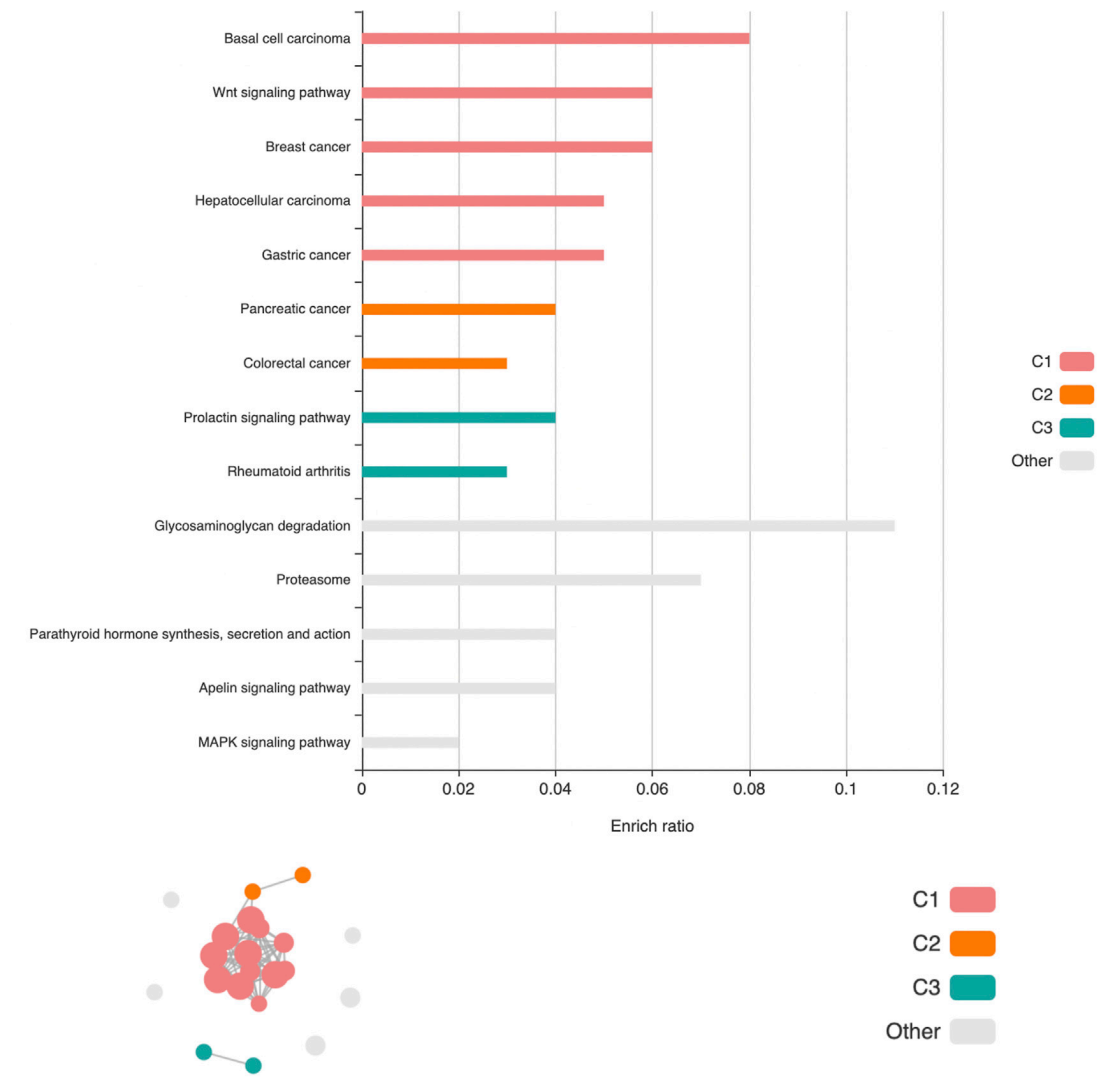
The cirFunMap plot of the pathway network is based on 173 top-hit genes from BMD-related studies. Above: The barplot of the enrichment ratio is defined as the number of top-hit genes in the pathway over the number of total genes in the pathway. Below: The circular network plot. The node color represents different clusters. The node size represents the levels of *p*-value: from small to large: (0.01,0.05), [0.001,0.01), (0.0001,0.001), and (1e-10,0.0001). The edge represents correlations larger than a default threshold of 0.35.

We carried out gene-set enrichment analyses (GSEA) for the top-hit genes identified by oTFisher. Based on gene ontology (GO) and KEGG pathways, the analysis was conducted by KEGG Orthology-Based Annotation System intelligent version (KOBAS-i) (Bu et al., 2021). The analysis identified significant GO terms and biological pathways enriched in the top-hit genes at the corrected significance level of 0.05. The GEFOS data-specific results are summarized in supplementary files top_GOs_pathways_study-specific.xlsx. These GO terms and biological pathways are often related to the bone and skeletal system and are consistent with the osteoporosis pathways reported in literature (Guo et al., 2019). Furthermore, we considered the BMD as a general trait and carried out the GSEA by pooling 173 top-hit genes from eight studies (except for falling risk). Twenty-one significantly enriched pathways were obtained and clustered into three networks based on their correlations by the cirFunMap plot (Bu et al., 2021). Figure 5 visualizes the clusters (ranked by the median *p*-value of the enriched pathways within each cluster). The first cluster gives a major network containing 12 pathways: Wnt signaling pathway (hsa04310), breast cancer (hsa05224), hepatocellular carcinoma (hsa05225), pathways in cancer (hsa05200), gastric cancer (hsa05226), basal cell carcinoma (hsa05217), signaling pathways regulating pluripotency of stem cells (hsa04550), proteoglycans in cancer (hsa05205), hippo signaling pathway (hsa04390), human papillomavirus infection (hsa05165), Cushing syndrome (hsa04934), and mTOR signaling pathway (hsa04150). The top two significant pathways, the Wnt signaling and breast cancer pathways, have been reported in literature (Guo et al., 2019). The large cluster here provides a networking context for them. The second cluster, containing pancreatic cancer (hsa05212) and colorectal cancer (hsa05210), is also connected with the first cluster. The third cluster, containing the prolactin signaling pathway (hsa04917) and rheumatoid arthritis (hsa05323), is independent of the rest. Details of the significant pathways and their clusters for the BMD traits are given in supplementary files top_pathways_BMDs.xlsx.

The top-hit novel genes included in the enriched GO terms and pathways are likely disease genes that influence the corresponding functionalities. In particular, we obtained three top-hit novel genes in the 21 significantly enriched pathways obtained by pooling 173 top-hit genes of the BMD traits. Gene *HSPG2* (chr1: 22148724–22263790, oTFisher *p*-value 1.18E-07) is included in a significantly enriched pathway of proteoglycans in cancer (hsa05205, corrected enrichment *p*-value 0.0047). It was shown to be associated with segregating developmental dysplasia of the hip (Basit et al., 2017). Gene *MAP3K12* (chr12: 53874275–53893444, oTFisher *p*-value 6.49E-09) is included in the significantly enriched MAPK signaling pathway (hsa04010, corrected enrichment *p*-value 0.0048). It is related to lissencephaly type 3 - metacarpal bone dysplasia and infantile osteopetrosis with neuroaxonal dysplasia in the Open Targets Genetics (Ghoussaini et al., 2021). Gene *PRKAG1* (chr12: 49396054–49412629, oTFisher *p*-value 2.22E-06) is included in the significantly enriched Apelin signaling pathway (hsa04371, corrected enrichment *p*-value 0.0049). It is related to bone marrow failure syndrome in the Open Targets Genetics. Novel top-hit genes contained in the enriched GOs and pathways from GEFOS data-specific results are summarized in the supplementary file novel_genes_in_top_GOs_pathways_study-specific.xlsx. More discussion of them is given in Supplementary Material, including genes connected to relevant traits such as osteoarthritis, osteosarcoma, and bone metastasis.

### 4.2 Haplotype block-based analysis

**FIGURE 6:**
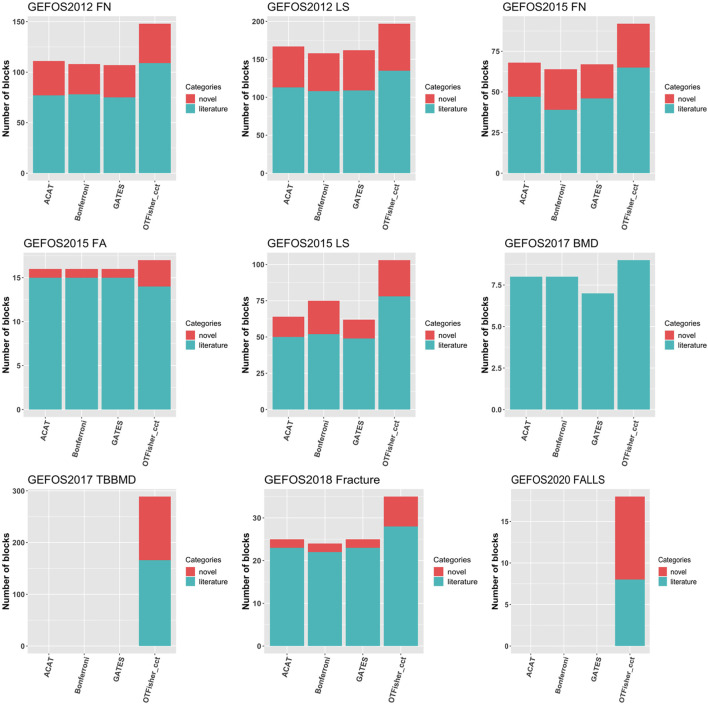
Bar chart for top-hit blocks by various tests. Literature blocks are those mapped to literature genes or containing literature SNPs. Genome-wide significant SNPs were removed from testing the data of GEFOS2017_TBBMD. There are no detections for ACAT and GATES for the 2017_TBBMD and 2020_FALLS data because they had no haploblock *p*-values surpassing the significance level defined by 0.05 over the number of blocks.

Gene-based analysis has the limitation of a small coverage of the genome. For a whole-genome association study, we grouped and analyzed SNPs by haplotype blocks (haploblock estimation by PLINK (Chang et al., 2015) is detailed in Supplementary Material). The Q-Q plots and the Manhattan plots are given in Supplementary Figures S27 and S28 in Supplementary Material. Overall, genomic inflation is reasonably controlled. For various SNP-set methods, Figure 6 shows the number of top-hit blocks. Compared with other methods, the oTFisher generated more top-hit blocks and novel blocks (i.e., top-hit blocks that do not overlap literature genes or SNPs). More details on the top-hit blocks and their corresponding SNPs and genes are summarized in the supplementary file top_haploblocks.xlsx.

The haploblock-based analysis provided complementary results to the single-SNP analysis and gene-based analysis. As a SNP-set analysis method, haploblock analysis could detect additional disease SNPs over single-SNP analysis. For example, in the study GEFOS2012_FN, the top-hit blocks by the oTFisher discovered 56 literature SNPs that the single-SNP analysis failed to detect (since their *p*-values do not pass the genome-wide significance level). The haploblock analysis could also map additional disease genes over the gene-based analysis. Supplementary Table S11 summarizes the numbers of top-hit blocks that can map to literature or novel genes. According to the results, the haploblock analysis found some literature genes that were not among the top hits of the gene-based analysis. For example, in the study GEFOS2012_FN, six literature genes were mapped by top-hit haploblocks but were not discovered by the gene-based analysis: *ATXN7L3*, *AXIN1*, *CPED1*, *FUBP3*, *LOC100272217*, and *SOX6*. For all GEFOS studies, we had 27 literature genes and 119 literature SNPs (63 of them are outside gene regions) detected by haploblock analysis but not by gene-based analysis. Supplementary Figure S29 lists the numbers of literature genes and SNPs found by haploblock analysis versus gene-based analysis. Furthermore, the top-hit blocks (including single-SNP blocks) contained all GEFOS-reported significant SNPs, indicating no information lost compared to the original GEFOS studies.

The top-hit blocks from eight BMD studies contain 286 novel blocks; 255 of them are outside of genes (detailed information is given in the supplementary file novel_haploblocks.xlsx). By epigenetic annotation (Haploreg v3 (Ward and Kellis, 2016)), 58 of the novel blocks (representing 43 non-overlapping loci) co-locate with strong enhancers of literature genes. Therefore, these novel blocks are of interest due to their functional connections. For example, a top-hit block chr2:54643778-54645650 (oTFisher *p*-value 1.67E-16 by GEFOS2012_LS data) contains three SNPs rs13393949 (*p*-value 2.12E-06), rs4671215 (*p*-value 9.36E-08), and rs7560205 (*p*-value 1.72E-05) that locate at a strong enhancers of gene *SPTBN1* in cells Huvec (umbilical vein endothelial cells) and NHEK (epidermal keratinocytes). Gene *SPTBN1* is shown associated with heel bone mineral density (Kemp et al., 2017). The enhancer-located novel blocks are given in the supplementary file novel_haploblocks_enhancers_literaturegene.xlsx, and an extensive discussion of the related SNPs is given in Supplementary Material.

### 4.3 Screening SNPs

**FIGURE 7:**
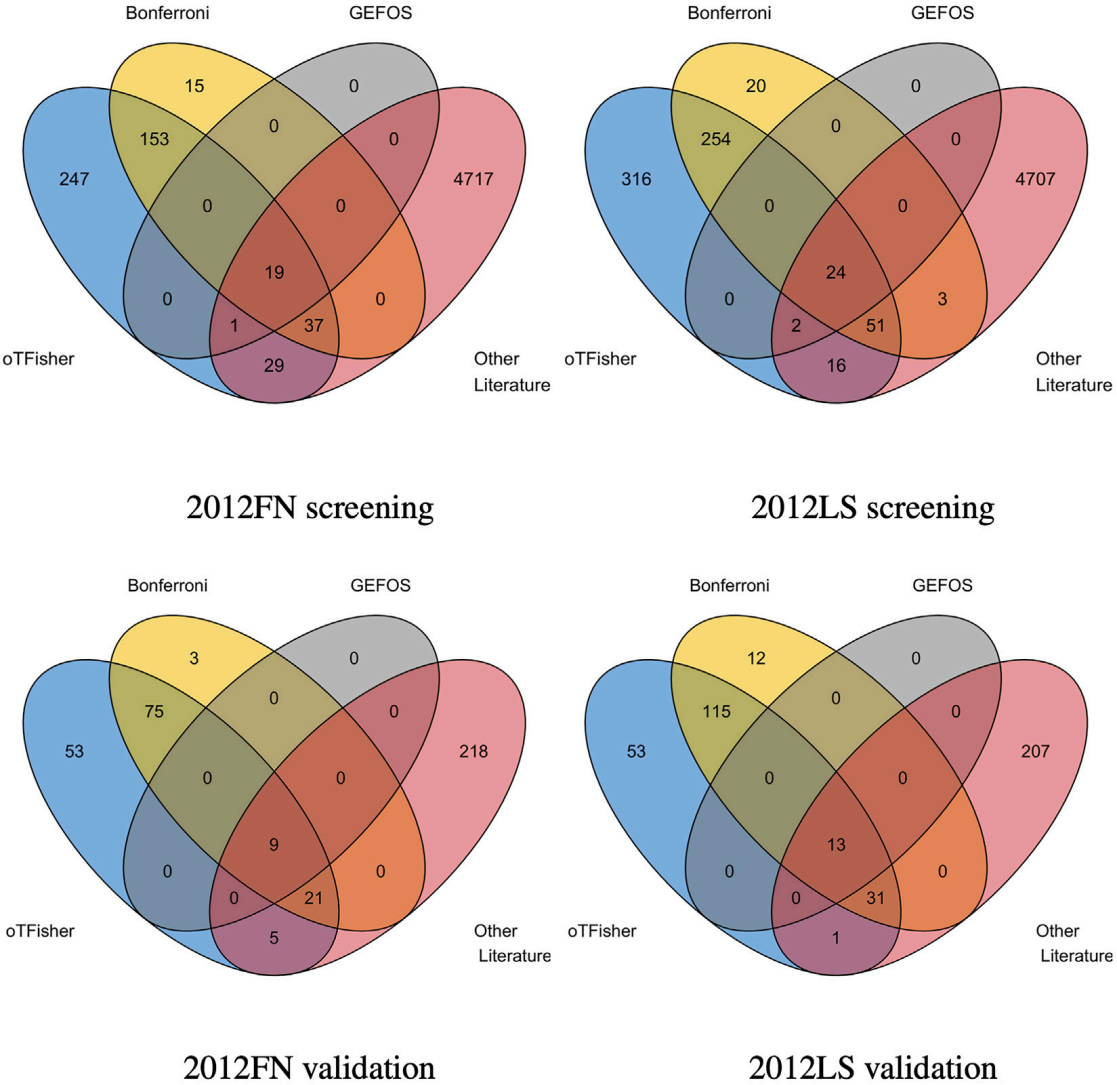
Overlap the screened SNPs in haploblock-based analysis (row 1) and validated SNPs (row 2) based on the oTFisher_r, Bonferroni procedure, original GEFOS study, and literature. SNP screening is done by GEFOS2012_FN (left column) and GEFOS2012_LS (right column); validation is done by the UK Biobank data.

Real-data analysis results show that the SNPs-screening procedure by the oTFisher_r could likely yield more disease SNPs than the Bonferroni procedure. Specifically, we used GEFOS2012 data sets to collect screened SNPs and validate them by a large data of osteoporosis from the UK Biobank (15,133 cases and 426,942 controls at ages of 38–73 years) (Sudlow et al., 2015). Figure 7 shows the Venn diagrams of the screened SNPs by the oTFisher_r and the Bonferroni procedures in the haploblock-based analyses, which are also compared with the SNPs reported in the original GEFOS studies and the literature SNPs. For consistent comparison, the validated SNPs were defined by the significance level of 0.05 over the total number of unique SNPs from the screening step and the literature. As expected, all GEFOS-reported SNPs were contained by the sets of literature SNPs as well as the screened SNPs. The oTFisher_r replicated more literature SNPs than the Bonferroni both before and after the validation stage. For example, with GEFOS2012_FN data, all screened SNPs by the Bonferroni were included in the set of SNPs by the oTFisher_r, while the oTFisher_r screened 30 additional literature SNPs (among which five were validated). Therefore, the oTFisher_r likely has a higher chance of finding disease SNPs. Furthermore, over 30% of the screened SNPs by oTFisher_r were verified. The high validation percentage (compared to the expected percentage of no more than 5% under the null) indicates that the set of screened SNPs by oTFisher_r likely contains enriched disease SNPs. Consistent results for gene-based analysis are given in Supplementary Figures S30 and S31 in Supplementary Material.

## 5 Discussion

GWAS summary data is a rich resource for hunting genetic factors associated with the susceptibility of human complex diseases. To facilitate analyzing such data, in this paper, we propose a SNP *p*-value combination test, the oTFisher, which has robustly high statistical power through adapting to unknown patterns of genetic effects. We develop computationally efficient algorithms to calculate the *p*-value of the oTFisher, which account for the LD of the SNPs. One advantage of such *p*-value combination test is that they do not assume a special type of SNP statistics. In principle, the same calculation can be carried out as long as the correlations among the SNP statistics can be estimated.

As given in Eq. 2, the oTFisher’s truncating and weighting scheme for SNP *p*-values maximizes the significance of the potential underlying genetic effects (through minimizing the corresponding TFisher’s *p*-value). With well-controlled type I error rate of the oTFisher, this automatic truncating scheme could serve as a vehicle for screening important SNPs that contribute to the overall association of the given SNP set. Results show that this screening procedure could better identify disease SNPs than the traditional Bonferroni and FDR procedures. Meanwhile, because the screening procedure is relatively liberal, validating these screened SNPs using an independent high-quality data set is critical for controlling false positives. Furthermore, one should always be cautious about interpreting the screened SNPs in the sense that statistical association does not necessarily mean causality. The associated SNPs could be due to LD with causal SNPs or even confounding effects. Nevertheless, the oTFisher provides a new way of exploring important SNPs not from their individual perspective but from the combined effects of the group as a whole.

We applied the oTFisher to a comprehensive study of osteoporosis-related traits using GEFOS data. Besides demonstrating the merit of the new method, we also generated novel genes and haploblocks that could benefit the downstream study of osteoporosis genetics. Further biological validations of these results are desired.

Our GEFOS data analysis focused on gene and haplotype block-based SNP-grouping strategies for simple biological interpretability. Based on the data-adaptive omnibus testing principle, the oTFisher can also be extended to other SNP-grouping as well as annotation-weighting strategies in whole genome sequencing studies, especially for studying the noncoding regions, following the ideas proposed in recent literature (Morrison et al., 2017; Li et al., 2020, 2019, 2022).

In general, the quality of GWAS summary data analysis highly depends on the quality of the input data. For example, if the SNP *p*-values were inflated, the subsequent SNP-set testing results will be inflated. Current inflation correction procedures could partially address the problem but are still limited. Further research in this direction is needed. Indeed, high-quality data is essential; we highly appreciate data-generating studies providing high-quality summary data for the sake of both primary and secondary data analyses.

## Data Availability

The original contributions presented in the study are included in the article/Supplementary Material, further inquiries can be directed to the corresponding author.
