# Supplementary material for: Simultaneous detection of novel genes and SNPs by adaptive p-value combination
Source: Front Genet. 2022 Nov 17;13:1009428. doi: 10.3389/fgene.2022.1009428 (PMC9713700; doi:10.3389/fgene.2022.1009428)
Supplement: Supplementary file 1 [file DataSheet2.PDF]

Supplementary Material

1 MATERIALS AND METHODS

1.1 Lemma 1

LEMMA 1. *Write* $P_i = 1 - F(Z_i)$*, where* $F(x) = \Phi(x)$ *for one-sided* P_i*'s or* $F(x) = F_{\chi_1^2}(x^2)$ *for two-sided* P_i*'s. Denote* $\rho_{ij} = cor(Z_i, Z_j)$*. We have*

$$\sigma_T^2 = \sum_{1 \le i,j \le n} \sum_{k=1}^{\infty} \frac{\rho_{ij}^k}{k!} I_i(k) I_j(k), \tag{S1}$$

where $I_j(k) = \int_{F^{-1}(1-\tau_1)}^{\infty} \phi(x) G(x|\tau_2) H_k(x) dx$*,* $G(x|\tau_2) = -2\log\left(\frac{1-F(x)}{\tau_2}\right)$*, and* $H_k(x)$ *is the* k*-th order Hermite polynomial.*

PROOF OF LEMMA 1. Write $T(\tau_1, \tau_2) = \sum_{i=1}^{n} Y_i$, where $Y_i = -2\log\left(\frac{P_i}{\tau_2}\right) I(P_i < \tau_1)$, $i = 1, \cdots, n$, have the same marginal distribution under the null hypothesis. Then,

$$\sigma_T^2 = \sum_{1 \le i,j \le n} \mathrm{Cov}(Y_i, Y_j).$$

When $i = j$, it is straightforward get $\mathrm{Var}(Y_i) = 4\tau_1(1 + (1 - \tau_1)(1 - \log\tau_1 + \log\tau_2)^2)$.

Now consider the other covariance terms. Write $P_i = 1 - F(Z_i)$, where $F(x) = \Phi(x)$ for one-sided P_i's or $F(x) = F_{\chi_1^2}(x^2)$ for two-sided P_i's. Define $G(Z_i|\tau_2) = -2\log\left(\frac{1-F(Z_i)}{\tau_2}\right)$, then $Y_i = G(Z_i|\tau_2) I(Z_i > F^{-1}(1 - \tau_1))$. Using Mehler's formula (Patel and Read, 1996),

$$\begin{aligned} E(Y_i Y_j) &= \sum_{k=0}^{\infty} \frac{\rho_{ij}^k}{k!} \int_{F^{-1}(1-\tau_1)}^{\infty} \int_{F^{-1}(1-\tau_1)}^{\infty} \phi(x)\phi(y) G(x|\tau_2) G(y|\tau_2) H_k(x) H_k(y) dx dy \\ &= \sum_{k=0}^{\infty} \frac{\rho_{ij}^k}{k!} I_i(k) I_j(k). \end{aligned}$$

where ρ_{ij} is the correlation coefficient of Z_i and Z_j, and $I_j(k) = \int_{F^{-1}(1-\tau_1)}^{\infty} \phi(x) G(x|\tau_2) H_k(x) dx$, $H_k(x)$ is the k-th order Hermite polynomial. Since $H_0(x) \equiv 0$, $E(Y_i) = I_i(0)$. Thus,

$$Cov(Y_i, Y_j) = E(Y_i Y_j) - E(Y_i)E(Y_j) = \sum_{k=1}^{\infty} \frac{\rho_{ij}^k}{k!} I_i(k) I_j(k).$$

1.2 The p-value of oTFisher_minp in (9)

Consider a set of truncation parameters $\{(\tau_{1k}, \tau_{2k}), k = 1, ..., K\}$. For simplicity, denote $T_k = T_n(\tau_{1k}, \tau_{2k})$ and t_k is the observed value of T_k. Let $G_k(t) = \mathbb{P}(T_k > t)$ be the survival function of

T_k. $G_k(t_k)$ is the observed p-value of T_k. Denote the oTFisher_minp statistic as $minP = \min_k G_k(T_k)$. For any constant $p_0 \in (0, 1)$, we have

$$P(minP > p_0) = P(\min_k G_k(T_k) > p_0) = P(T_k < G_k^{-1}(p_0), k = 1, ..., K).$$

Denote $Y_{ik} = -2\log\left(\frac{P_i}{\tau_{2k}}\right) I(P_i < \tau_{1k})$, $i = 1, ..., n$. We have $T_k = \sum_{i=1}^n Y_{ik}$, $k = 1, ..., K$. By the CLT, the vector $\mathbf{T} = (T_1, ..., T_K)'$ is asymptotically multivariate normal as $n \to \infty$. Specifically, if we denote $\boldsymbol{\mu}_T = (\mu_{T_1}, ..., \mu_{T_K})'$, we have

$$\boldsymbol{\Omega}^{-1/2}(\mathbf{T} - \boldsymbol{\mu}_T) \xrightarrow{D} N(\mathbf{0}, \mathbf{I}_{K\times K}).$$

This distribution convergence means that $Z_k \equiv \frac{T_k - \mu_{T_k}}{\sigma_{T_k}} \xrightarrow{D} N(0, 1)$, $\frac{G_k^{-1}(p_0) - \mu_{T_k}}{\sigma_{T_k}} \to \bar{\Phi}^{-1}(p_0)$, and $\mathbf{R}^{-1/2} Z \xrightarrow{D} N(\mathbf{0}, \mathbf{I}_{K\times K})$, where $\mathbf{R}$ is the correlation matrix obtained by scaling the covariance matrix $\boldsymbol{\Omega}$ of $\mathbf{T} = (T_1, ..., T_K)'$. That is, $\mathbf{R} = \boldsymbol{\Lambda}\boldsymbol{\Omega}\boldsymbol{\Lambda}$ with the diagonal matrix $\boldsymbol{\Lambda} = \text{diag}(1/\sqrt{\boldsymbol{\Omega}_{kk}})_{1\le k\le K} = \text{diag}(1/\sigma_{T_k})_{1\le k\le K}$. Therefore,

$$P(T_k < G_k^{-1}(p_0), k = 1, ..., K) = (1 + o(1))\Phi_{\mathbf{R}}(\bar{\Phi}^{-1}(p_0), ..., \bar{\Phi}^{-1}(p_0)),$$

where $\Phi_{\mathbf{R}}$ denotes the CDF of a multivariate Gaussian distribution with mean zero and correlation matrix R. Equivalently, the p-value of oTFisher_minp at the observed statistic p_0 is

$$\mathbb{P}(minP < p_0) = (1 + o(1))[1 - \Phi_{\mathbf{R}}(\bar{\Phi}^{-1}(p_0), ..., \bar{\Phi}^{-1}(p_0))]. \tag{S2}$$

1.3 Lemma 2

LEMMA 2. *Following the same notations in Lemma 1, for* $1 \le l, k \le K$,

$$\Omega_{lk} = \text{Cov}[T_n(\tau_{1l}, \tau_{2l}), T_n(\tau_{1k}, \tau_{2k})] = \sum_{1\le i,j\le n} \sum_{r=1}^{\infty} \frac{\rho_{ij}^r}{r!} I_{il}(r) I_{jk}(r),$$

where $I_{jk}(r) = \int_{F^{-1}(1-\tau_{1k})}^{\infty} \phi(x) G(x|\tau_{2k}) H_r(x) dx$.

PROOF OF LEMMA 2. Given any two TFisher statistics T_l and T_k,

$$\Omega_{lk} = \text{Cov}(T_l, T_k) = \sum_{1\le i,j\le n} \text{Cov}(Y_{il}, Y_{jk}).$$

When $i = j$ and $l = k$, we have $\text{Var}(Y_{il}) = 4\tau_{1l}\left(1 + (1 - \tau_{1l})(1 - \log\tau_{1l} + \log\tau_{2l})^2\right)$. In general, we apply Mehler's formula (Patel and Read, 1996) to get

$$Cov(Y_{il}, Y_{jk}) = \sum_{r=1}^{\infty} \frac{\rho_{ij}^r}{r!} I_{il}(r) I_{jk}(r),$$

where $I_{jk}(r) = \int_{F^{-1}(1-\tau_{1k})}^{\infty} \phi(x)G(x|\tau_{2k})H_r(x)dx,\ j = 1, ..., n$ and $k = 1, ..., K$. $G(x|\tau_{2k}) = -2\log\left(\frac{1-F(x)}{\tau_{2k}}\right)$ and $H_r(x)$ is the r-th order Hermite polynomial.

2 SIMULATION STUDIES

2.1 Calculation accuracy

Table S1. Type I error control for SNP-set testing by the oTFisher procedures under quantitative and binary traits. SNP-set size $n = 10$, sample size $N = 1000$. The search domain $\tau_1 = \tau_2 \in \{0.00001, 0.001, 0.005, 0.01, 0.05, 0.1, 0.2, 0.5, 0.7, 1\}$.

	rare variants				common variants			
	oTFisher_cct		oTFisher_minp		oTFisher_cct		oTFisher_minp	
α	GB	Hybrid	GB	Hybrid	GB	Hybrid	GB	hybrid
Continuous trait								
0.1	0.62	0.62	0.49	0.49	0.62	0.61	0.50	0.5
0.05	0.72	0.72	0.49	0.49	0.72	0.72	0.51	0.49
0.01	0.90	0.89	0.52	0.47	0.97	0.86	0.60	0.45
0.005	0.93	0.89	0.54	0.46	1.06	0.85	0.63	0.43
0.001	1.04	0.89	0.63	0.46	1.22	0.85	0.72	0.42
0.0005	1.12	0.95	0.72	0.51	1.22	0.91	0.64	0.46
0.0001	1.55	1.11	1.03	0.58	1.20	1	0.80	0.55
0.00005	1.79	1.22	1.21	0.73	1.60	1.08	1.40	0.67
0.00001	2.58	1.9	1.99	1.45	3.00	1.63	3.00	1.36
Binary trait								
0.1	0.63	0.63	0.49	0.49	0.60	0.6	0.49	0.49
0.05	0.72	0.72	0.49	0.48	0.70	0.7	0.48	0.48
0.01	0.90	0.9	0.49	0.46	0.91	0.84	0.55	0.43
0.005	0.92	0.88	0.50	0.45	0.97	0.83	0.59	0.42
0.001	0.93	0.85	0.51	0.41	1.16	0.73	0.75	0.37
0.0005	0.92	0.85	0.64	0.43	1.29	0.8	0.87	0.39
0.0001	1.60	0.89	0.80	0.47	1.83	0.71	1.32	0.37
0.00005	1.20	0.94	1.20	0.51	2.12	0.76	1.58	0.51
0.00001	3.00	1.04	3.00	0.8	3.02	0.95	2.81	1.11

Table S2. Type I error control for SNP-set testing by the oTFisher procedures under quantitative and binary traits. SNP-set size $n = 10$, sample size $N = 1000$. The search domain $\tau_1 = \tau_2 \in \{0.001, 0.005, 0.01, 0.05, 0.1, 0.2, 0.5, 0.7, 1\}$.

	rare variants				common variants			
	oTFisher_cct		oTFisher_minp		oTFisher_cct		oTFisher_minp	
α	GB	Hybrid	GB	Hybrid	GB	Hybrid	GB	hybrid
Continuous trait								
0.1	0.71	0.71	0.62	0.62	0.69	0.69	0.62	0.63
0.05	0.82	0.82	0.61	0.61	0.81	0.81	0.62	0.62
0.01	0.99	0.98	0.64	0.59	1.04	0.96	0.7	0.57
0.005	1.02	0.99	0.66	0.57	1.12	0.95	0.76	0.55
0.001	1.11	0.97	0.75	0.56	1.38	0.92	1.00	0.52
0.0005	1.17	0.97	0.84	0.58	1.54	0.94	1.17	0.55
0.0001	1.44	1.08	1.23	0.75	2.12	1.11	1.91	0.73
0.00005	1.63	1.06	1.54	0.80	2.47	1.19	2.41	0.86
0.00001	2.29	1.12	2.29	1.11	3.80	1.43	4.20	1.24
Binary trait								
0.1	0.71	0.72	0.63	0.63	0.69	0.69	0.62	0.62
0.05	0.81	0.82	0.60	0.60	0.80	0.80	0.61	0.61
0.01	0.99	0.99	0.63	0.59	1.01	0.94	0.67	0.55
0.005	1.00	0.98	0.64	0.57	1.07	0.92	0.72	0.52
0.001	1.07	0.94	0.71	0.52	1.29	0.86	0.92	0.47
0.0005	1.11	0.92	0.78	0.53	1.44	0.85	1.08	0.48
0.0001	1.29	0.94	1.10	0.65	1.89	0.93	1.69	0.61
0.00005	1.46	1.01	1.33	0.75	2.15	0.98	2.05	0.72
0.00001	1.95	1.10	2.06	1.00	2.98	1.12	3.35	0.95

Table S3. Type I error control for SNP-set testing by the oTFisher procedures under quantitative traits. SNP-set size $n = 10$, sample size $N = 1000$. The search domain $\tau_1 = \tau_2 \in \{0.01, 0.05, 0.1, 0.2, 0.5, 0.7, 1\}$.

	rare variants				common variants			
	oTFisher_cct		oTFisher_minp		oTFisher_cct		oTFisher_minp	
α	GB	Hybrid	GB	Hybrid	GB	Hybrid	GB	hybrid
Continuous trait								
0.1	0.94	0.94	0.78	0.79	0.9	0.91	0.78	0.79
0.05	0.99	0.99	0.77	0.77	0.97	0.98	0.77	0.77
0.01	1.01	0.98	0.78	0.69	1.07	0.95	0.81	0.62
0.005	1.03	0.97	0.8	0.66	1.14	0.92	0.87	0.58
0.001	1.12	0.98	0.96	0.68	1.39	0.93	1.17	0.58
0.0005	1.2	0.97	1.08	0.69	1.55	0.96	1.4	0.63
0.0001	1.45	1.01	1.6	0.86	2.11	1	2.29	0.77
0.00005	1.6	1.02	1.88	0.97	2.49	1.07	2.84	0.86
0.00001	2.25	1.2	3.06	1.22	3.87	1.29	4.97	1.19

Table S4. Type I error control for SNP-set testing by the oTFisher procedures under quantitative and binary traits by GB and hybrid methods. 5×10^5 simulations; genotype data were randomly generated in each simulation. Set size $n = 100$; sample size $N = 1000$. Search domain $\tau_1 = \tau_2 \in \{0.01, 0.05, 0.1, 0.2, 0.5, 0.7, 1\}$.

	rare variants				common variants			
	oTFisher_cct		oTFisher_minp		oTFisher_cct		oTFisher_minp	
α	GB	Hybrid	GB	Hybrid	GB	Hybrid	GB	hybrid
Continuous trait								
0.1	1.00	1.02	0.87	0.89	0.96	0.96	0.79	0.79
0.05	1.04	1.06	0.88	0.89	1.00	1.00	0.81	0.81
0.01	1.19	0.98	1.02	0.76	1.23	0.94	1.03	0.66
0.005	1.26	0.98	1.12	0.75	1.37	0.93	1.17	0.63
0.001	1.73	1.01	1.68	0.84	2.11	0.96	1.95	0.70
0.0005	2.07	1.07	2.09	1.00	2.47	0.93	2.55	0.78
0.0001	3.00	1.32	3.70	1.42	3.73	1.14	4.47	1.14
Binary trait								
0.1	1.02	1.01	0.88	0.88	0.97	0.97	0.80	0.79
0.05	1.03	1.04	0.88	0.88	1.01	1.00	0.81	0.80
0.01	1.18	0.96	1.01	0.73	1.20	0.94	0.97	0.65
0.005	1.25	0.95	1.12	0.73	1.33	0.92	1.10	0.63
0.001	1.72	1.02	1.63	0.87	1.83	1.00	1.79	0.74
0.0005	1.97	1.04	2.15	0.97	2.22	1.04	2.22	0.85
0.0001	3.20	1.24	3.87	1.42	3.60	0.86	4.33	0.78

2.2 Statistical power

The algorithm to get the empirical p-values of the RTP and the ARTP is consistent with (Yu et al., 2009) except that we directly simulate the Z-scores instead of permuting the genotype data for faster computation. Specifically, we simulate in total M Z-score vectors $Z^s = (Z_1^s, ..., Z_n^s) \sim N(0, \Sigma)$, $s = 1, ..., M$, where Σ is the estimated correlation matrix of the Z-scores of the SNP-set from data. By (5), we obtain SNP p-values and order them $P_{(1)}^s \leq, ..., \leq P_{(n)}^s$. For simplicity, we use $s = 0$ to index the observed quantities from the data instead of the simulation. At each given rank-truncation point k_j, $j = 1, ..., J$, we get the RTP statistics $T^s(k_j) = \sum_{i=1}^{k_j} -2\log(P_{(j)}^s)$, and its empirical p-value $P^s(k_j) = \frac{\sum_{l=0}^{M} I(T^l(k_j) \geq T^s(k_j))}{M+1}$, $s = 0, 1, ..., M$. Thus, the ARTP statistics are $minP^s = \min_{1 \leq j \leq J} P^s(k_j)$, and the empirical p-values of the ARTP are $\frac{\sum_{l=0}^{M} I(minP^l \leq minP^0)}{M+1}$, $s = 0, 1, ..., M$. In this paper we used $M = 40,000$ simulations.

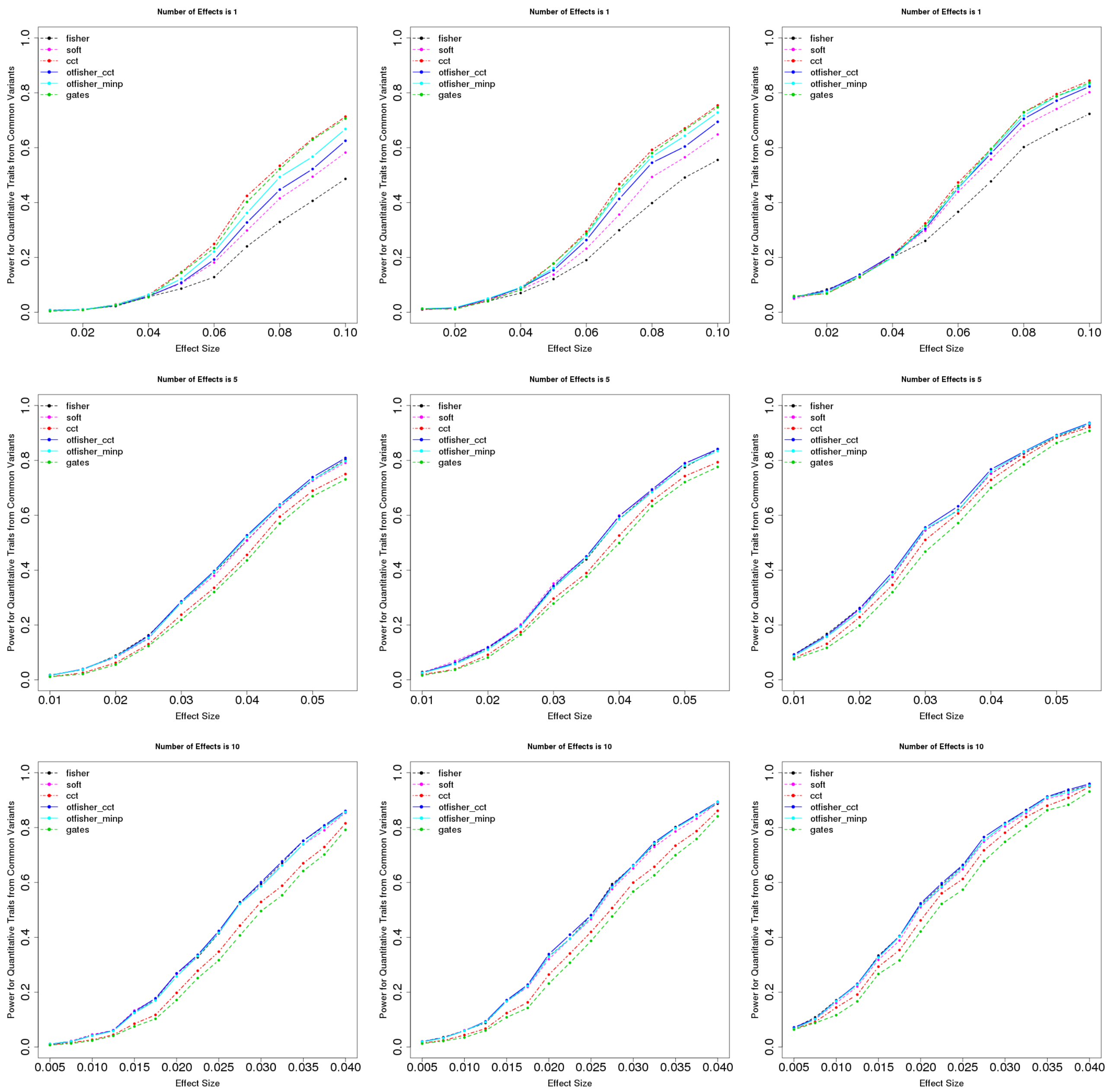

Figure S1: Statistical power for quantitative traits based on common variants. Vary the effect size at a given number of causal SNPs $m = 1$, 5, or 10, corresponding to the three rows. Left, middle, right columns: $\alpha = 0.005$, 0.01, and 0.05, respectively. Setting: $n = 100$, $N = 10000$, and τ-searching domain $\mathcal{T} = \{0.001, 0.005, 0.01, 0.05, 0.1, 0.2, 0.5, 0.7, 1\}$ for row 1 and $\mathcal{T} = \{0.01, 0.05, 0.1, 0.2, 0.5, 0.7, 1\}$ for row 2 and 3.

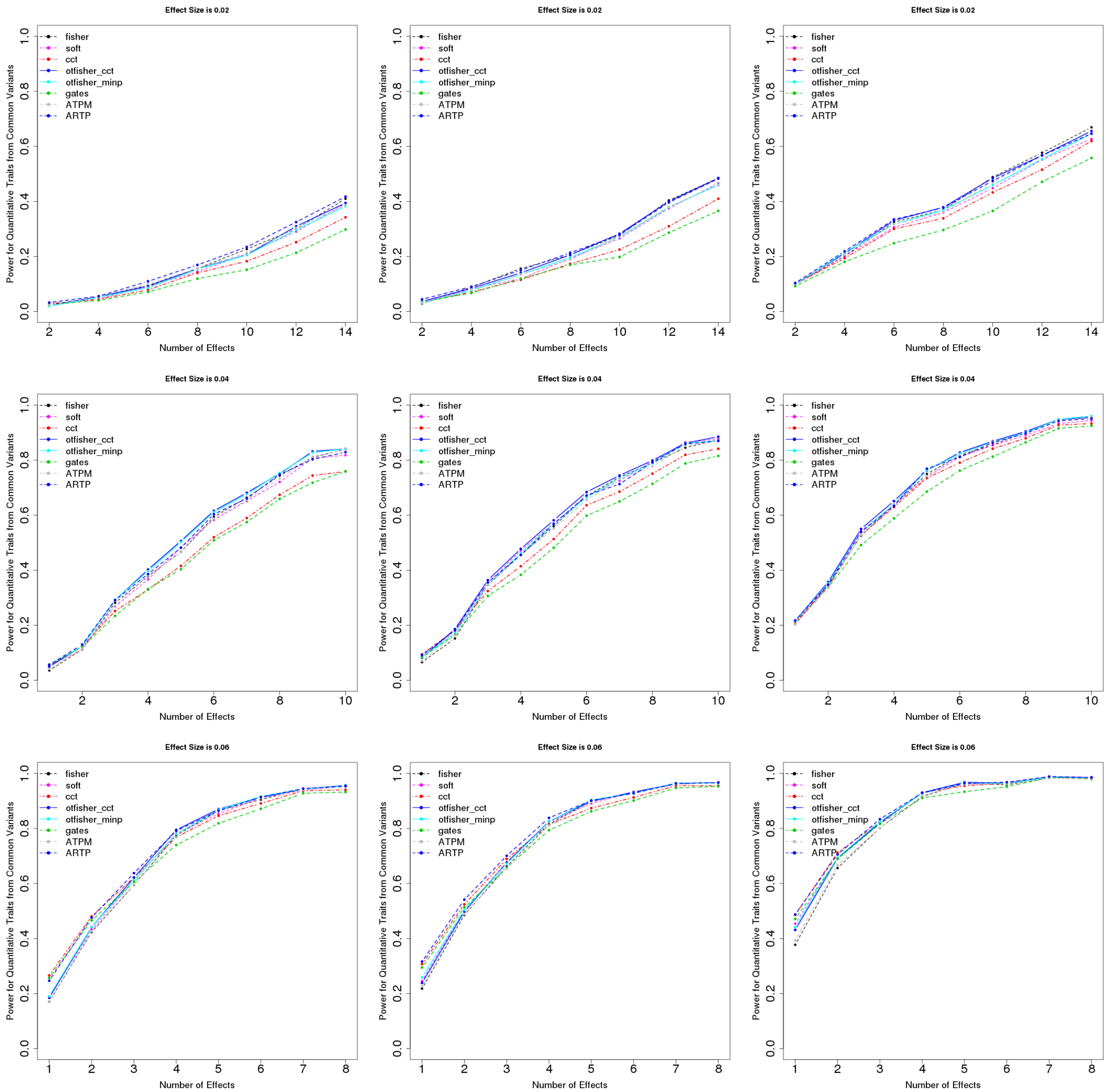

Figure S2: Statistical power for quantitative traits based on common variants. Vary the number of causal SNPs at a given effect size (nonzero β) being 0.02, 0.04, and 0.06, corresponding to the three rows. Left, middle, right columns: $\alpha = 0.005$, 0.01, and 0.05, respectively. Setting: $n = 100$, $N = 10000$, and τ-searching domain $\mathcal{T} = \{0.01, 0.05, 0.1, 0.2, 0.5, 0.7, 1\}$.

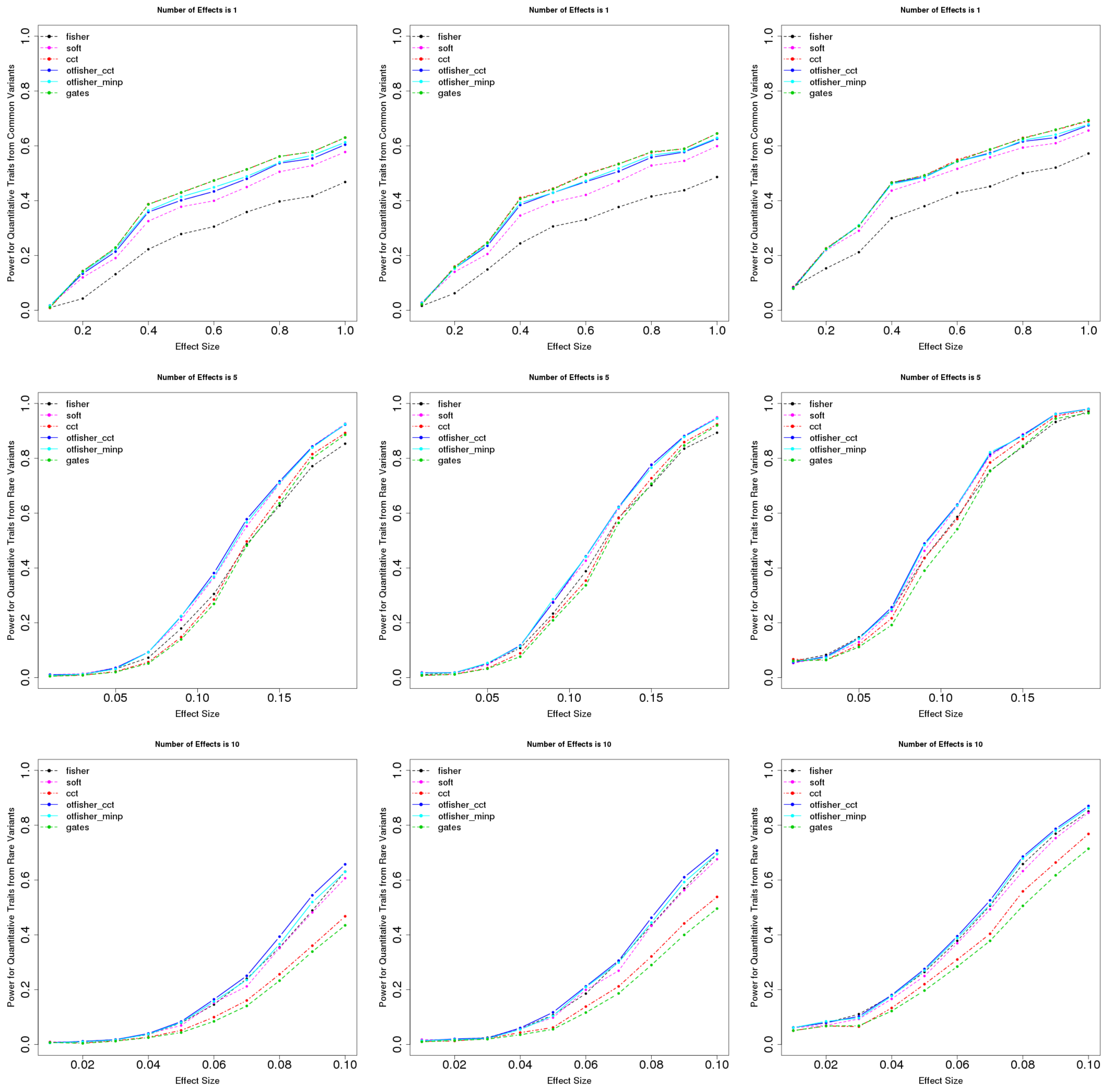

Figure S3: Statistical power for quantitative traits based on rare variants. Vary the effect size at a given number of causal SNPs $m = 1$, 5, or 10, corresponding to the three rows. Left, middle, right columns: $\alpha = 0.005$, 0.01, and 0.05, respectively. Setting: $n = 100$, $N = 10000$, and τ-searching domain $\mathcal{T} = \{0.001, 0.005, 0.01, 0.05, 0.1, 0.2, 0.5, 0.7, 1\}$ for row 1 and τ-searching domain $\mathcal{T} = \{0.01, 0.05, 0.1, 0.2, 0.5, 0.7, 1\}$ for row 2 and 3.

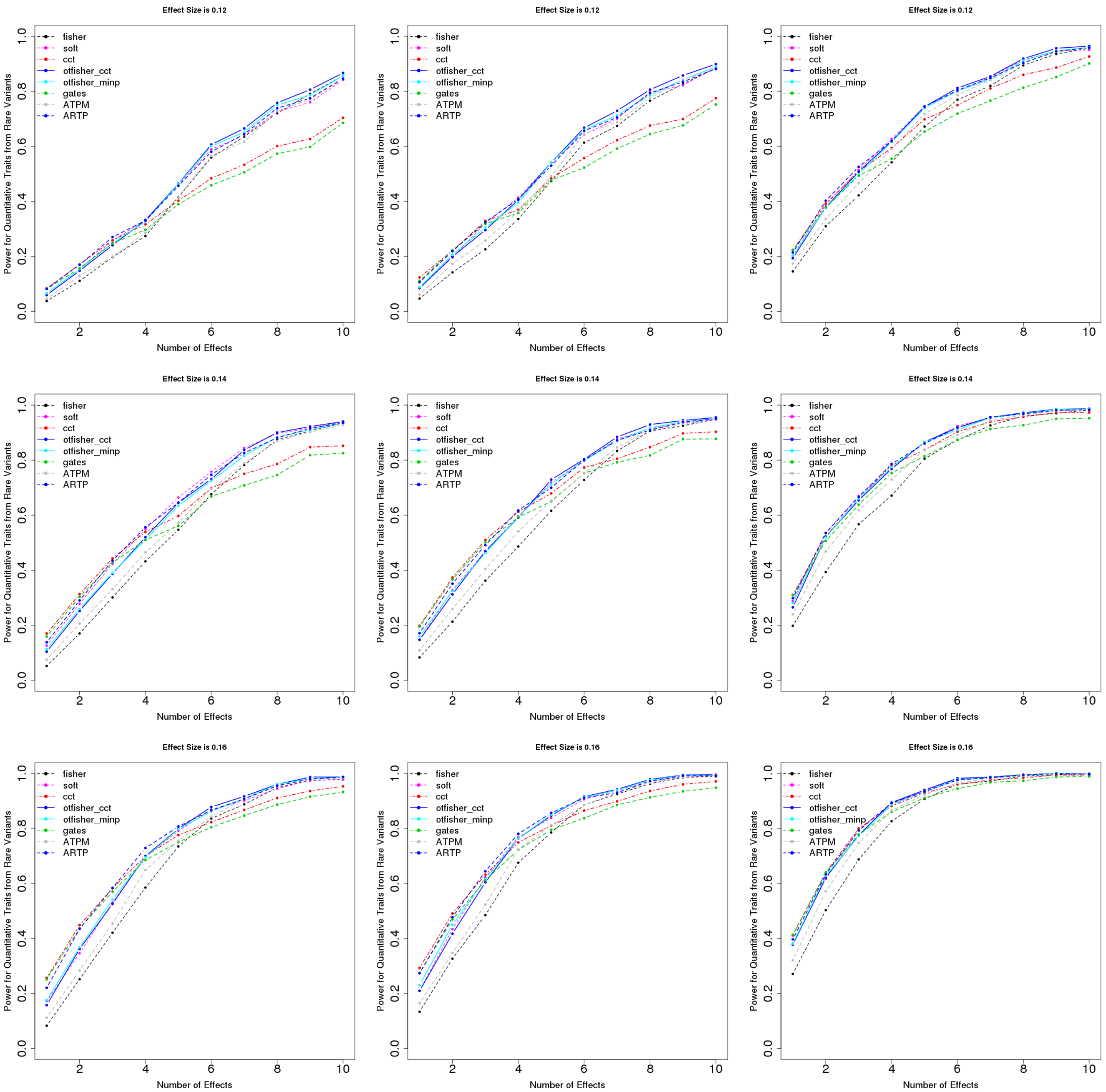

Figure S4: Statistical power for quantitative traits based on rare variants. Vary the number of causal SNPs at a given effect size (nonzero β) being 0.12, 0.14, and 0.16, corresponding to the three rows. Left, middle, right columns: $\alpha = 0.005$, 0.01, and 0.05, respectively. Setting: $n = 100$, $N = 10000$, and τ-searching domain $\mathcal{T} = \{0.01, 0.05, 0.1, 0.2, 0.5, 0.7, 1\}$.

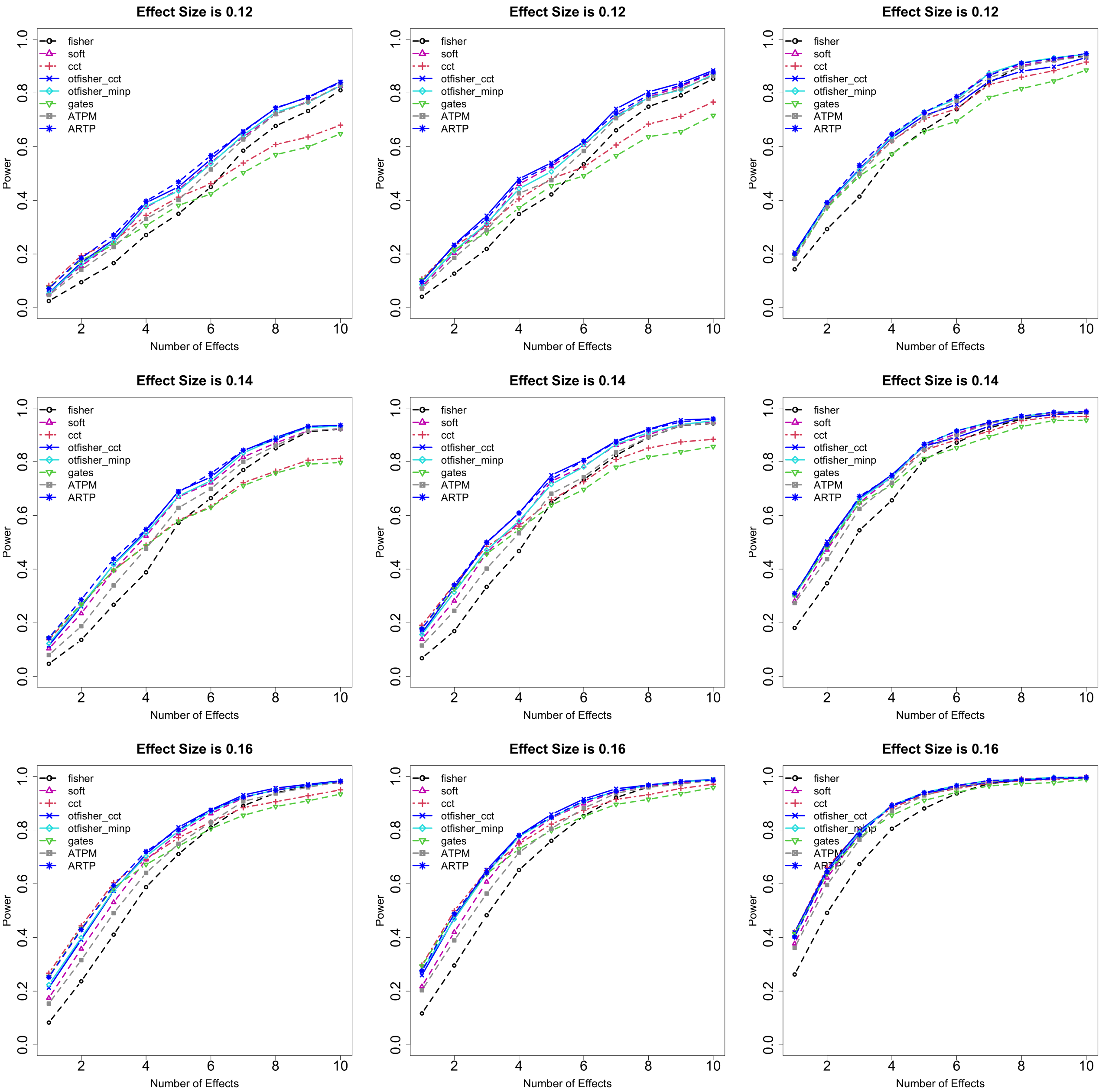

Figure S5: Statistical power for quantitative traits based on rare variants. Vary the number of causal SNPs at a given effect size (nonzero β) being 0.12, 0.14, and 0.16, corresponding to the three rows. Left, middle, right columns: $\alpha = 0.005$, 0.01, and 0.05, respectively. Setting: $n = 100$, $N = 10000$, and τ-searching domain $\mathcal{T} = \{0.001, 0.005, 0.01, 0.05, 0.1, 0.2, 0.5, 0.7, 1\}$.

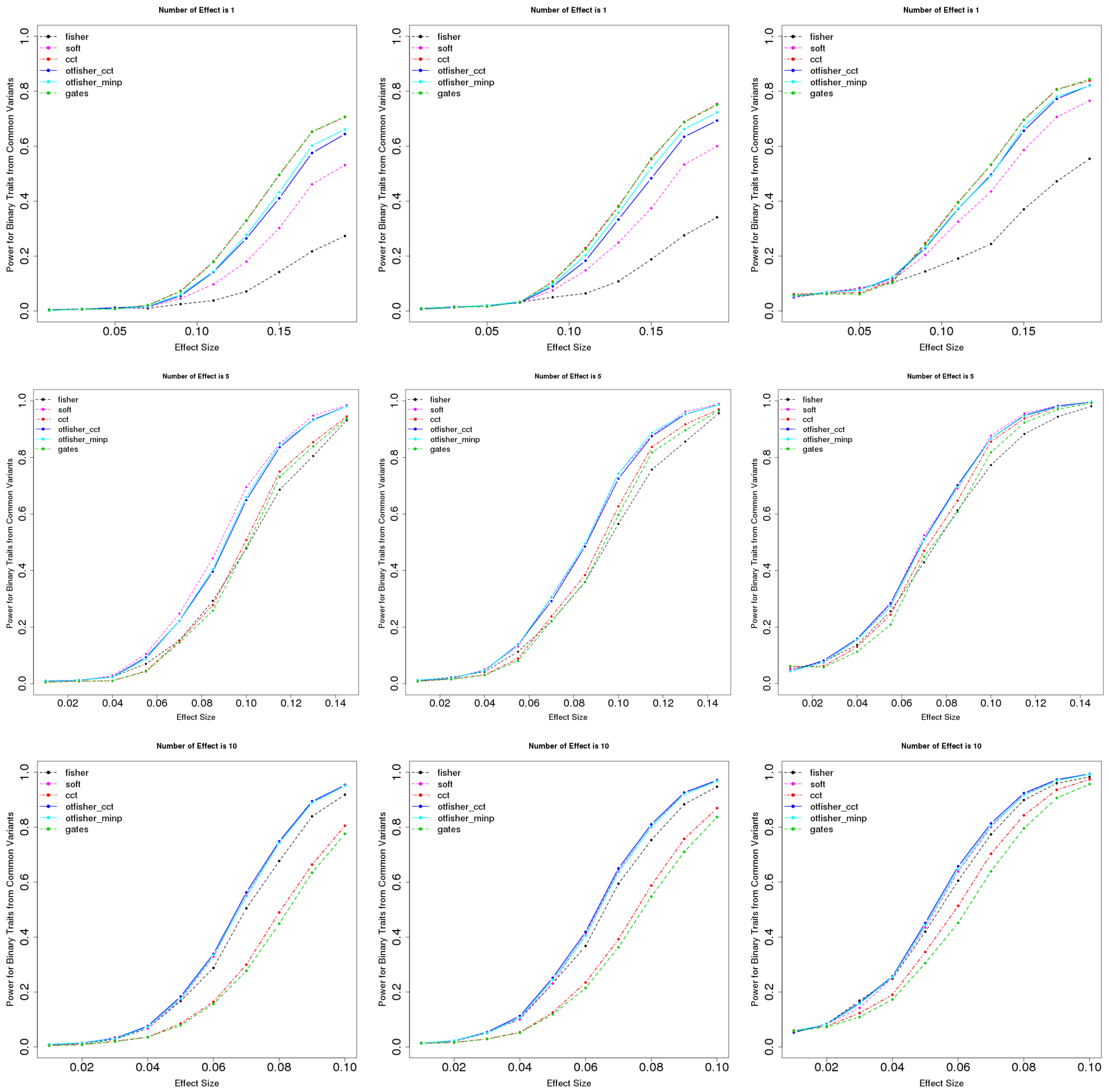

Figure S6: Statistical power for binary traits based on common variants. Vary the effect size at a given number of causal SNPs $m = 1$, 5, or 10, corresponding to the three rows. Left, middle, right columns: $\alpha = 0.005$, 0.01, and 0.05, respectively. Setting: $n = 100$, $N = 10000$, and τ-searching domain $\mathcal{T} = \{0.01, 0.05, 0.1, 0.2, 0.5, 0.7, 1\}$.

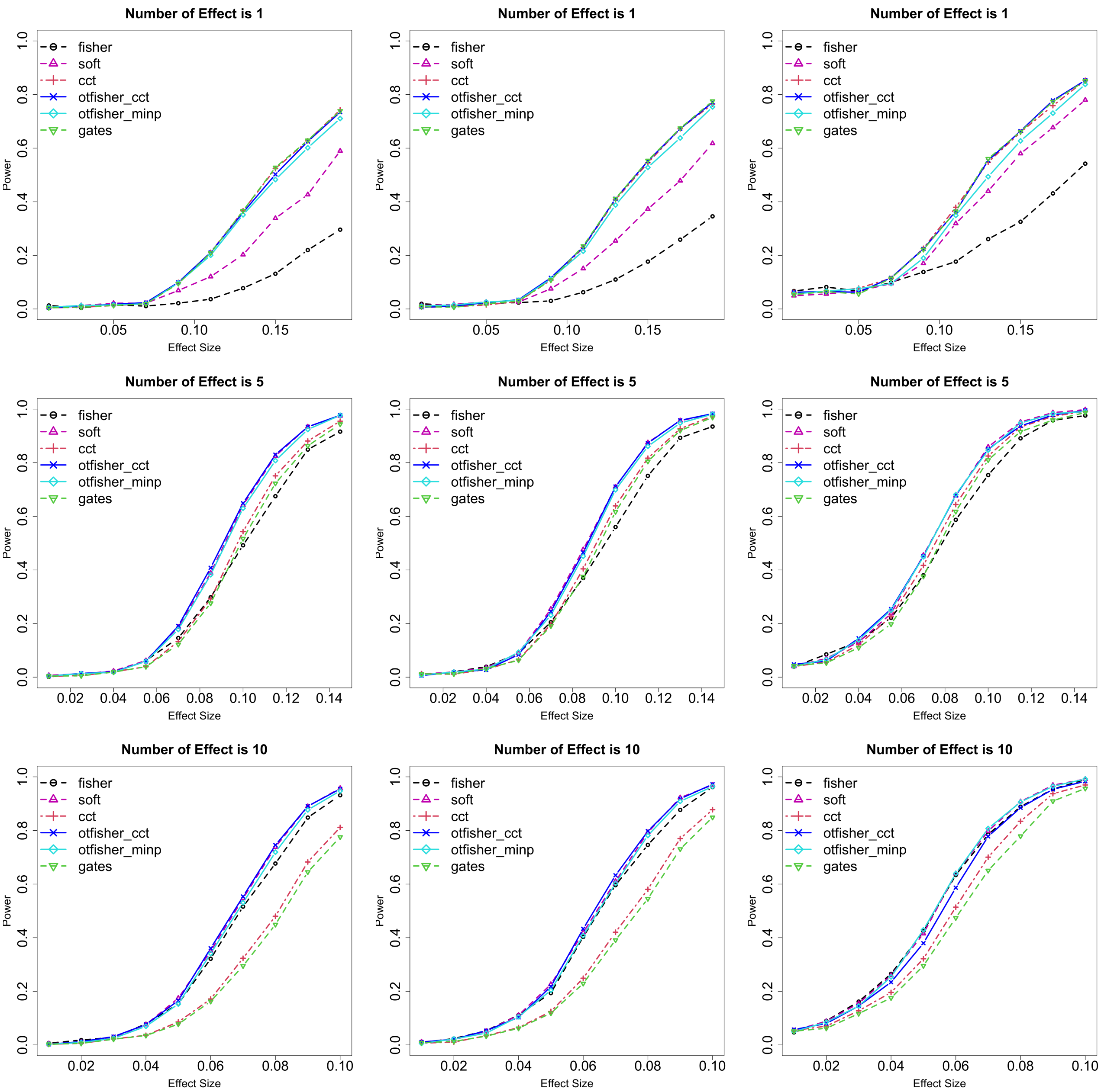

Figure S7: Statistical power for binary traits based on common variants. Vary the effect size at a given number of causal SNPs $m = 1$, 5, or 10, corresponding to the three rows. Left, middle, right columns: $\alpha = 0.005$, 0.01, and 0.05, respectively. Setting: $n = 100$, $N = 10000$, $\mathcal{T} = \{0.0001, 0.0005, 0.001, 0.005, 0.01, 0.05, 0.1, 0.2, 0.5, 0.7, 1\}$ for row 1, and $\mathcal{T} = \{0.001, 0.005, 0.01, 0.05, 0.1, 0.2, 0.5, 0.7, 1\}$ for rows 2 and 3.

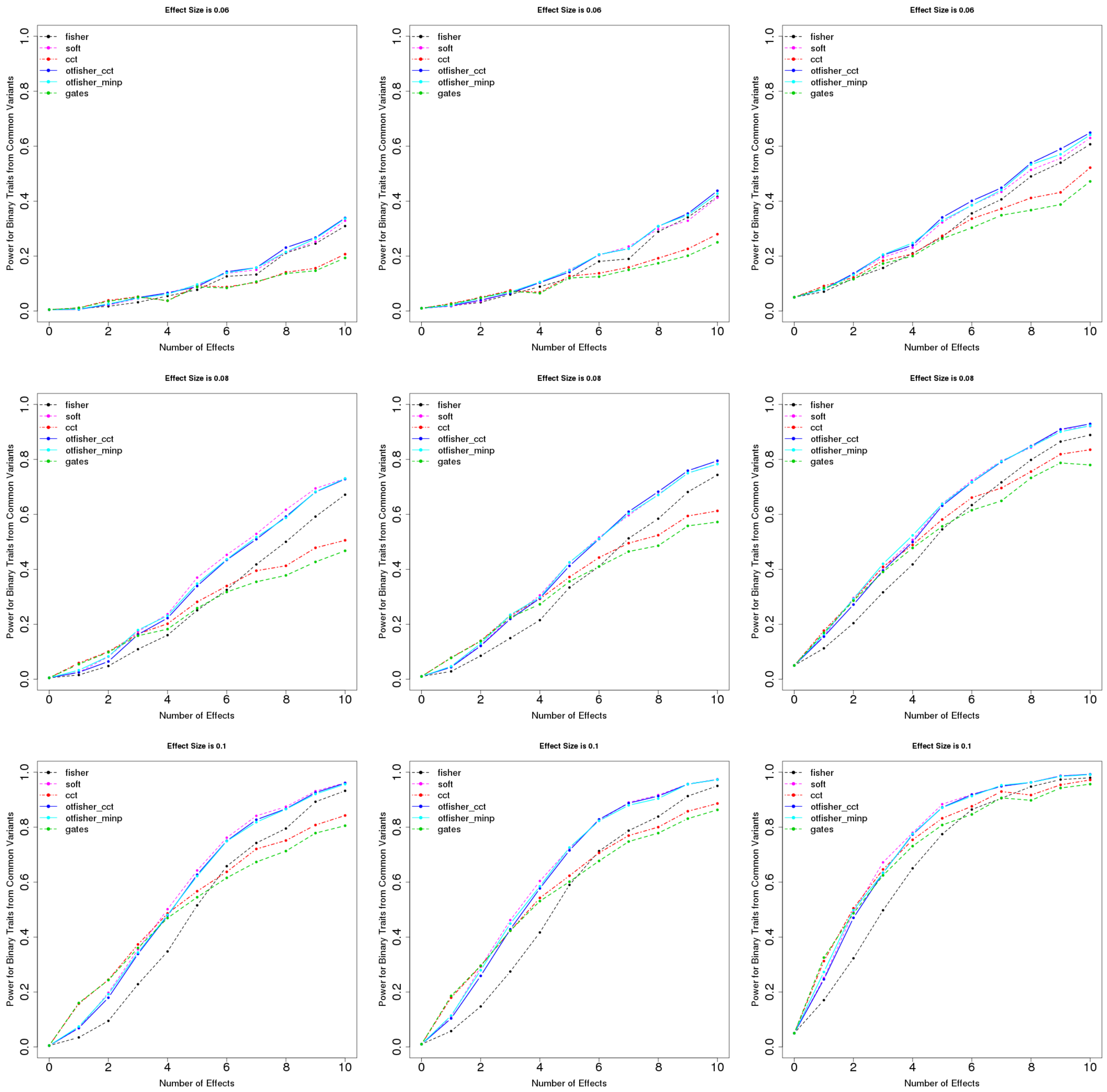

Figure S8: Statistical power for binary traits based on common variants. Vary the number of causal SNPs at a given effect size (nonzero β) being 0.1, 0.11, and 0.12, corresponding to the three rows. Left, middle, right columns: $\alpha = 0.005$, 0.01, and 0.05, respectively. Setting: $n = 100$, $N = 10000$, and τ-searching domain $\mathcal{T} = \{0.01, 0.05, 0.1, 0.2, 0.5, 0.7, 1\}$.

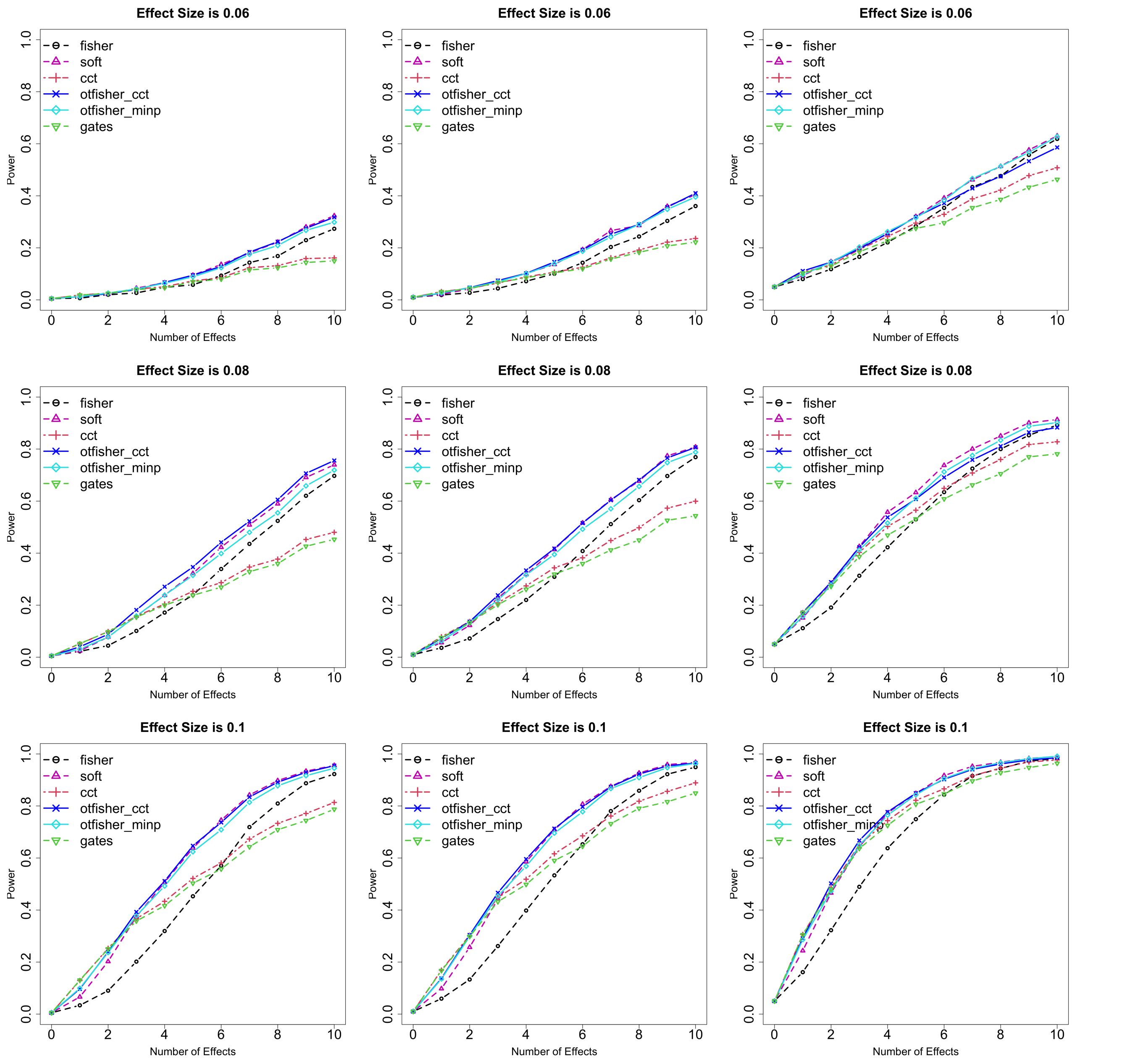

Figure S9: Statistical power for binary traits based on common variants. Vary the number of causal SNPs at a given effect size (nonzero β) being 0.1, 0.11, and 0.12, corresponding to the three rows. Left, middle, right columns: $\alpha = 0.005$, 0.01, and 0.05, respectively. Setting: $n = 100$, $N = 10000$, and τ-searching domain $\mathcal{T} = \{0.001, 0.005, 0.01, 0.05, 0.1, 0.2, 0.5, 0.7, 1\}$.

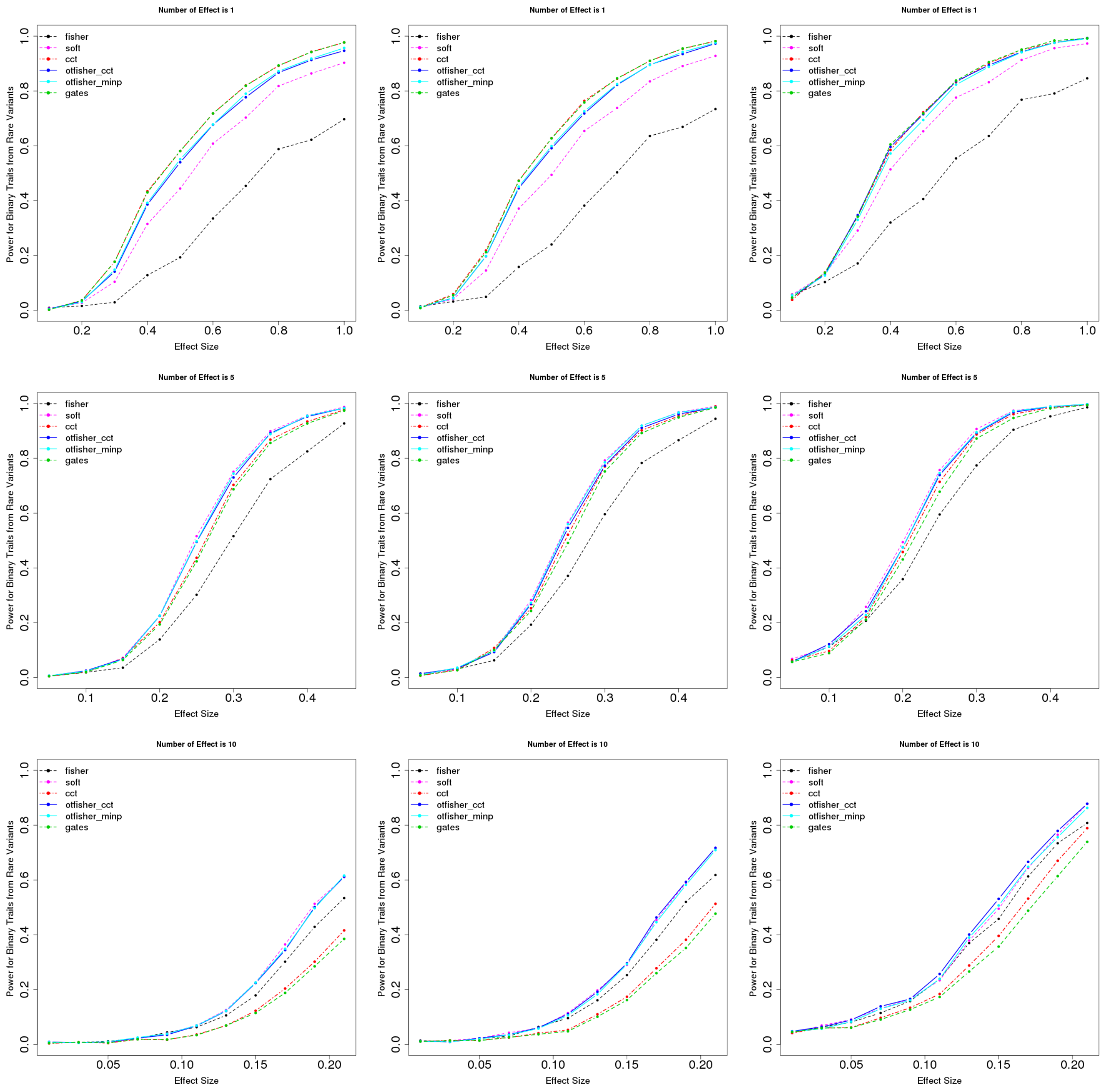

Figure S10: Statistical power for binary traits based on rare variants. Vary the effect size at a given number of causal SNPs $m = 1$, 5, or 10, corresponding to the three rows. Left, middle, right columns: $\alpha = 0.005$, 0.01, and 0.05, respectively. Setting: $n = 100$, $N = 10000$, and $\mathcal{T} = \{0.001, 0.005, 0.01, 0.05, 0.1, 0.2, 0.5, 0.7, 1\}$ for row 1 $\mathcal{T} = \{0.01, 0.05, 0.1, 0.2, 0.5, 0.7, 1\}$ for row 2 and 3.

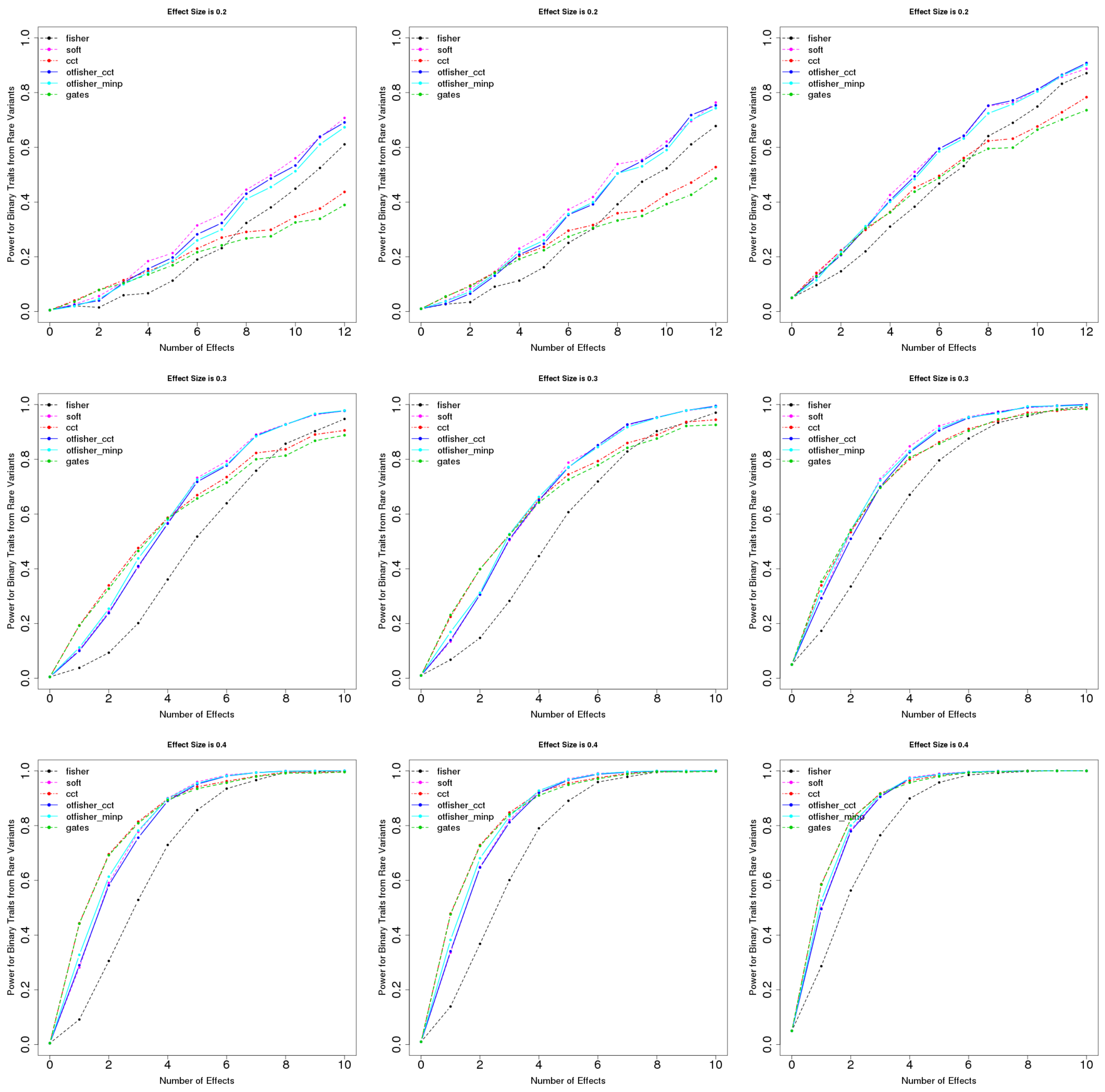

Figure S11: Statistical power for binary traits based on rare variants. Vary the number of causal SNPs at a given effect size (nonzero β) being 0.4, 0.5, and 0.6, corresponding to the three rows. Left, middle, right columns: $\alpha = 0.005$, 0.01, and 0.05, respectively. Setting: $n = 100$, $N = 10000$, and τ-searching domain $\mathcal{T} = \{0.01, 0.05, 0.1, 0.2, 0.5, 0.7, 1\}$.

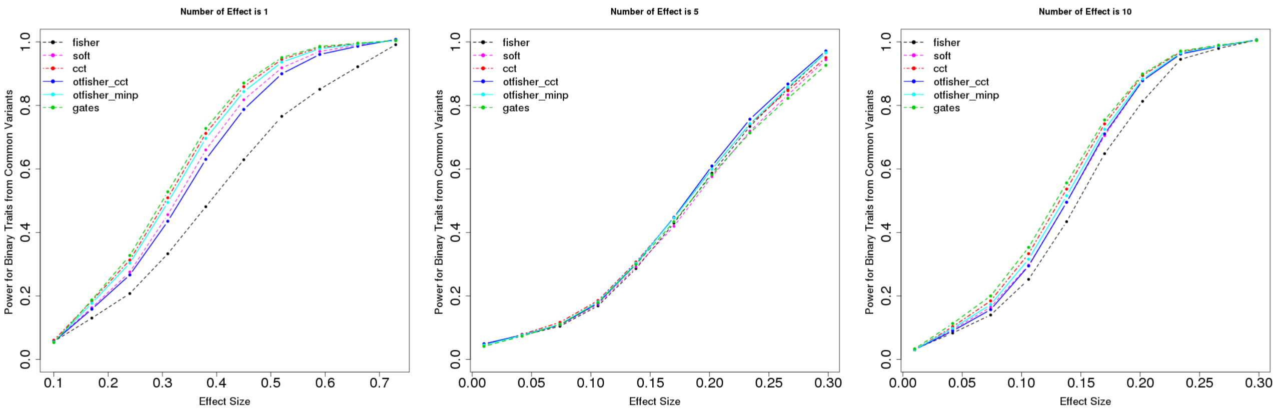

Figure S12: Statistical power for binary traits based on common variants. Vary the effect size at given number of causal SNPs $m = 1$, 5, or 10, corresponding to the left, middle, and right figures. $\alpha = 0.05$. Setting: $n = 25$, $N = 10000$, and $\mathcal{T} = \{0.01, 0.05, 0.1, 0.2, 0.5, 0.7, 1\}$.

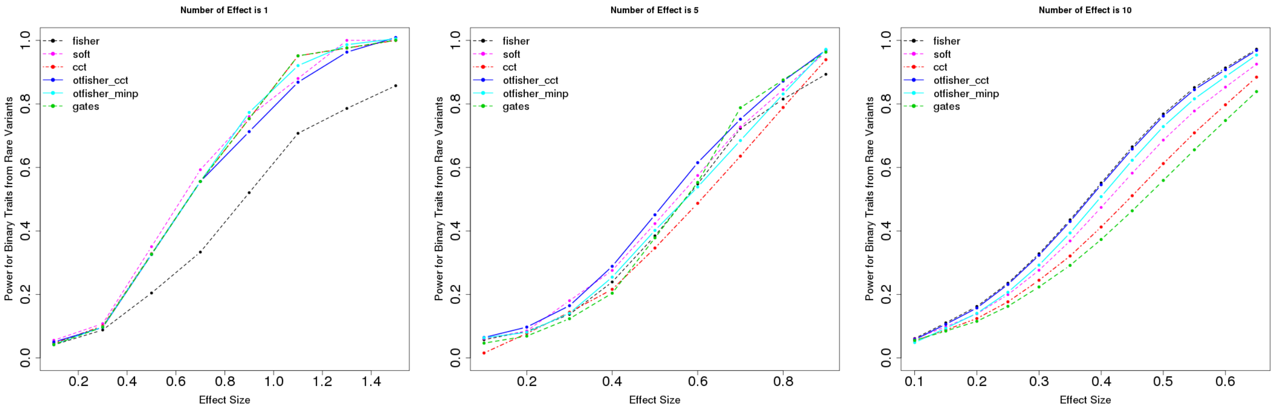

Figure S13: Statistical power for binary traits based on rare variants. Vary the effect size at given number of causal SNPs $m = 1$, 5, or 10, corresponding to the left, middle, and right figures. $\alpha = 0.05$. Setting: $n = 25$, $N = 10000$, and $\mathcal{T} = \{0.01, 0.05, 0.1, 0.2, 0.5, 0.7, 1\}$.

2.3 SNP screening

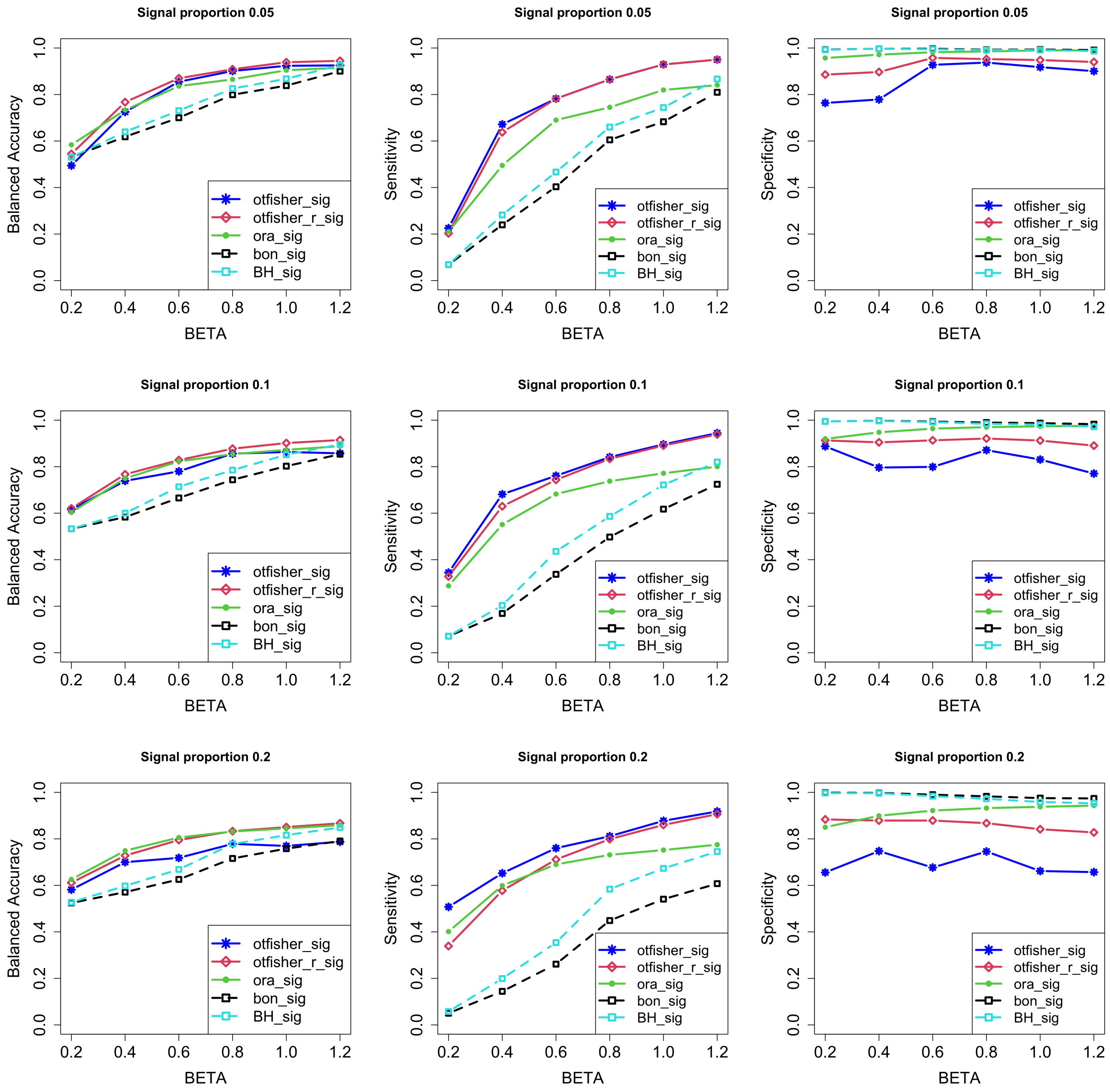

Figure S14: Accuracy at the SNP-screening stage under the continuous trait model. The proportion of causal SNPs in the two causal genes (containing 200 SNPs) = 0.05 (row 1), 0.1 (row 2), and 0.2 (row 3). Accuracy measures: balanced accuracy (column 1), sensitivity (column 2), and specificity (column 3). The search domain in oTFisher is $\tau_1 = \tau_2 \in \{0.01, 0.05, 0.1, 0.2, 0.5, 0.7, 1\}$.

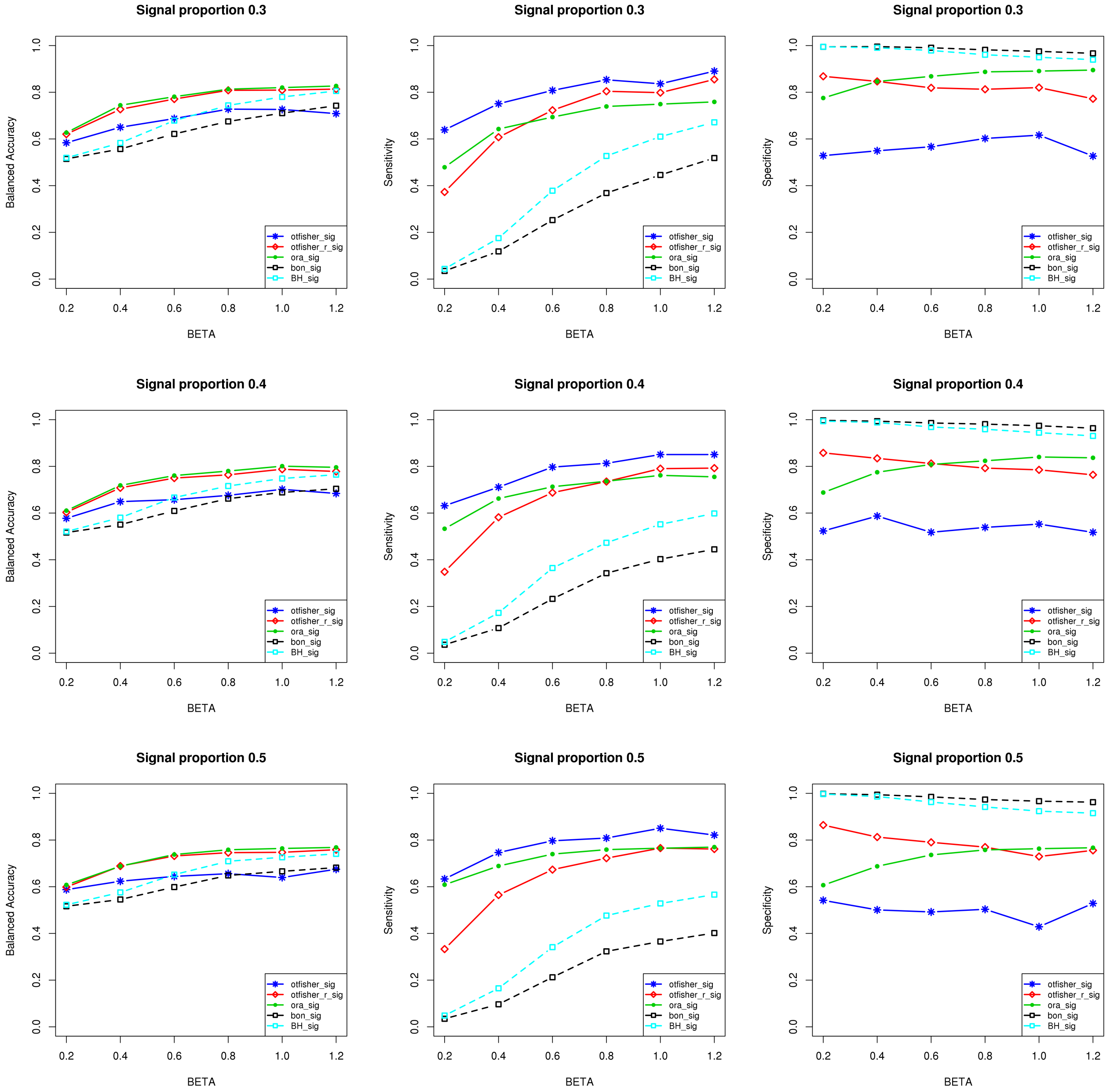

Figure S15: Accuracy at the SNP-screening stage under the continuous trait model. Same setting as Figure S14 except the proportion of causal SNPs in the two causal genes = 0.3 (row 1), 0.4 (row 2), and 0.5 (row 3).

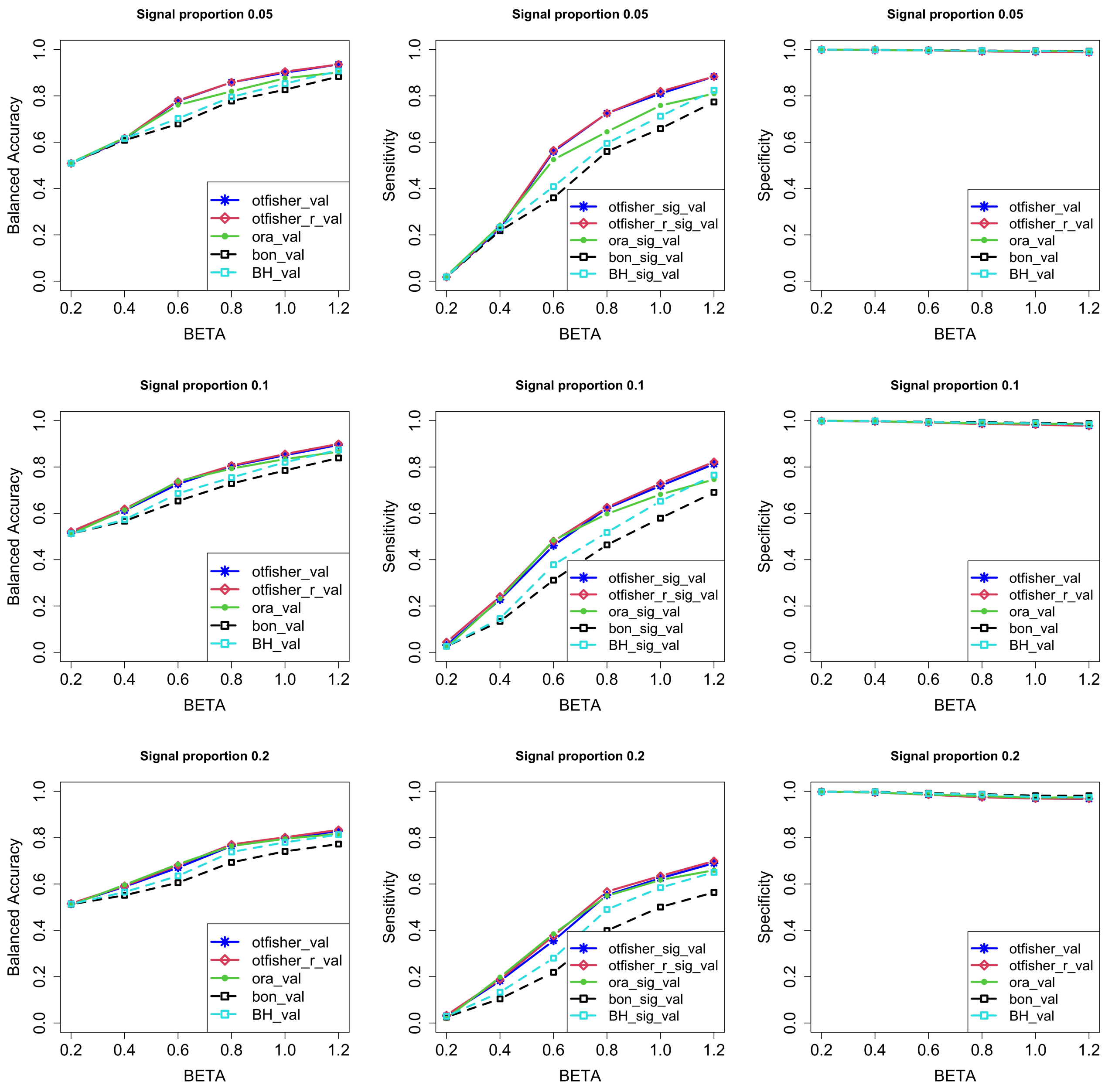

Figure S16: Accuracy at the SNP-validation stage under the continuous trait model. The proportion of causal SNPs in the two causal genes (containing 200 SNPs) = 0.05 (row 1), 0.1 (row 2), and 0.2 (row 3). Accuracy measures: balanced accuracy (column 1), sensitivity (column 2), and specificity (column 3). The search domain in oTFisher is $\tau_1 = \tau_2 \in \{0.01, 0.05, 0.1, 0.2, 0.5, 0.7, 1\}$.

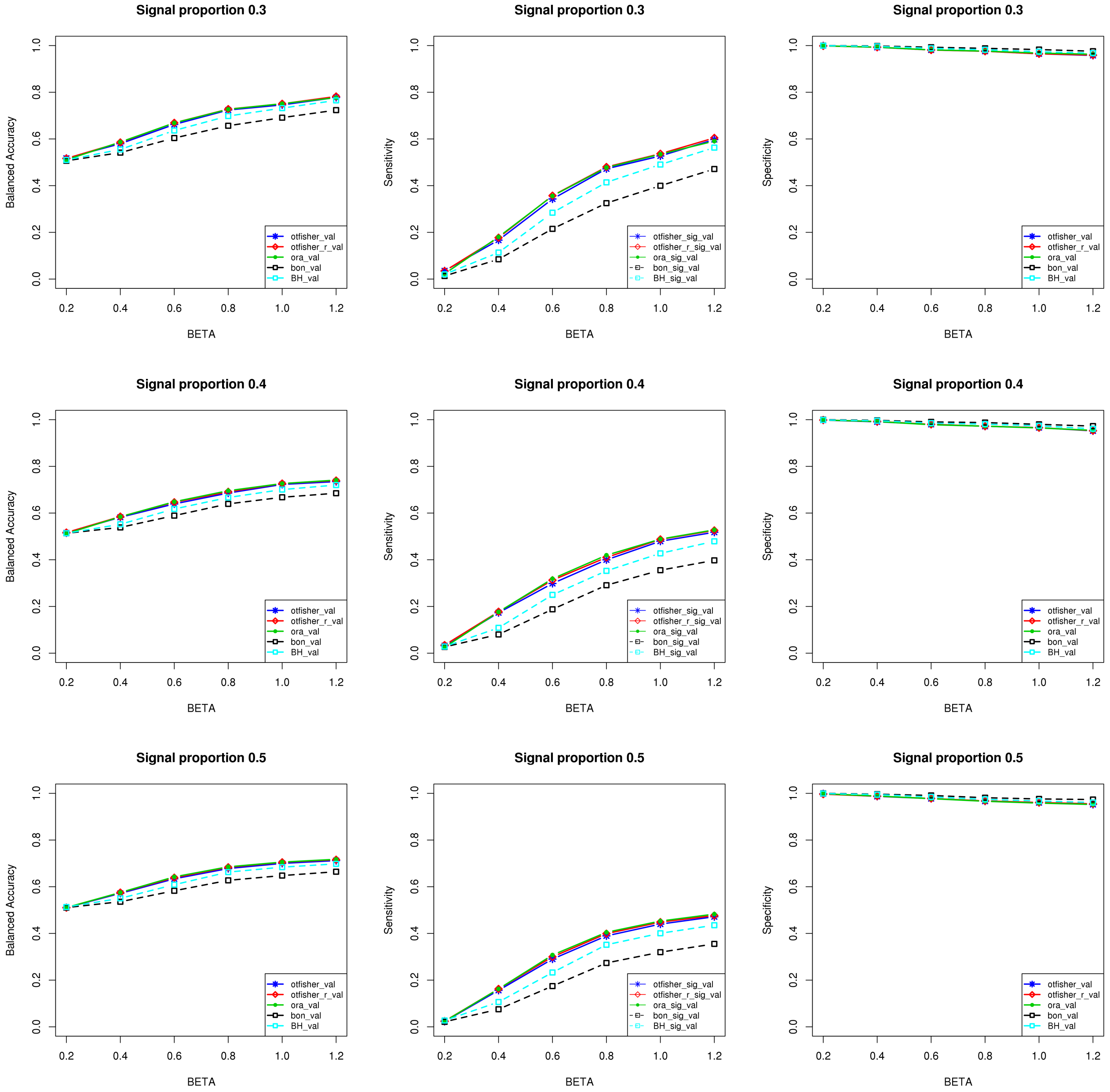

Figure S17: Accuracy at the SNP-validation stage under the continuous trait model. Same setting as Figure S16 except the proportion of causal SNPs in the two causal genes = 0.3 (row 1), 0.4 (row 2), and 0.5 (row 3).

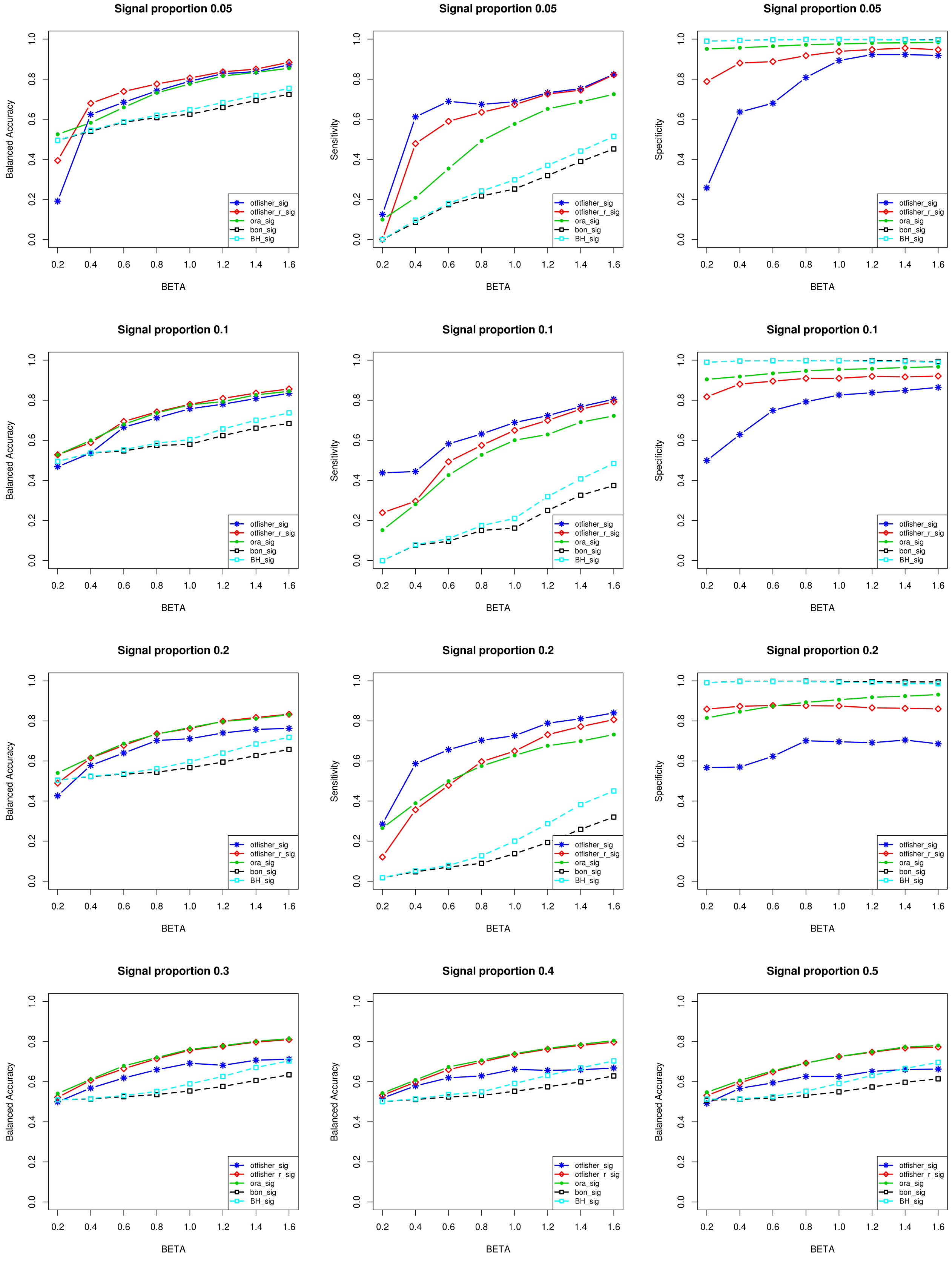

Figure S18: Accuracy at the SNP-screening stage under the binary trait model. The proportion of causal SNPs in the two causal genes (containing 200 SNPs) = 0.05 (row 1), 0.1 (row 2), and 0.2 (row 3). Accuracy measures: balanced accuracy (column 1), sensitivity (column 2), and specificity (column 3). The search domain in oTFisher is $\tau_1 = \tau_2 \in \{0.01, 0.05, 0.1, 0.2, 0.5, 0.7, 1\}$.

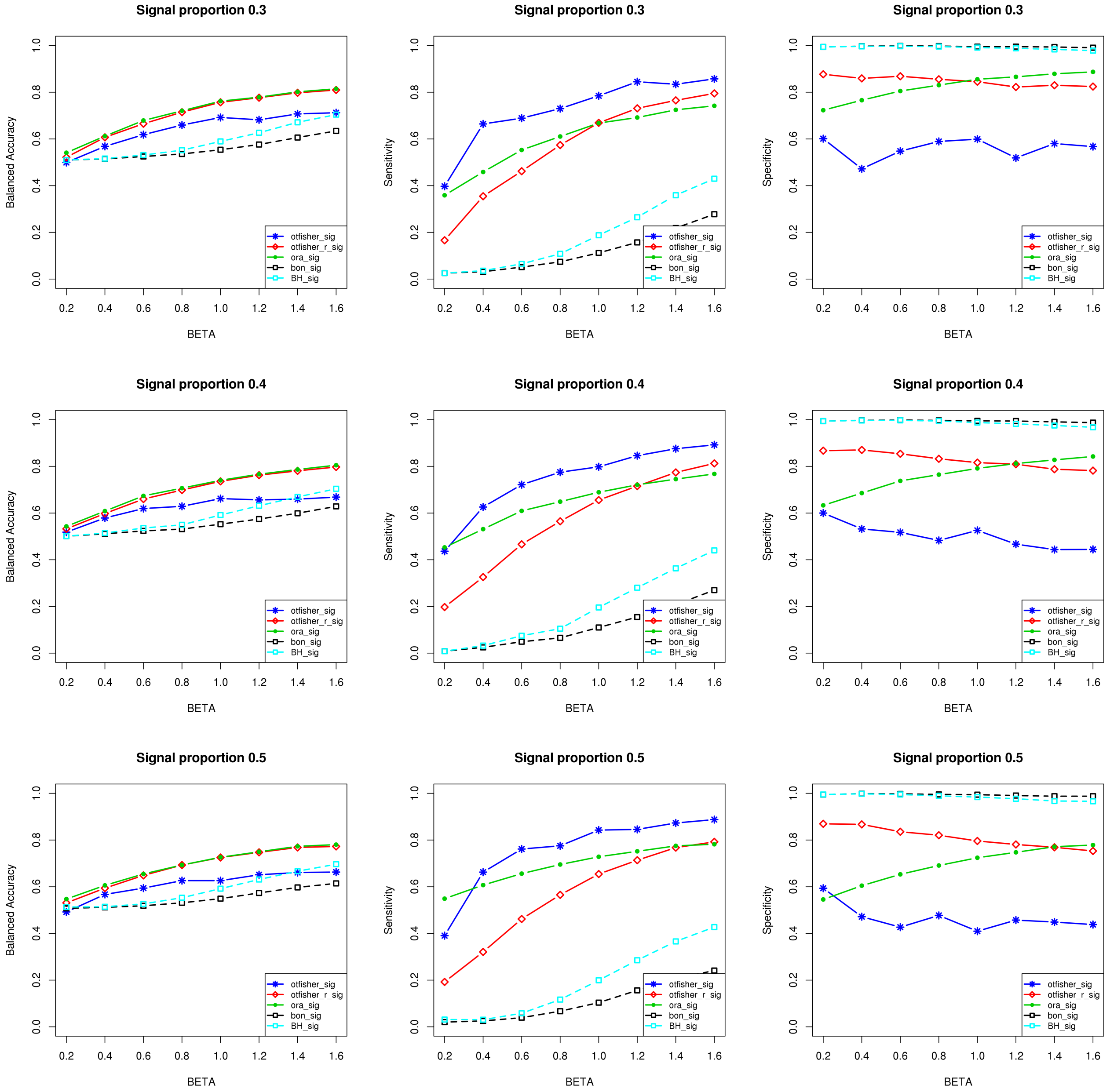

Figure S19: Accuracy at the SNP-screening stage under the binary trait model. Same setting as Figure S18 except the proportion of causal SNPs in the two causal genes = 0.3 (row 1), 0.4 (row 2), and 0.5 (row 3).

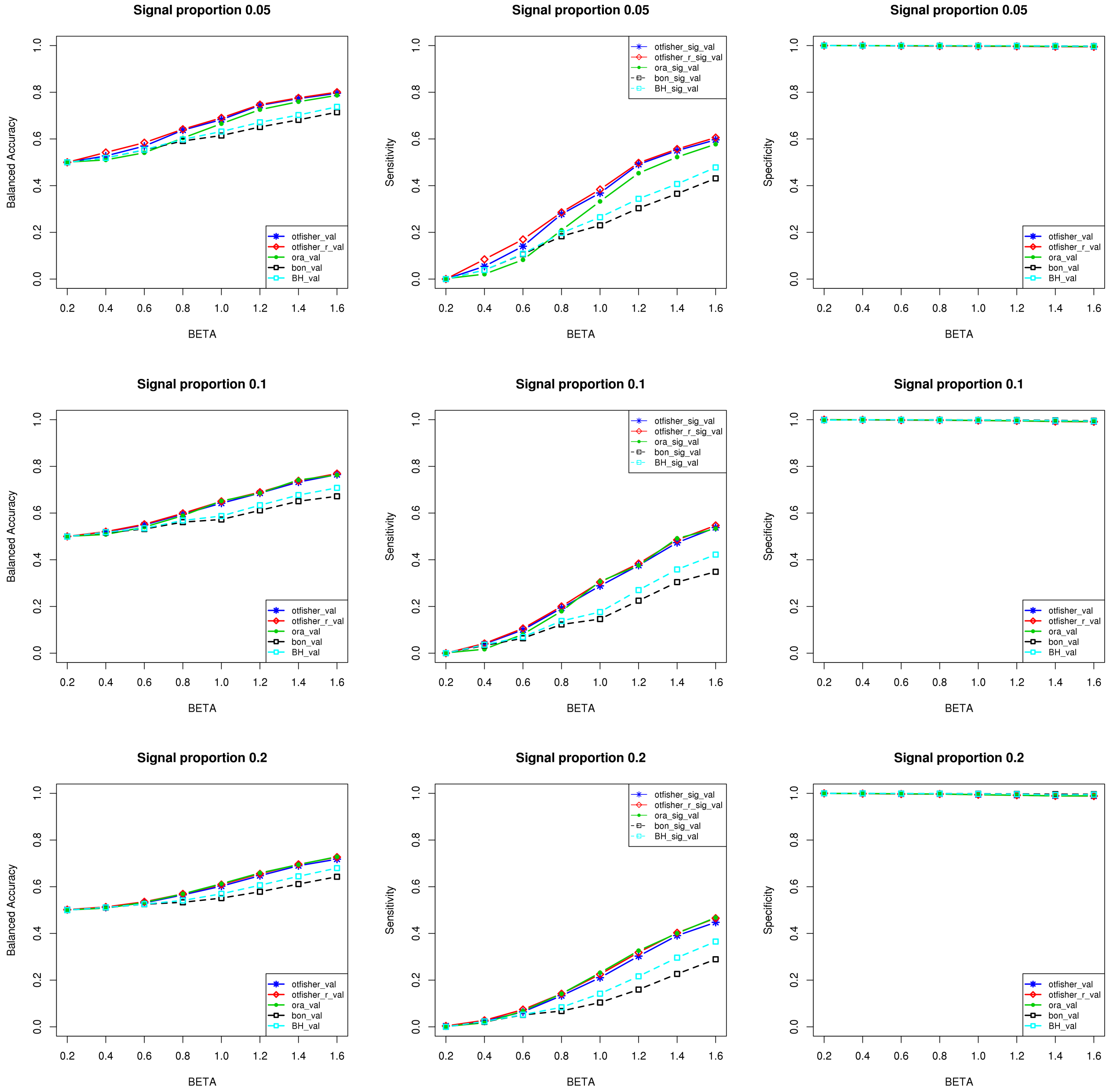

Figure S20: Accuracy at the SNP-validation stage under the binary trait model. The proportion of causal SNPs in the two causal genes (containing 200 SNPs) = 0.05 (row 1), 0.1 (row 2), and 0.2 (row 3). Accuracy measures: balanced accuracy (column 1), sensitivity (column 2), and specificity (column 3). The search domain in oTFisher is $\tau_1 = \tau_2 \in \{0.01, 0.05, 0.1, 0.2, 0.5, 0.7, 1\}$.

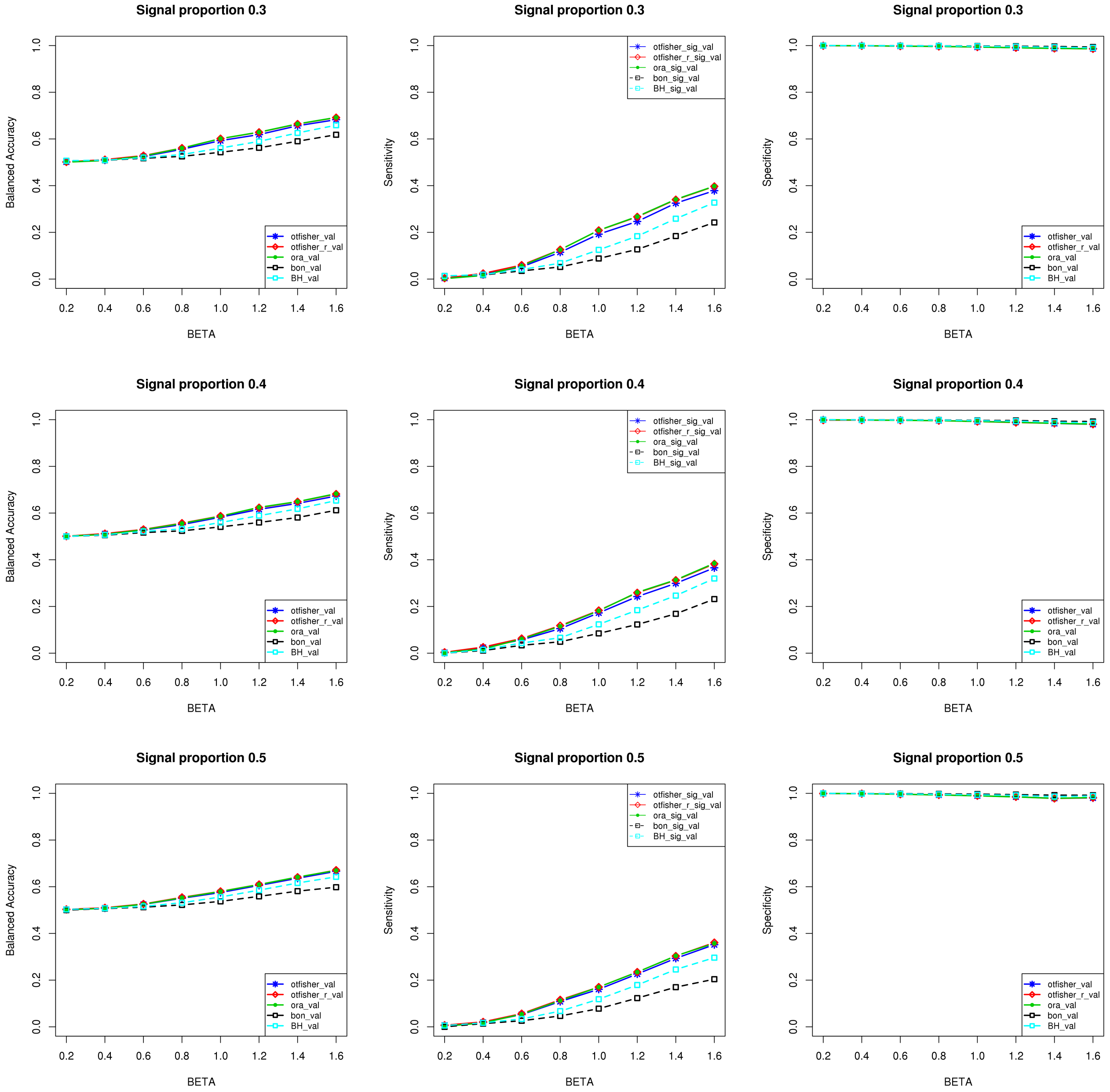

Figure S21: Accuracy at the SNP-validation stage under the binary trait model. Same setting as Figure S20 except the proportion of causal SNPs in the two causal genes = 0.3 (row 1), 0.4 (row 2), and 0.5 (row 3).

3 REAL-DATA ANALYSIS

3.1 GEFOS study and data information

3.1.1 Summary of the GEFOS studies

GEFOS2012_FN and GEFOS2012_LS (Estrada et al., 2012):

- Phenotypes: Bone mineral density (BMD), which defines osteoporosis in the clinic, is the best predictor of fracture risk. This paper studied femoral neck BMD (FN-BMD) and lumbar spine BMD (LS-BMD).

- Genotypes: GWAS genotyping was done by SNP arrays. The MAF information of the real data was not provided, but data provided allele frequency of Allele1 in the HapMap CEU population. We used the pooled data (men and women), including about 2.4M SNPs, including both de novo and imputed genotypes.
- Sample: FN-BMD (stage 1): 32,961 individuals of European and East Asian ancestry. LS-BMD (stage 1): n=31,800. 17 GWAS cohorts (Supplementary Table 18 of (Estrada et al., 2012)): AFOS (Amish Family Osteoporosis Study); AOGC (Anglo-Australasian Osteoporosis Genetics Consortium); CHS (Cardiovascular Health Study); DeCODE (DeCODE Genetics Study); ERF (Erasmus Rucphen Family); EPICNOR (Europen Prospective Investigation into Cancer, Norfolk study); FHS (Framingham Heart Study); GOOD (Gothenburg Osteoporosis and Obesity Determinants Study); HABC (Health Aging and Body Composition); HKOS (Hong Kong Osteoporosis Study); Indiana (Indiana Genetics of Bone Fragility Study); ORCADES (The Orkney Complex Disease Study); RS-I (Rotterdam Study-I); RS-II (Rotterdam Study-II); RS-III (Rotterdam Study-III); TUK-1 (TwinsUK); TUK-23 (TwinsUK).
- Data quality control: SNPs missing in more than three studies were excluded. The data exclude variants with MAF $\leq 1\%$. From the supplementary materials: "Genotyped calls from K-Biosciences, AOGC and deCODE were centrally controlled for: Sample call rate $> 80\%$, SNP call rate $> 90\%$, HWE P $> 1 \times 10^{-4}$, MAF $> 1\%$. The following QC filters were applied for samples genotyped by WHI: Sample call rate $> 98\%$, SNP call rate $> 98\%$, HWE P $> 1 \times 10^{-6}$, MAF $> 0.5\%$."
- SNP association analysis and p-values: SNP p-values were obtained from a meta-analysis using regression coefficients (beta and standard error). "each study ... using sex-specific, age- weight- and principal components- adjusted standardized residuals analyzed under an additive (per allele) genetic model." SNP p-values are two-sided. Data have been made corrections against inflation of test statistics using genomic control at the individual study level and after meta-analysis. Note that SNP p-values downloaded from `http://www.gefos.org/` might be different from p-values reported by the paper. For example, for SNP rs13245690 on chr7, its downloaded p-value is 7.674e-07 and its reported p-value is 1.07E-09; for SNP rs10226308 on chr7, its downloaded p-value is 2.578e-06 and its reported p-value is 3.36e-09. When such a discrepancy happens, we use the p-values reported by the paper.

GEFOS2015_FN, GEFOS2015_LS, and GEFOS2015_FA (Zheng et al., 2015):

- Phenotypes: Femoral neck bone mineral density (FN-BMD), lumbar spine BMD (LS-BMD), and forearm BMD (FA-BMD).
- Genotypes: Meta-analysis of whole genome sequencing (n=2,882 from UK10K), whole exome sequencing (n = 3,549), deep imputation of genotyped samples using a combined UK10K and the 1000Genomes reference panel (n=26,534), and de novo replication genotyping (n=20,271). GEFOS2015_LS data includes 10,582,866 SNPs; GEFOS2015_FA data includes 9,955,365 SNPs.
- Sample: The total sample size is about n = 53k individuals of European ancestry from the general population. Study cohorts: ALSPAC (Avon Longitudinal Study of Parents and Children) and TwinsUK cohorts (for whole-genome sequencing); AOGC, FHS, RS-I, ESP, and ERF cohorts (for exome sequencing).
- Data quality control: Variants with MAF$< 0.5\%$ were excluded.
- SNP association analysis and p-values: "Single variants with a MAF $> 0.5\%$ were tested for an additive effect on the lumbar spine, femoral neck, and forearm BMD, adjusting for sex, age, age2, weight, and standardized... Meta-analysis of cohort-level summary statistics was undertaken using GWAMA..." Note that we only have access to the SNP p-values by their "discovery" stage data. The

associated SNPs reported in the paper could have smaller p-values based on the “combined” data. In particular, SNPs rs11024028 and rs55983207 from GEFOS2015_FN and the SNPs rs188303909 and rs6542457 from GEFOS2015_LS were not genome-wide significant at the discovery stage, but they were genome-wide significant based on the combined data (see their Table 1 (Zheng et al., 2015)). Our SNP-set analysis was based on the SNP p-values at the discovery stage.

GEFOS2017_BMD (Medina-Gomez et al., 2017):

- Phenotypes: Total body less head BMD (TBBMD).
- Genotypes: HapMap imputed data (hg18, build 36) including 2,300,000 SNPs with MAF $\geq$ 0.05 and acceptable imputation quality.
- Sample: A meta-analysis of four pediatric cohorts of population-based studies: the Generation R Study, the Avon Longitudinal Study of Parents and their Children (ALSPAC), the Bone Mineral Density in Childhood Study (BMDCS), and the Copenhagen Prospective Studies on Asthma in Childhood (COPSAC) cohort. The sample size is about 10K in total.
- Data quality control: MAF $\geq$ 0.05 and acceptable imputation quality. Samples were excluded from the analysis to guarantee that no pair of individuals exceeded the standard GCTA cut-off coefficient of 0.025 for genetic relatedness.
- SNP association analysis and p-values: ”Analysis of the two quantitative traits was performed in each study individually based on best guess data using the ”qt- command” available in PLINK...” ”The overall bivariate association test was obtained by a standard Wald-type statistic (chi-square on two d.f.)...”

GEFOS2017_TBBMD (Medina-Gomez et al., 2018):

- Phenotypes: Total body bone mineral density (TB-BMD), overall and divided across five age strata.
- Genotypes: To enable meta-analysis, each study performed genotype imputation using the cosmopolitan (all ethnicities combined) 1000 Genomes phase 1 v.3 (March 2012) reference panel, yielding 30,000,000 SNPs.
- Sample: Thirty epidemiological studies comprised 66,628 individuals from populations across America, Europe, and Australia.
- Data quality control: Filters included: presence in at least two participant studies and MAF 0.5% in these studies.
- SNP association analysis and p-values: TB(LH)-BMD was corrected for age, weight, height, and genomic principal components (derived from GWAS data), as well as any additional study-specific covariates (e.g., recruiting center), in a linear regression model. SNP association was tested for autosomal variants, in which the additive effect of each SNP on the normalized BMD residuals was estimated via linear regression.

GEFOS2018_Fracture (Trajanoska et al., 2018):

- Phenotypes: Study identified the genetic determinants of fracture risk and assessed the role of 15 clinical risk factors on osteoporotic fracture risk.
- Genotypes: GWAS and HapMap-based imputation. 2,539,801 autosomal SNPs.

- Sample: A total of 25 cohorts with genome-wide genotyping and fracture data were recruited globally through the GEnetic Factors for OSteoporosis consortium (GEFOS), comprising 37,857 cases and 227,116 controls. The cohorts were from Europe (n=15), North America (n=8), Australia (n=1), and east Asia (n=1).
- Data quality control: SNPs present in more than two studies with MAF $>$ 5% and acceptable imputation quality (Rsqr $>$ 0.4) were meta-analyzed.
- SNP association analysis and p-values: The study used logistic regression (adjusting for sex, age (simple and quadratic terms), height, and weight) to test the additive (per allele) genetic effects.

GEFOS2020_Falls (Trajanoska et al., 2020):

- Phenotypes: Falling risk.
- Genotypes: GWAS data. Imputation was performed using the Haplotype Reference Consortium (HRC) panel.
- Sample: The 89,076 cases and 362,103 controls were from the UK Biobank (in total 451,179 White European individuals at the ages of 40-69 years). 7,745,930 SNPs.
- Data quality control: Individuals were excluded based on unusually high heterozygosity or $>$ 5% missing genotype rate, or a mismatch between self-reported and genetically inferred sex. SNP exclusions were made based on low MAF $<$ 1% and low imputation quality (info$<$0.3).
- SNP association analysis and p-values: The study used logistic regression adjusting for age and sex.

3.1.2 Summary statistics

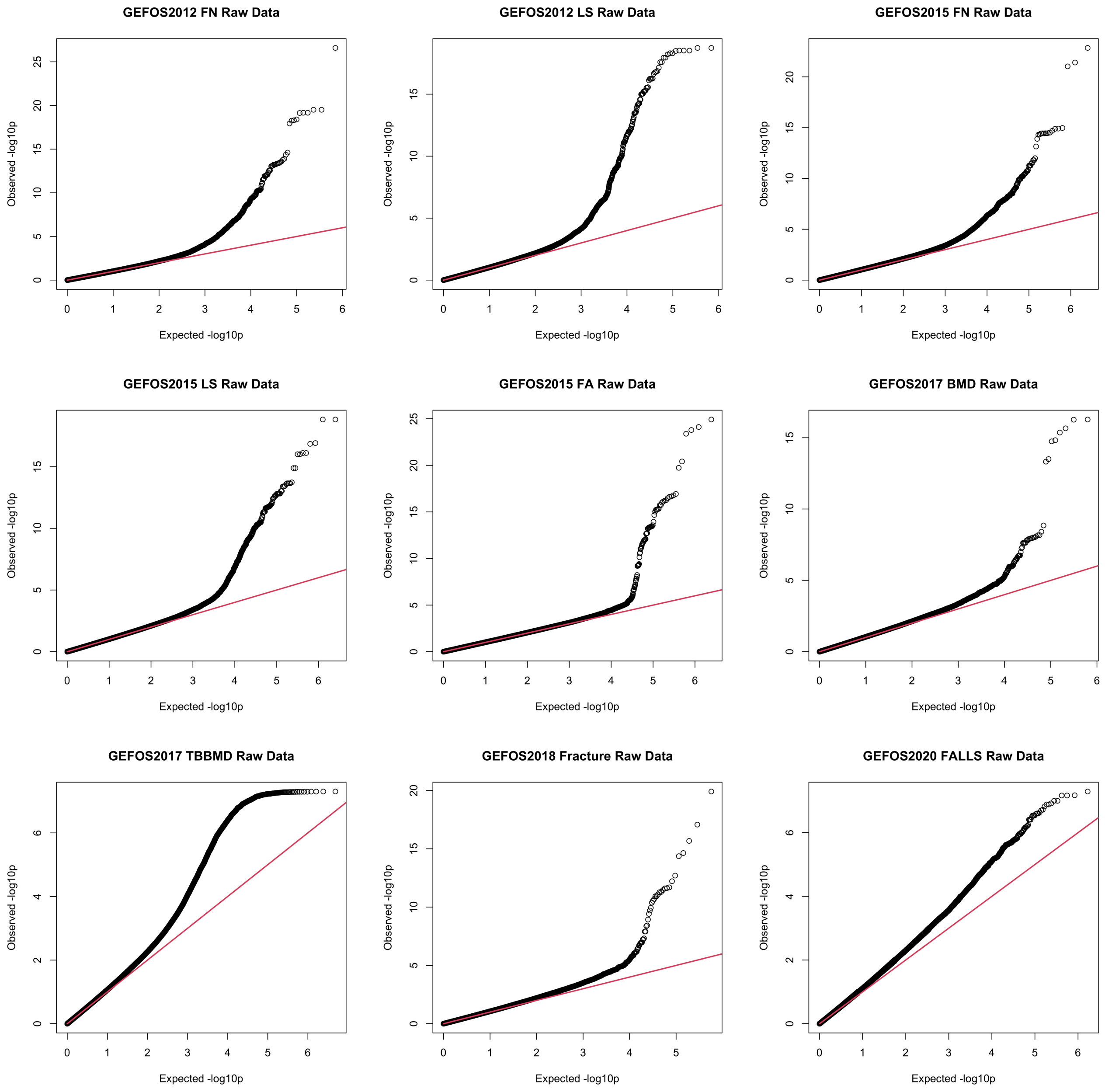

Figure S22: Q-Q plots for raw SNP p-values. GEFOS2012: $\lambda_{2012fn} = 1.00$, $\lambda_{2012ls} = 1.00$; GEFOS2015: $\lambda_{2015fn} = 1.02$, $\lambda_{2015ls} = 1.01$, $\lambda_{2015fa} = 1.02$; GEFOS2017: $\lambda_{2017bmd} = 1.05$, $\lambda_{2017tbbmd \geq 5e-8} = 1.07$; GEFOS2018: $\lambda_{2018frac} = 1.07$; GEFOS2020: $\lambda_{2018frac} = 1.15$.

Table S5. Distribution of the minor allele frequencies (MAFs) of SNPs mapped to genes. No. of SNPs: (No. of mapped SNPs / total No. of SNPs); Rare SNPs: MAF<0.05.

No. of SNPs	Min.	1st Qu.	Median	Mean	3rd Qu.	Max.	Prop. of rare SNPs
2012FN (695577 / 2454680)	0.0102	0.1080	0.2158	0.2312	0.3496	0.5000	0.0972
2012LS (691135 / 2482597)	0.0101	0.1008	0.2044	0.2243	0.3423	0.5000	0.1080
2015FN (2521984 / 10586899)	0.0001	0.0100	0.04489	0.1169	0.1912	0.5000	0.5169
2015LS (2523883/ 10582866)	0.0001	0.0100	0.0448	0.1168	0.1910	0.5000	0.5173
2015FA (2454680 / 9955365)	0.0001	0.0120	0.0536	0.1242	0.2073	0.5000	0.4886
2017BMD (495139 / 2276829)	0.0502	0.1242	0.2296	0.2457	0.3587	0.5000	0
2017TBBMD (4210008 / 18259434)	0.0050	0.0153	0.0329	0.0951	0.1187	0.5000	0.6001
2018Fracture (571714 / 2519953)	0.0050	0.0765	0.1873	0.2105	0.3337	0.5000	0.1702
2020FALLS (1631020 / 7745390)	0.0100	0.0221	0.0647	0.1335	0.2176	0.5000	0.4473

Table S6. Distribution of gene size by the numbers of SNPs contained in genes.

Data Sets	Min.	1st Qu.	Median	Mean	3rd Qu.	Max.
2012FN	1	6	12	27.39	25	3874
2012LS	1	6	12	30.26	25	3844
2015FN	1	23	44	103	97	4267
2015LS	1	24	44	98.68	97	1997
2015FA	1	24	44	101.4	93	11199
2017BMD	1	5	8	20.68	17	2753
2017TBBMD	2	53	94	184.3	191	9585
2018Fracture	1	6	10	24.85	21	3288
2020FALLS	1	18	32	69.75	66	4587

Table S7. Distribution of gene size by the number of blocks. The last column summarizes the No. of blocks mapped to genes / total No. of blocks. Roughly half of the blocks can be mapped to genes.

Data Sets	Min.	1st Qu.	Median	Mean	3rd Qu.	Max.	No. of blocks to genes
2012FN	1	2	4	10.90	9	1690	219900/426744
2012LS	1	2	4	10.89	9	1689	219756/426409
2015FN	1	1	10	27.71	28	3529	578620/1081574
2015LS	1	1	10	27.67	28	3525	577855/1080218
2015FA	1	1	9	24.65	24	3296	517700/979645
2017BMD	1	1	3	7.78	7	1110	144861/293310
2017TBBMD	1	16	34	75.24	73	7639	1627786/3093340
2018Fracture	1	1	3	8.80	7	1288	173938/336188
2020FALLS	1	1	6	16.5	15	2519	345909/666387

Table S8. Haploblock sizes are measured by the number of SNPs per block in our data analysis. The numbers in the first column: (the numbers of SNPs in our data analysis / total numbers of original SNPs). The SNPs used in our data analysis are common SNPs (following the default for estimating haploblocks (Chang et al., 2015); see Genomic Mapping below) minus SNPs trimmed due to high LD (see Correlation Estimation below).

No. of SNPs in Data Analysis	Min.	1st Qu.	Median	Mean	3rd Qu.	Max.
2012FN (1353968/2475350)	1	1	2	5.90	5	290
2012LS (1352939/ 2468080)	1	1	2	5.90	5	290
2015FN (4597697/ 10586899)	1	1	1	8.91	1	1212
2015LS(4596776/ 10586866)	1	1	1	8.92	1	1201
2015FA (4347743/ 9955365)	1	1	1	9.24	2	1196
2017BMD (896648/ 2276829)	1	1	2	7.00	7	283
2017TBBMD (10715260/ 18259434)	1	1	1	5.30	1	1936
2018Fracture (1100018/ 2519953)	1	1	2	7.68	7	342
2020FALLs (3150775/ 7745390)	1	1	1	11.61	4	1069

3.2 Data processing

3.2.1 Genome version, genes, and haploblocks

We unified the genome version to hg19. SNPs that were annotated by a different genome version were converted to hg19 by "snptracker" (Deng et al., 2016) and LiftRsNumber.py (`https://genome.sph.umich.edu/wiki/LiftRsNumber.py`). More than 99.9% SNPs were successfully converted.

We grouped the SNPs into sets by UCSC genes if SNP locates within ±5k upstream and downstream of genes (Chang et al., 2015).

SNPs are also grouped into sets by haploblocks. We estimated haploblocks following the default setting of PLINK (`https://www.cog-genomics.org/plink/1.9/ld`). Specifically, the procedure uses common SNPs (MAF ≥0.05) with SNP pairs within 200 kbs of distance. A pair of SNPs are considered in "strong LD" if the bottom of the 90% D-prime confidence interval is greater than 0.70, and the top of the confidence interval is at least 0.98. It treats confidence interval tops smaller than 0.90 as strong evidence for historical recombination. The number of strong LD pairs within a haploblock must be more than 0.95 times

the total number of informative pairs (i.e., either "strong LD" or 'recombination'). Our SNP-set analysis added rare SNPs (MAF $<$ 0.05) back to haploblocks based on genomic locations. To avoid losing genetic information, we still contain into study the individual SNPs that are not assigned into blocks with other SNPs. We treated them as blocks of single representative SNPs and used SNP p-values as the p-values of the blocks.

3.2.2 LD estimation, correlations of SNP statistics, and SNP pruning

The LDs among SNPs were estimated using the genotype data of the 1000 Genomes Projects Phase 3 (1KG) (Lin and Zeng, 2010). The choices of the 1KG subjects for LD calculation were consistent with GEFOS studies from which the summary data were obtained. For example, if most individuals in a GEFOS study were of European and Asian ancestry, we would estimate the LD by using the genotype data of non-African samples of the 1KG.

Based on the original studies that generated the summary data, it is reasonable to assume that the SNP p-values' corresponding statistics $\mathbf{Z}$ in the manuscript equation (4) are asymptotically normal distributed. Following the common practice in literature, we estimated their correlation matrix Σ by the matrix of LD r values.

To achieve computational stability in SNP-set analysis, we trim high LDs (i..e, prune SNPs) to avoid ill-conditioned Σ. Precisely, if a SNP pair has $r^2 > 0.9$, we would arbitrarily remove the SNP with a smaller MAF. SNP pruning is justifiable in SNP-set analysis. First, SNPs in high LD represent the same genetic variant since they are likely transmitted together. Second, SNPs in high LD have highly correlated SNP p-values (see Figure S23 for evidence)]. Therefore, pruning SNPs in high LD does not significantly lose information. We consider that the two SNPs in high LD are atlas to each other. Therefore, detecting either one indicates the finding of the same genetic variant (to be consistent in comparison with the results of the original GEFOS studies).

If Σ was still not positively definite after SNP pruning, it would be recalculated to get the nearest symmetric positive semidefinite matrix with unit diagonal (Higham, 2002).

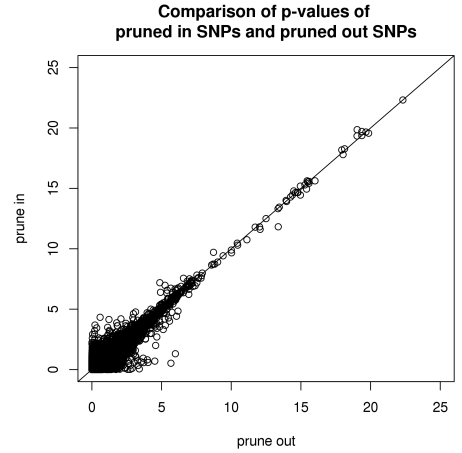

Figure S23: The transformed (-log10) p-values of the pruned SNPs vs. their counterparts in high LD (GEFOS2012FN data).

3.2.3 Adjust SNP p-values

The SNP p-values from some summary data sets might show inflated Q-Q plots. The inflation could be due to confounding biases, which need to be reduced before the p-combination tests. To adjust the SNP p-values, we first adjust their Z-scores by utilizing the LD score regression (LDSR) approach, which helps distinguish potential confounding biases from polygenicity (Bulik-Sullivan et al., 2015). Specifically, for the Z-score Z_j of the jth SNP, it has been shown an approximate relationship: $E(Z_j^2) = Na + 1 + \beta l_j$, where $\beta = Nh^2/n$, N is the sample size, n is the number of SNPs such that h^2/n is the average heritability explained per SNP, a measures the contribution of confounding biases, and l_j is the LD score of SNP j. When no confounding biases exist, we denote the Z-score Z_j^* and, by the above equation, $E(Z_j^{*2}) = 1 + \beta l_j$. We consider the fixed-effect model, i.e., the genetic effects are fixed unknown parameters in the regression (Lee et al., 2018). Since they are standardized statistics, the Z-scores have the unit variance (maybe in the asymptotic sense). So, $(E(Z_j))^2 = E(Z_j^2) - Var(Z_j) = E(Z_j^2) - 1 = Na + \beta l_j$, and similarly, $(E(Z_j^*))^2 = \beta l_j$. Following these equations, we have $E(Z_j^*) = sgn(Z_j^*)\sqrt{1 + (E(Z_j^*))^2 - 1} = sgn(Z_j^*)\sqrt{\frac{((E(Z_j))^2+1)(1+\beta l_j)}{1+Na+\beta l_j} - 1}$. Using the observed Z-score from a given data to approximate the expectation, we get the adjustment relationship:

$$Z_j^* = sgn(Z_j)\sqrt{\frac{(Z_j^2 + 1)(1 + \beta l_j)}{1 + Na + \beta l_j} - 1}. \tag{S3}$$

We keep the signs of the Z-scores consistent, but that does not matter when their p-values are two-sided. The equation makes sense in that $Z_j^* = Z_j$ when no confounding biases exist, e.g., $a = 0$. We remain $Z_j^* = Z_j$ unchanged when $\frac{(Z_j^2+1)(1+\beta l_j)}{1+Na+\beta l_j} - 1 < 0$, which indicates confounding might not exist and / or the estimation of confounding is not accurate. We get the adjusted Z scores for all SNPs and convert them to p-values for downstream analysis. In our data analyses, we applied software LDSC and the script ldsc.py (`https://github.com/bulik/ldsc`) to estimate HapMap3 LD scores (using the genotype data from the 1000 Genomes Project), the intercept $1 + Na$, and h^2.

3.2.4 Literature-reported SNPs and genes

The list of literature SNPs:

Supplementary file literature_snps.xlsx includes 4,802 unique SNPs reported in the literature to be associated with osteoporosis, BMD-related traits, and bone fracture. These SNPs were queried from

- GWAS Catalog, by using trait terms 'osteoporosis', 'radius bone mineral density', 'hip bone mineral density', 'pelvis bone mineral density', 'trunk bone mineral density', 'spine bone mineral density', 'volumetric bone mineral density', 'heel bone mineral density', 'femoral neck bone mineral density', 'abnormality of bone mineral density', and 'bone fracture'.
- Open Target Genetics, by using trait terms 'osteoporosis' and 'bone mineral density'.

The original sources used Genome Assembly GRCh38.p13 and dbSNP Build154. We have converted the SNP positions to GRCh37(hg19).

Last update: May 15, 2022.

The list of literature genes:

Supplementary file literature_genes.xlxs includes 2,179 unique genes that were reported in the literature to be associated with osteoporosis, BMD-related traits, and bone fracture. These genes were queried from

- GWAS Catalog, by using trait terms 'osteoporosis', 'radius bone mineral density', 'hip bone mineral density', 'pelvis bone mineral density', 'trunk bone mineral density', 'spine bone mineral density', 'volumetric bone mineral density', 'heel bone mineral density', 'femoral neck bone mineral density', 'abnormality of bone mineral density', and 'bone fracture'.
- Open Target Genetics, by using trait terms 'osteoporosis' and 'bone mineral density'. [Search results include "nearest genes" and "L2G genes".]
- Malacards, by using trait terms 'osteoporosis'.
- Manual Google search for literature articles, by using key word 'bone mineral density'.

Among these 2179 genes, 1420 genes were given by GWAS catalog (in its "reported gene" column) using the above literature SNPs, additional 229 genes were obtained from Malacards, additional 293 and 204 genes were obtained from Open Targets Genetics (based on its "closest gene" column and the Locus-to-Gene (L2G) pipeline, respectively), and additional 33 genes were obtained from other literature articles searched by Google using key word "bone mineral density".

The file also includes 16 genes for trait 'falling risk' reported by (Trajanoska et al., 2020).

Last update: May 15, 2022.

3.3 Gene-based analysis

3.3.1 Q-Q plots

The Q-Q plots of the gene-based association p-values.

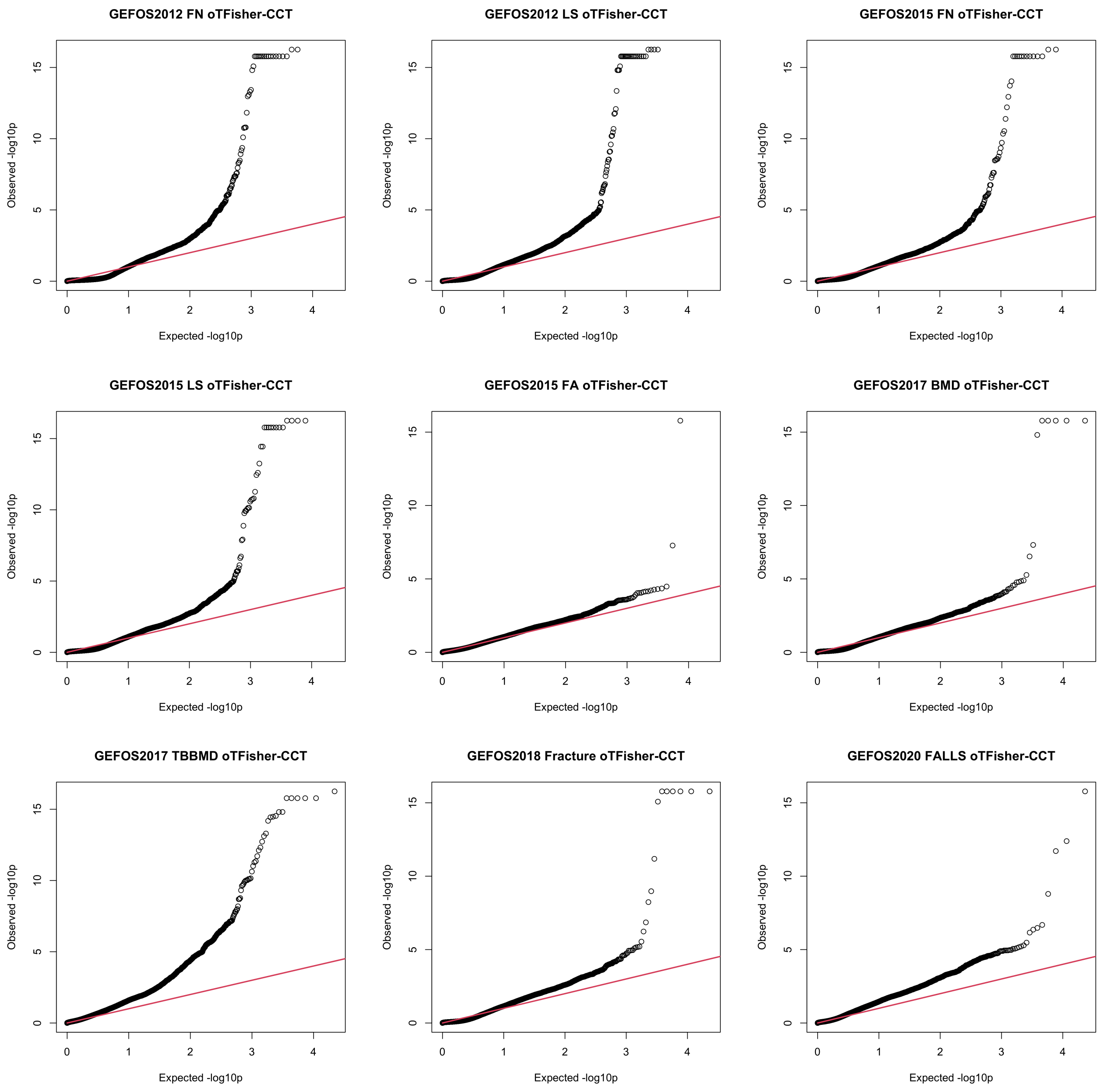

Figure S24: Q-Q plots for the gene-based association p-values by oTFisher_cct. Genetic inflation factors: GEFOS2012: $\lambda_{2012fn} = 0.12, \lambda_{2012ls} = 0.19$; GEFOS2015: $\lambda_{2015fn} = 0.25, \lambda_{2015ls} = 0.24, \lambda_{2015fa} = 0.59$; GEFOS2017:$\lambda_{2017bmd} = 0.21, \lambda_{2017tbbmd} = 1.34$; GEFOS2018: $\lambda_{2018frac} = 0.28$; GEFOS2020: $\lambda_{2020falls} = 0.91$.

3.3.2 Top-hit genes

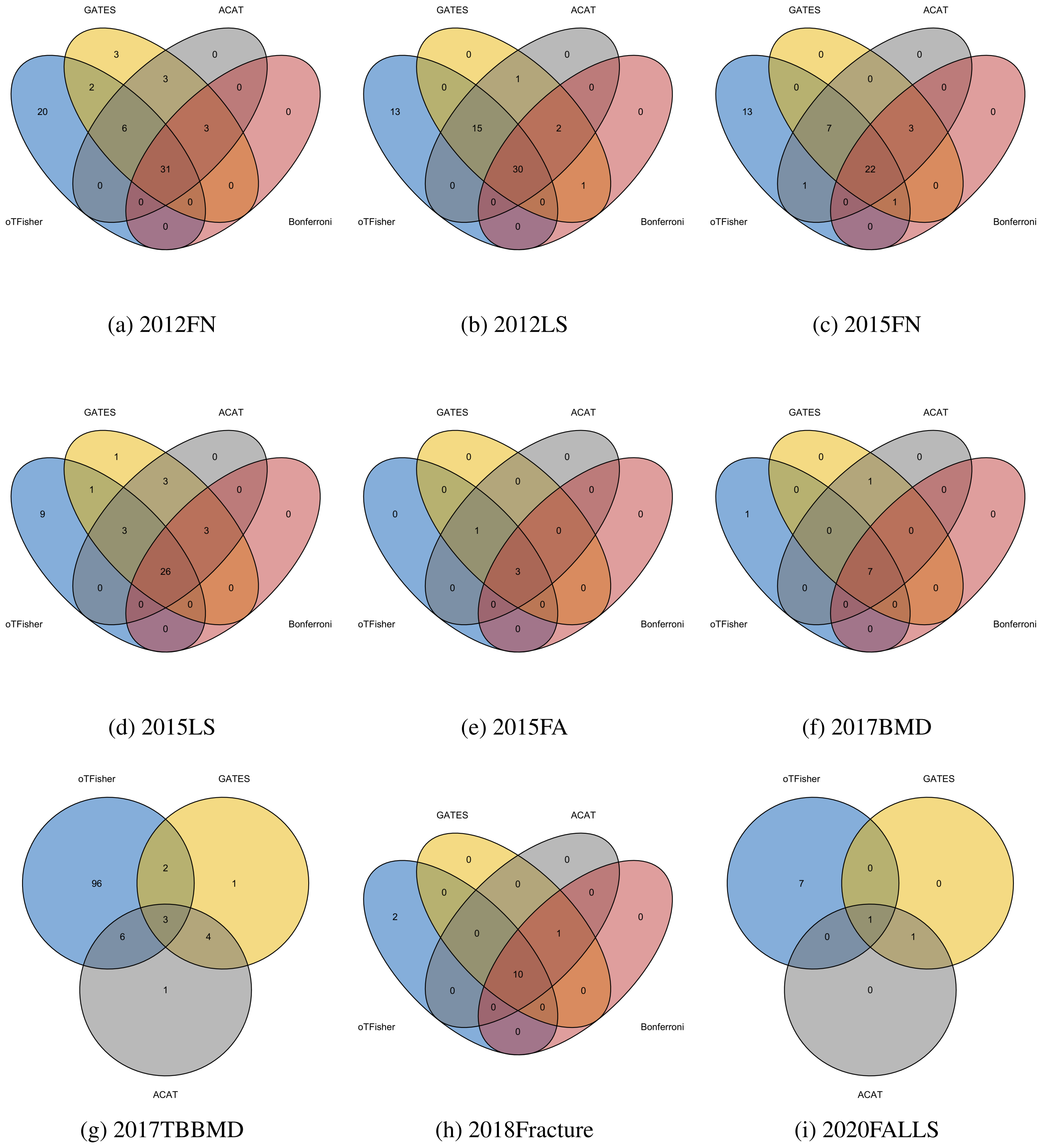

Figure S25: Venn diagrams for the overlap of top-hit genes found by four SNP-set tests: oTFisher_cct, Bonferroni, ACAT, and GATES.

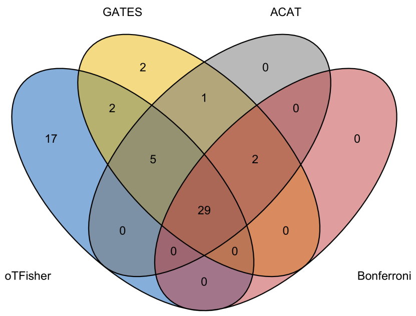

(a) 2012FN

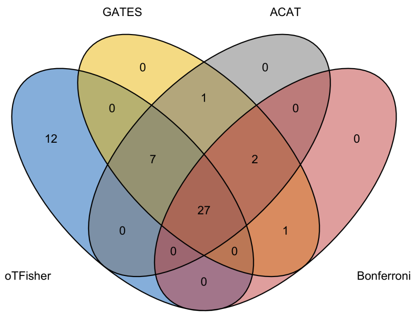

(b) 2012LS

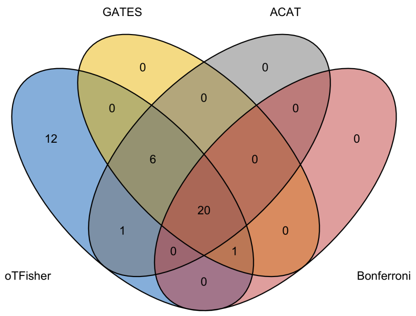

(c) 2015FN

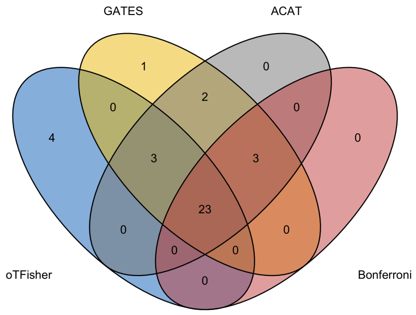

(d) 2015LS

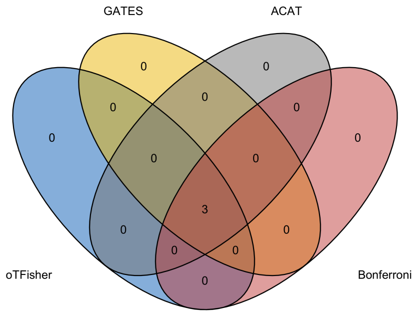

(e) 2015FA

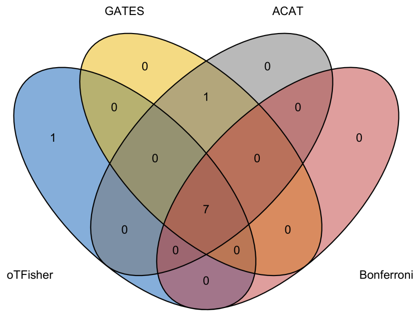

(f) 2017BMD

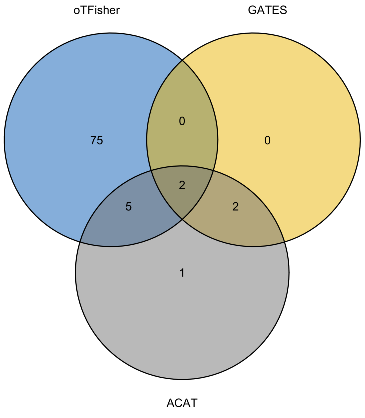

(g) 2017TBBMD

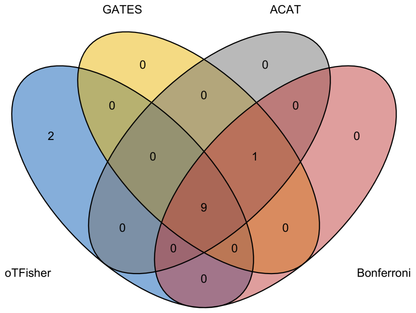

(h) 2018Fracture

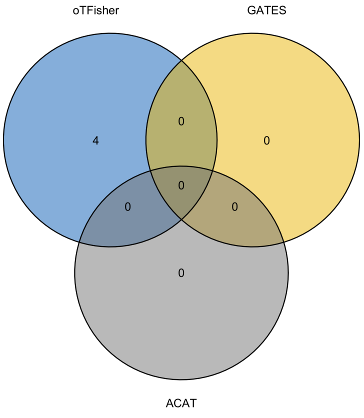

(i) 2020FALLS

Figure S26: Venn diagrams for the overlap of top-hit literature genes. Methods: oTFisher_cct, Bonferroni, ACAT, and GATES.

Table S9. SNP distribution for part of top-hit genes on chromosome 1 (based on the GEFOS2017_TBBMD data). The 5% and 10% percentiles of SNP p-values are smaller than the expected values under the null.

chr	gene	size	min	5 %	10 %	oTFisher_cct	oTFisher_minp
1	WLS	545	5.18E-08	0.00010248	0.002441	2.41E-10	6.21E-10
1	CTPS1	133	2.27E-07	1.00E-05	0.0037342	2.86E-07	1.66E-06
1	HSPG2	495	2.87E-06	0.00020673	0.0020232	1.18E-07	1.14E-07
1	MIR1262	30	7.94E-08	1.10E-05	0.00010747	4.60E-07	5.27E-07
1	RAP1GAP	362	1.44E-06	0.00025258	0.0023815	1.03E-07	5.76E-07
1	SLC30A10	86	5.17E-08	0.00086298	0.0017305	1.04E-07	1.67E-07
1	FAAH	88	2.34E-06	1.28E-05	0.00017442	4.39E-07	1.12E-06
1	CDC42	150	5.23E-08	3.81E-05	0.0030756	4.83E-13	6.23E-13

Table S10. SNP distribution for top-hit genes based on the GEFOS2020_FALLS data. The 5% and 10% percentiles of SNP p-values are smaller than the expected values under the null.

chr	gene	size	min	5 %	10 %	oTFisher_cct	oTFisher_minp
1	FAM212B	75	2.60E-07	3.43E-05	0.000156	3.34E-07	8.47E-07
2	RAPH1	63	1.40E-05	0.000141	0.000814	4.37E-07	4.42E-07
11	BDNF-AS	114	1.00E-07	2.48E-06	0.000294	1.95E-12	8.85E-12
11	BDNF	49	2.50E-07	3.68E-06	0.00014	1.67E-16	6.74E-16
11	TSPAN4	57	5.10E-08	2.44E-06	0.0003252	4.07E-13	1.04E-13
14	LINC00871	383	5.70E-07	2.61E-05	0.000886	6.95E-07	1.09E-07
16	RAB11FIP3	115	5.40E-06	3.46E-05	5.46E-05	2.09E-07	1.62E-07
19	LOC284395	381	1.60E-06	7.00E-06	0.005	1.61E-09	2.51E-09

3.3.3 Putative novel genes

The significantly enriched GO/pathway terms contain many literature genes, indicating the results are reliable. At the same time, the top-hit novel genes included in the significantly enriched GO/pathway terms are likely relevant to the traits through the corresponding functions of these GO/pathway terms. The following is a brief discussion on the relevance of these genes.

Gene *STH* (also named *MAPTIT*) is in the significant GO term cytoplasm (2012LS). It is associated with osteoarthritis-hip through SNP rs62063281 (Tachmazidou et al., 2019).

Gene *RANBP1* in the significant GO term protein binding (2015LS) has shown an association with osteosarcoma (Gemoll et al., 2015).

From the significant GO term protein binding (2017TBBMD), four novel genes *HSPG2*, *BOLL*, *PRPH*, and *MAP3K12* are relevant. More specifically, *HSPG2* was shown to be related to segregating developmental dysplasia of the hip (Basit et al., 2017). *BOLL* (also named *BOULE*) was shown associated with bone marrow (Li et al., 2017). *PRPH* was shown associated with amyotrophic lateral sclerosis (Gros-Louis et al., 2004). *MAP3K12* was shown associated with Lissencephaly type 3 - metacarpal bone dysplasia (`https://platform.opentargets.org/evidence/ENSG00000139625/`

Orphanet_86822) and Infantile osteopetrosis with neuroaxonal dysplasia (https://platform.opentargets.org/evidence/ENSG00000139625/Orphanet_85179).

Gene *PRKAG1* in the significant GO term protein binding (2017TBBMD) has shown an association with bone marrow failure syndrome (https://platform.opentargets.org/evidence/ENSG00000181929/MONDO_0000159).

Gene *RMND1* in the significant GO term protein binding (2017TBBMD) has shown an association with heel bone density through SNP rs373421938 (Kim, 2018).

Gene *SCMH1* in the significant GO term gene silencing (2017TBBMD) has shown an association with breast adenocarcinoma (https://platform.opentargets.org/evidence/ENSG00000010803/EFO_0000304).

Gene *SOX1* in the significant GO term positive regulation of transcription by RNA polymerase II (2015LS) has shown an association with osteosarcoma (Ahmad et al., 2017).

Gene *SPPL2C* in the significant GO term protein binding (2012LS) has shown an association with osteoarthritis through SNP rs62063281(Tachmazidou et al., 2019).

3.4 Haplotype block-based analysis

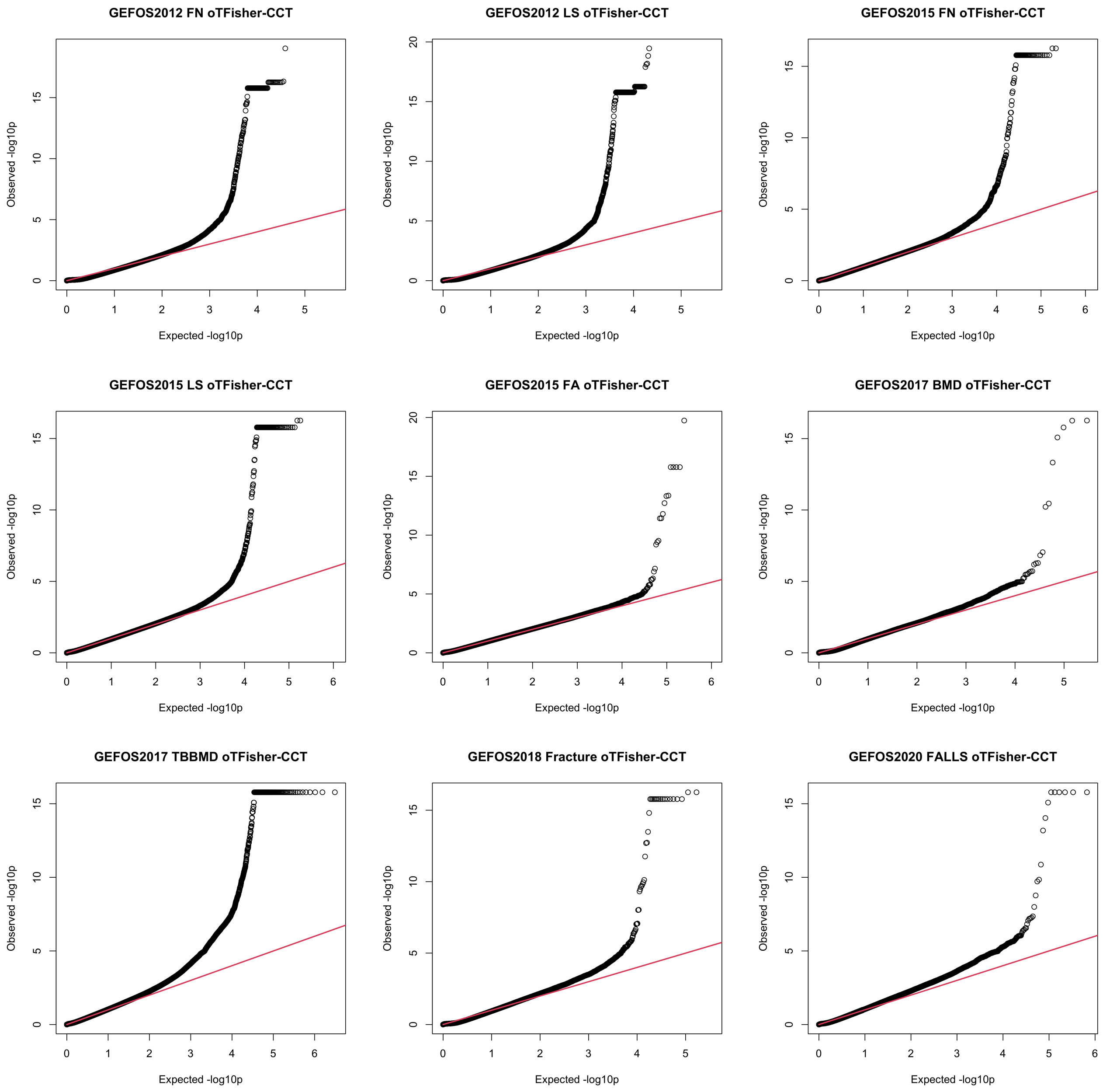

Figure S27: Q-Q plots for the haploblock based analysis by the oTFisher. Genetic inflation factors: GEFOS2012: $\lambda_{2012fn} = 0.2043, \lambda_{2012ls} = 0.2047$. GEFOS2015:$\lambda_{2015fn} = 0.6665, \lambda_{2015ls} = 0.6631, \lambda_{2015fa} = 0.6348$. GEFOS2017: $\lambda_{2017bmd} = 0.3321, \lambda_{2017tbbmd} = 0.7593$. GEFOS2018: $\lambda_{2018fracture} = 0.2937$. GEFOS2020: $\lambda_{2020falls} = 0.6044$.

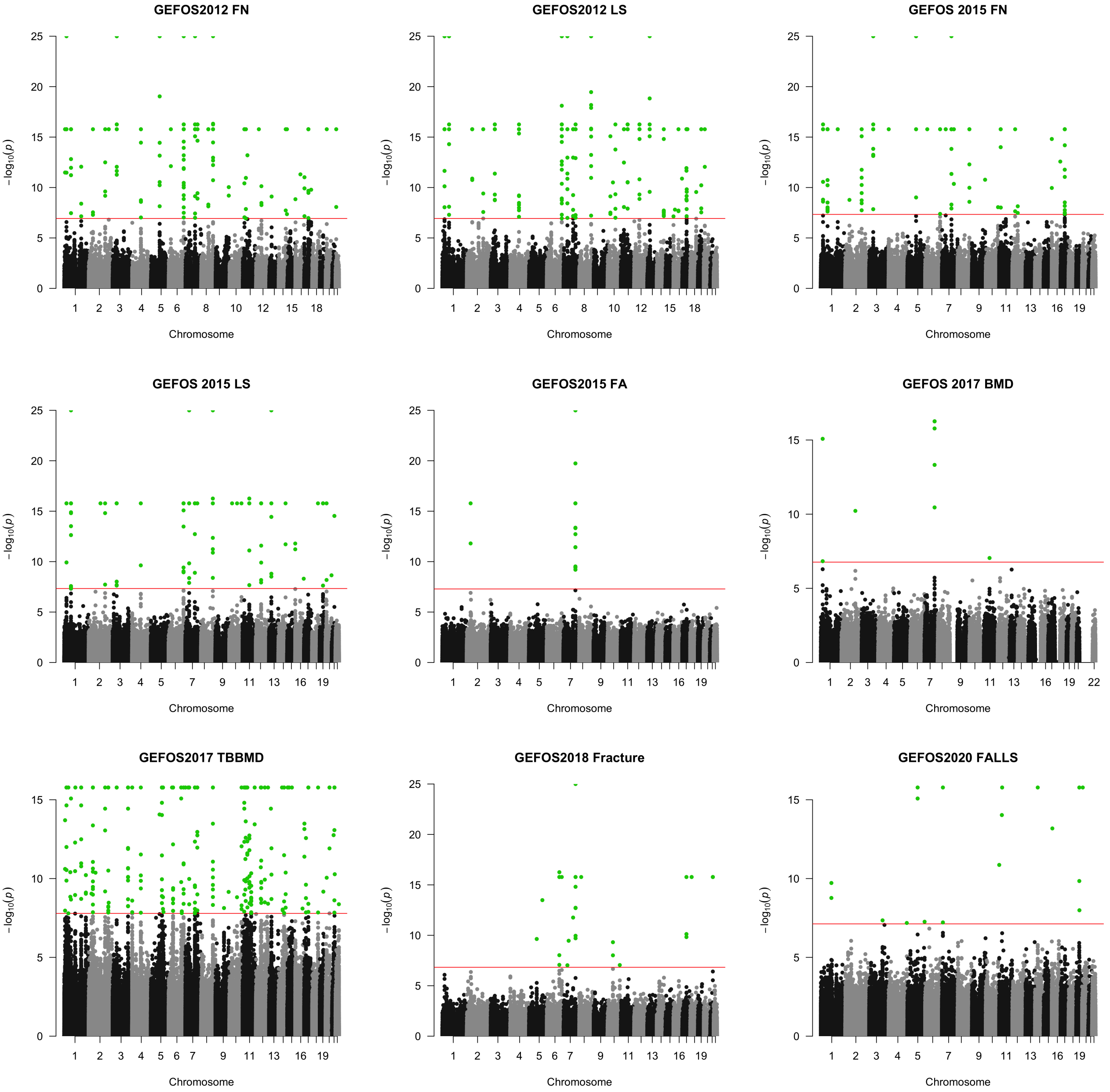

Figure S28: Manhattan plots for haploblock SNP-set test by the oTFisher_cct. The Red line is at a genome-wide significant level -log10(0.05/ total block number). Green dots are significant blocks. Zero p-values were set to 1e-25 for demonstration purposes.

Table S11. The number of top-hit blocks and mapped genes obtained by haploblock-based SNP-set analysis. Literature blocks are these overlapped literature genes or contained literature SNPs. Novel blocks are these not related to literature genes nor literature SNPs. Depending on GEFOS samples, we applied all-population (allpopns) or non-African (noafr) samples of the 1000Genome data to estimate the LD. The number in the parenthesis is the number of blocks that can be mapped to the corresponding gene category.

	blocks			genes		
	literature	novel	total	literature	novel	total
FN-2012-5k: totally 426744 blocks(allpopns)						
oTFisher_cct	109	39	148	45(75)	9(10)	54(85)
GATES	75	32	107	22(53)	2(3)	24(56)
ACAT	77	34	111	23(55)	2(3)	25(58)
Bonferroni	78	30	108	31(57)	2(3)	33(60)
LS-2012-5k: totally 426409 blocks (allpopns)						
oTFisher_cct	135	62	197	53(97)	13(13)	66(110)
GATES	109	53	162	35(82)	4(4)	39(86)
ACAT	113	54	167	35(85)	4(4)	39(89)
Bonferroni	108	50	158	35(80)	4(5)	39(85)
FN-2015-5k: totally 1081574 blocks(noafr)						
oTFisher_cct	65	27	92	30(46)	5(7)	35(53)
GATES	46	21	67	22(31)	3(4)	25(35)
ACAT	47	21	68	22(32)	3(4)	25(36)
Bonferroni	39	25	64	21(29)	4(3)	25(32)
LS-2015-5k: totally 1080218 blocks(noafr)						
oTFisher_cct	78	25	103	37(54)	7(7)	44(61)
GATES	49	13	62	20(33)	4(4)	24(37)
ACAT	50	14	64	21(34)	4(4)	25(38)
Bonferroni	52	23	75	25(37)	5(5)	30(42)
FA-2015-5k: totally 979645 blocks(noafr)						
oTFisher_cct	14	3	17	3(13)	1(2)	4(15)
GATES	15	1	16	3(14)	0(0)	3(14)
ACAT	15	1	16	3(14)	0(0)	3(14)
Bonferroni	15	1	16	3(14)	0(0)	3(14)
BMD-2017-5k: totally 293310 blocks(noafr)						
oTFisher_cct	9	0	9	6(8)	0(0)	6(8)
GATES	7	0	7	5(7)	0(0)	5(7)
ACAT	8	0	8	5(7)	0(0)	5(7)
Bonferroni	8	0	8	5(7)	0(0)	5(7)
TBBMD-2017-5k: totally 3093340 blocks(all)						
oTFisher_cct	166	123	289	75(149)	23(28)	98(177)
GATES	0	0	0	0(0)	0(0)	0(0)
ACAT	0	0	0	0(0)	0(0)	0(0)
Bonferroni	0	0	0	0(0)	0(0)	0(0)
Fracture-2018-5k: totally 336188 blocks(noafr)						
oTFisher_cct	28	7	35	11(18)	2(3)	13(21)
GATES	23	2	25	9(15)	1(1)	10(16)
ACAT	23	2	25	9(15)	1(1)	10(16)
Bonferroni	22	2	24	9(15)	1(1)	10(16)
FALLS-2020-5k: totally 666387 blocks(noafr)						
oTFisher_cct	8	10	18	4(3)	4(4)	8(7)
GATES	0	0	0	0(0)	0(0)	0(0)
ACAT	0	0	0	0(0)	0(0)	0(0)
Bonferroni	0	0	0	0(0)	0(0)	0(0)

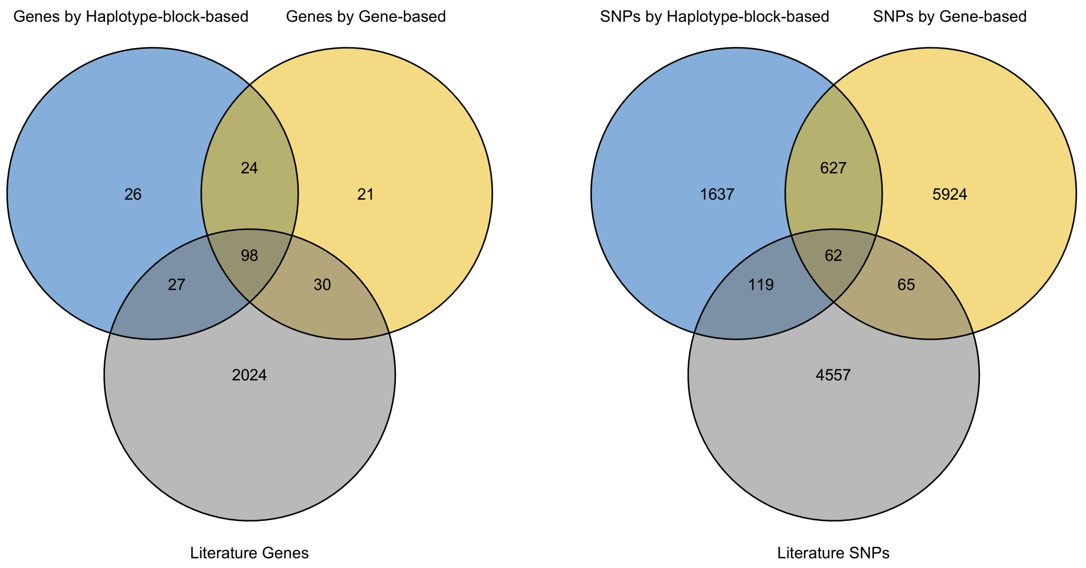

Figure S29: Venn diagrams show the overlap of the literature genes and SNPs found by haploblock analysis versus gene-based analysis. Left: Genes discovered based on the top hits by the oTFisher_cct test. Twenty-seven literature genes were found by haploblock analysis but not by gene-based analysis. Right: SNPs found by the oTFisher_r procedure. In total, 119 literature SNPs were detected only by haploblock analysis (63 of them are outside gene regions). Results are pooled from studies GEFOS2012_FN, GEFOS2012_LS, GEFOS2015_FN, GEFOS2015_LS, GEFOS2015_FA, GEFOS2017_BMD, GEFOS2017_TBBMD, and GEFOS2018_Fracture.

Novel haploblocks mapped to strong enhancers:

The top-hit blocks from all eight BMD studies contain 286 novel blocks (no overlap with literature genes or SNPs, 255 of them are outside of genes). The detailed block information is given in the supplementary file novel_blocks.xlsx.

We conducted epigenetic annotation for the novel blocks by Haploreg v3 (Ward and Kellis, 2016). Of 3061 SNPs covered by the 286 novel blocks, 240 SNPs are intronic, 3 SNPs are at 3'UTR, and 0 SNPs are at 5' UTR.

In total, 58 of these novel haploblocks can be mapped (by their SNPs) to strong enhancers of literature genes (results summarized in supplementary file novel_blocks_enhancers.xlsx).

In the following, we briefly discuss the SNPs in the novel blocks mapped to strong enhancers and/or promotors.

SNP rs1320596 in the GEFOS2012_FN top block and GEFOS2012_LS top block chr1:22688484 – 22690765 locates at the enhancer of cell K562 (leukemia) and is also close to a literature gene *ZBTB40* with a distance of 88539.

SNP rs76376051 in the GEFOS2015_LS top block chr1:22687982 – 22690364 locates at the enhancer of K562(leukemia) and is close to a literature gene *ZBTB40*.

SNP rs1021880 in the GEFOS2017_TBBMD top block chr1:110424529-110427190 located at the enhancer of cell K562 (leukemia) and is related to a literature gene *CSF1* with a distance of 28702.

A potential novel SNP rs17025223 in the GEFOS2017_TBBMD top block chr1:110490037-110493945 locates at the enhancer of cell HepG2 (hepatocellular carcinoma) and cell Huvec (umbilical vein endothelial cells) and is also close to a literature gene *CSF1* with a distance of 17985.

SNPs rs17045866, rs4671214, rs4671215, and rs7560205 in the GEFOS2012_LS top block chr2:54630902-54643692 are not only located at the enhancers of cell Huvec (umbilical vein endothelial cells) and cell NHEK (epidermal keratinocytes), but also located at the promoter of cell HepG2 (hepatocellular carcinoma). SNP rs17045866 also mapped to the cell Skeletal Muscle (SK.MUS) by Roadmap Epigenomics, which is associated with the delivery of bone morphogenetic protein-2 (Musgrave et al., 2002). Moreover, this SNP is close to a literature gene *SPTBN1* with a distance of 32812.

Three potential novel SNPs rs13393949, rs4671215, and rs7560205 in the GEFOS2012_LS top block chr2:54643778-54645650 locate at the enhancer of umbilical vein endothelial cells (Huvec), and they are related to the nearest gene *SPTBN1*, that is also a literature gene with the trait heel bone mineral density.

The top block chr2: 166552272 –166561432 of GEFOS2015_FN and GEFOS2015_LS contains 6 potential novel SNPs, and this block is close to a literature gene *CSRNP3*.

SNP rs1156916 and rs1156916 from top blocks chr3:41157575-41162974 of GEFOS2012_FN and GEFOS2012_LS is close to a literature gene *CTNNB1*, and it is also located at the enhancer of cell HepG2 (hepatocellular carcinoma).

SNP rs9878224 from top blocks chr3:41155615-41162974 of GEFOS2015_FN and GEFOS2015_LS is close to a literature gene *CTNNB1* with a distance of 81714. This SNP is also mapped to the enhancer of breast myoepithelial cells and skeletal muscle.

SNP rs34242730 in the GEFOS2017_TBBMD top block chr3: 156430628-156438321 locates at the enhancer of cell HMEC (mammary epithelial cells) from ENCODE, and it closes to a literature gene *TIPARP* with a distance of 12429. By Roadmap Epigenomics, this SNP also mapped to enhancers of BR.H35 (Breast vHMEC.Donor RM035), BR.MYO (Breast Myoepithelial Cells) and BM.MSC (Bone Marrow-Derived Mesenchymal Stem Cell Cultured Cells). SNP was also annotated to DNAse of cell HSMM (skeletal muscle myoblasts) by ENCODE.

SNPs rs10011284, rs10013836, rs12513126, rs13147471, rs1381949, rs17013531, rs17013562, rs17783296, rs17783320, rs2169611 and rs2169612 are in the GEFOS2012_FN top block chr4:88817225 – 88848016 and the GEFOS2012_LS top block chr4:88817225 – 88848016 locates at the enhancer of HepG2 (hepatocellular carcinoma) and they are also close to a literature gene *HSP90AB3P* with trait heel bone mineral density.

The top block chr5:88438444 – 88588302 from GEFOS2015_FN contains 25 potential novel SNPs at the enhancer of HSMM (skeletal muscle myoblasts), and this block is also close to a literature gene *MEF2C*.

SNP rs680620 from top block chr6:44562461-44564008 of GEFOS2017_TBBMD is mapped to the enhancers of skeletal muscle myoblasts and H1 cell line by ENCODE, where skeletal muscle myoblasts may express osteopontin (Uaesoontrachoon et al., 2008). This SNP is also close to a literature gene *CDC5L* with a distance of 147680.

The top block chr6:44548256 – 44554761 of GEFOS2017_TBBMD is mapped to the enhancer of HSMM (skeletal muscle myoblasts), and this block is also close to a literature gene *CDC5L*.

SNP rs1524073 in the GEFOS2017_TBBMD top block chr7: 38067611-38093233 locates at the enhancer of cell HSMM (skeletal muscle myoblasts) and is close to a literature gene *EPDR1* with a distance of 76068.

The top block chr7:38026156-38062167 of GEFOS2015_LS contains 10 potential novel SNPs rs117535055, rs118008598, rs1524050, rs17171245, rs185883438, rs67767155, rs6950561, rs73124894, rs78007943, and rs952369 and this block locates at the enhancer of cell HSMM (skeletal muscle myoblasts). The loci are close to a literature gene EPDR1 with the trait heel bone mineral density.

The top block chr7:96036979-96046387 of GEFOS2012_FN contains one potential novel SNP rs6971575, and the locus is at the enhancer of cell Huvec (umbilical vein endothelial cells). This block is also close to a literature gene *FLJ42280*.

The top block chr7:38035012-38050032 of GEFOS2017_TBBMD contains 8 potential novel SNPs rs12534806, rs1524050, rs1574004, rs67534599, rs6950561, rs73124894, rs78007943 and rs952369 and and this block locates at the enhancer of cell HSMM (skeletal muscle myoblasts). The locus is close to a literature gene *EPDR1* with the trait heel bone mineral density.

SNPs rs6971575 and rs6971215 from top block chr7:96036979-96041231 of GEFOS2017_TBBMD locate at the enhancers of cell Huvec (umbilical vein endothelial cells). The first SNP is also mapped to the enhancer of cell skeletal muscle (SK.MUS) by Roadmap. Furthermore, these two SNPs are close to a literature gene *FLJ42280*.

SNP rs73708344 from GEFOS2017_TBBMD top block chr7:96162479-96180537 locates at the enhancer of cell NHEK (epidermal keratinocytes) and is close to a literature gene *FLJ42280*.

SNP rs35110698 from GEFOS2015_FN top block chr7:28012003-28018998 locates at the enhancer of cell NHLF (lung fibroblasts) and is close to a literature gene *KLHDC5* with a distance of 63024.

SNPs rs3134086 and rs17684050 in the GEFOS2012_LS top block chr8:119831320-119856209 locate at the enhancer of cell HMEC (mammary epithelial cells) and are close to a literature gene *TNFRSF11B* with a distance of 79586. These SNPs also mapped to the enhancers of cell BR.H35 (Breast vHMEC.Donor RM035) and cell BM.MSC (Bone Marrow-Derived Mesenchymal Stem Cell Cultured Cells), which are associated with bone mineral density (van der Klift et al., 2003).

The top block chr8:119831320-119856209 of GEFOS2012_LS contains two potential SNPs, rs17684050 and rs3134086, and locates at the enhancers of HMEC (mammary epithelial cells) and NHEK (epidermal keratinocytes). This block is also mapped to a literature gene *TNFRSF11B* with trait bone mineral density.

SNP from chr10:79436344-79466701 of GEFOS2015_LS locates at the enhancer of cell GM12878 (B-lymphocyte, lymphoblastoid) and is also close to a literature gene *KCNMA1* with a distance of45942.

The top block chr11:121907885 – 21916663 contains 18 potential novel SNPs, and this block locates at the enhancer of cell NHLF (lung fibroblasts) and Huvec (umbilical vein endothelial cells). Moreover, a literature gene *MIR100HG* is close to the block.

The top block chr11:15829992-15838168 from GEFOS2017_TBBMD contains 5 potential novel SNPs rs11023730, rs1837606, rs2615044, rs2615045, and rs35840970. This block locates at the enhancers of cell HMEC (mammary epithelial cells), HSMM (skeletal muscle myoblasts), NHEK (epidermal keratinocytes), and NHLF (lung fibroblasts). Furthermore, a literature gene *SOX6* is close to the block.

SNP rs12281263 for top block chr11:15881098-15886388 of GEFOS2017_TBBMD locates at the enhancers of cell HMEC (mammary epithelial cells), and NHEK (epidermal keratinocytes). Furthermore, a literature gene *SOX6* is close to SNP.

The top block chr12:28012003-28018998 of GEFOS2015_FN contains three potential novel SNPs rs10743612, rs11049207, and rs258410, which are mapped to the enhancer of GM12878 (B-lymphocyte, lymphoblastoid). This block is also close to a literature gene *KLHDC5* related to femoral neck bone mineral density.

The top block chr12:90338518-90349999 of GEFOS2017_TBBMD contains four potential novel SNPs rs17836918, rs55973975, rs56162932, and rs7294671, which are mapped to the enhancer of GM12878 (B-lymphocyte, lymphoblastoid) and NHEK (epidermal keratinocytes). This block is also close to a literature gene *LOC338758*.

SNPs rs61926987 from top block chr12:90409494-90412549 of GEFOS2017_TBBMD locates at the enhancer of cell HSMM (skeletal muscle myoblasts). This block is also related to the closest gene *LOC338758*, a literature BMD gene.

SNPs rs61924662 from top block chr12:90485617-90486592 of GEFOS2017_TBBMD locates at enhancers of cell HSMM (skeletal muscle myoblasts) and cell NHEK (epidermal keratinocytes). This block is also related to the closest gene *LOC338758*, a literature BMD gene.

SNP rs17536071 in the gefos2012 top block chr13:43058031-43066117 locates at the enhancer of umbilical vein endothelial cells by ENCODE. Also, it is located at the enhancer of Bone Marrow-Derived Mesenchymal Stem Cell Cultured Cells by Roadmap. This SNP is related to a literature gene *TNFSF11*.

The top block chr13:42983701-43052880 from GEFOS2015_LS locates at the enhancer of cell GM12878 (B-lymphocyte, lymphoblastoid) and is also close to a literature gene *AKAP11*.

SNPs from top block chr13:43058974-43065232 from GEFOS2015_LS locates at the enhancer of cell Huvec (umbilical vein endothelial cells) and HMEC (mammary epithelial cells) and are also associated with a literature gene *TNFSF11*.

The top block chr13:42952499-42986645 from GEFOS2017_TBBMD contains eight potential novel SNPs, and this block is located at the enhancer of cell GM12878 (B-lymphocyte, lymphoblastoid). This block is also close to a literature gene *AKAP11*.

SNP rs9315922 from top block chr13:43070780 – 43099648 of GEFOS2012_LS and top block chr13:43066917 – 43085060 of GEFOS2017_TBBMD locates at the enhancer of cell GM12878 (B-lymphocyte, lymphoblastoid) and is also close to a literature gene *TNFSF11*.

The top block chr16:73097845-73101325 from GEFOS2017_TBBMD contains nine potential novel SNPs, and this block is located at the enhancer of cell NHLF (lung fibroblastsand) and Huvec (umbilical vein endothelial cells). This block is also close to a literature gene *ZFHX3*.

SNP rs1107747 in the top block chr17:41772813-41777286 of GEFOS2012_FN and GEFOS2012_LS is at the enhancer of cell HSMM (skeletal muscle myoblasts) and is also close to a literature gene *MEOX1*.

SNP rs16940132 from top block chr17:41786076-41799590 of GEFOS2018_Fracture, chr17:41797692-41798098 of GEFOS2012_FN and GEFOS2012_LS is located at the enhancer of cell HMEC (mammary epithelial cells), and this SNP is related to a literature gene *SOST*.

SNP rs2865162 in the top block chr20:39110179-39117995 of GEFOS2017_TBBMD locates at the enhancer of cell HMEC (mammary epithelial cells) and is also close to a literature gene *MAFB* with a distance of 197724.

The top block chr21:40319387-40320382 of GEFOS2018_Fracture contains two potential novel SNPs rs11702281 and rs6517491. This block locates at the enhancer of cell NHLF (lung fibroblasts) and is close to a literature heel BMD gene *ETS2*.

The top block chr21:36974873-37002836 of GEFOS2017_TBBMD contains seven potential novel SNPs located at the enhancer of cell HMEC (mammary epithelial cell), and the block is also related to a literature heel BMD gene *MIR802*.

3.5 Screening SNPs

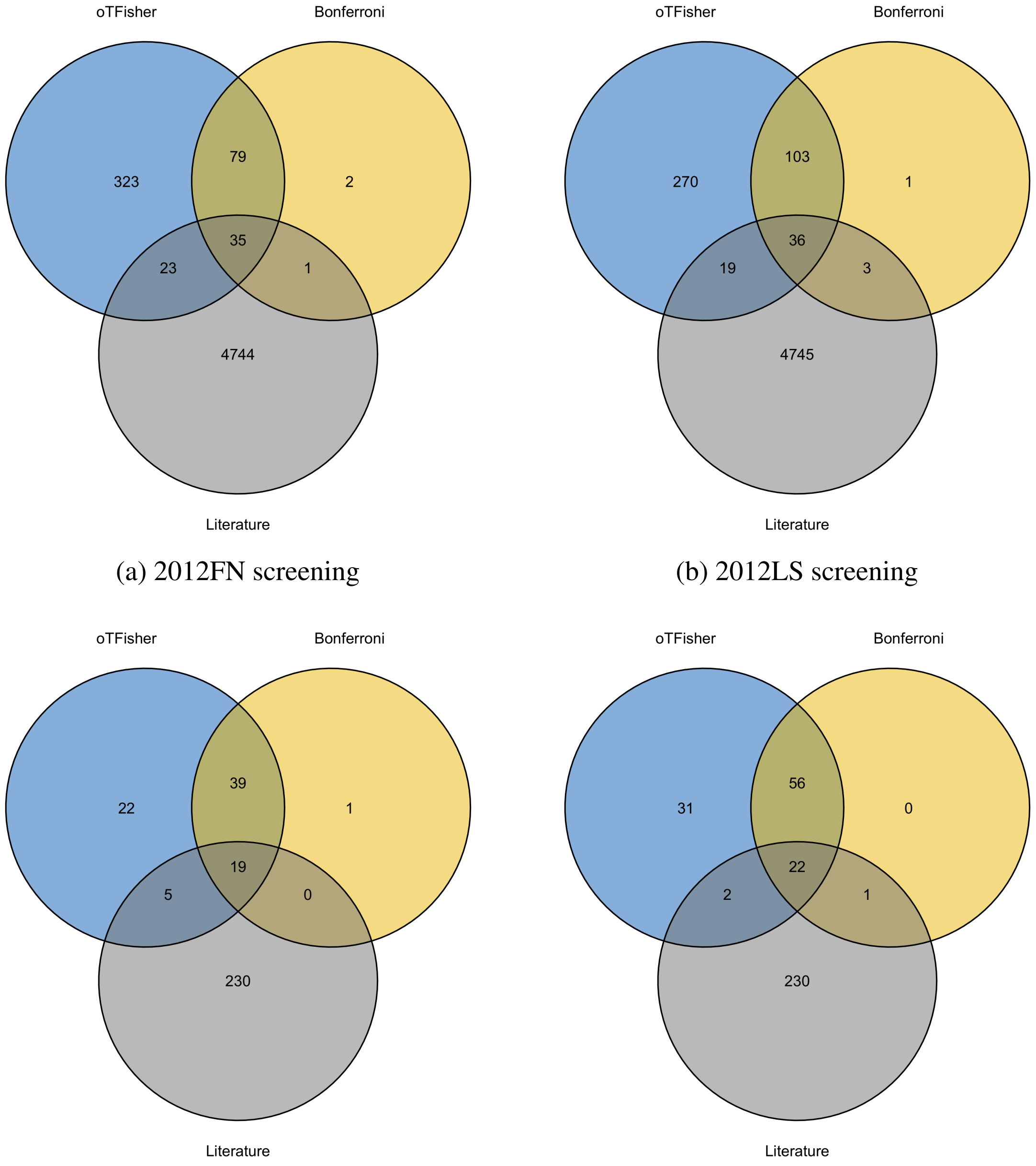

(a) 2012FN screening

(b) 2012LS screening

(c) 2012FN validation

(d) 2012LS validation

Figure S30: The GEFOS2012 data: Overlap of the screened SNPs in gene-based analysis (row 1) and the validated SNPs (row 2) based on the oTFisher_r, Bonferroni procedure, and literature. SNP-screening by GEFOS2012_FN (left column) and GEFOS2012_LS (right column) data; validation by the UKBB data. SNPs reported from the original GEFOS study were not included since many of them are outside of genes, and not comparable to the gene-based analysis result.

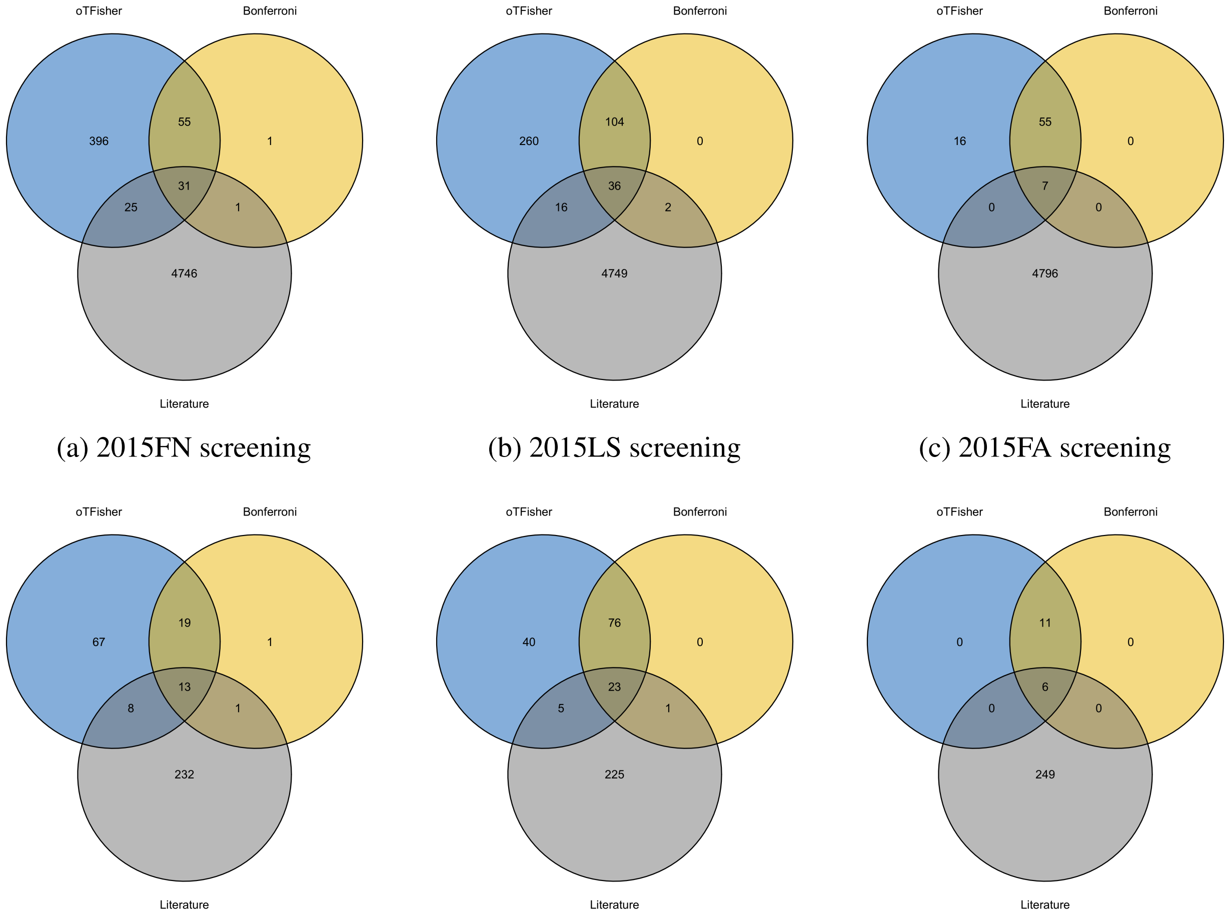

(a) 2015FN screening (b) 2015LS screening (c) 2015FA screening

(d) 2015FN validation (e) 2015LS validation (f) 2015FA validation

Figure S31: The GEFOS2015 data: Overlap of the screened SNPs in gene-based analysis (row 1) and the validated SNPs (row 2) based on the oTFisher_r, Bonferroni procedure, and literature. SNP-screening by GEFOS2015_FN (left column), GEFOS2015_LS (middle), and GEFOS2015_FA (right); validation by the UKBB data. SNPs reported from the original GEFOS study were not included since many of them are outside of genes, and not comparable to the gene-based analysis result.

REFERENCES

Ahmad, A., Strohbuecker, S., Tufarelli, C., and Sottile, V. (2017). Expression of a sox1 overlapping transcript in neural differentiation and cancer models. *Cellular and Molecular Life Sciences* 74, 4245–4258

Basit, S., Albalawi, A. M., Alharby, E., and Khoshhal, K. I. (2017). Exome sequencing identified rare variants in genes hspg2 and atp2b4 in a family segregating developmental dysplasia of the hip. *BMC Medical Genetics* 18, 1–10

Bulik-Sullivan, B. K., Loh, P.-R., Finucane, H. K., Ripke, S., Yang, J., Patterson, N., et al. (2015). Ld score regression distinguishes confounding from polygenicity in genome-wide association studies. *Nature genetics* 47, 291

Chang, C. C., Chow, C. C., Tellier, L. C., Vattikuti, S., Purcell, S. M., and Lee, J. J. (2015). Second-generation plink: rising to the challenge of larger and richer datasets. *Gigascience* 4, s13742–015

Deng, J.-E., Sham, P. C., and Li, M.-X. (2016). Snptracker: a swift tool for comprehensive tracking and unifying dbsnp rs ids and genomic coordinates of massive sequence variants. *G3: Genes, Genomes, Genetics* 6, 205–207

Estrada, K., Styrkarsdottir, U., Evangelou, E., Hsu, Y.-H., Duncan, E. L., Ntzani, E. E., et al. (2012). Genome-wide meta-analysis identifies 56 bone mineral density loci and reveals 14 loci associated with risk of fracture. *Nature genetics* 44, 491–501

Gemoll, T., Epping, F., Heinrich, L., Fritzsche, B., Roblick, U. J., Szymczak, S., et al. (2015). Increased cathepsin d protein expression is a biomarker for osteosarcomas, pulmonary metastases and other bone malignancies. *Oncotarget* 6, 16517

Gros-Louis, F., Larivière, R., Gowing, G., Laurent, S., Camu, W., Bouchard, J.-P., et al. (2004). A frameshift deletion in peripherin gene associated with amyotrophic lateral sclerosis. *Journal of Biological Chemistry* 279, 45951–45956

Higham, N. J. (2002). Computing the nearest correlation matrix—a problem from finance. *IMA journal of Numerical Analysis* 22, 329–343

Kim, S. K. (2018). Identification of 613 new loci associated with heel bone mineral density and a polygenic risk score for bone mineral density, osteoporosis and fracture. *PloS one* 13, e0200785

Lee, J. J., McGue, M., Iacono, W. G., and Chow, C. C. (2018). The accuracy of ld score regression as an estimator of confounding and genetic correlations in genome-wide association studies. *Genetic epidemiology* 42, 783–795

Li, P.-z., Yan, G.-y., Han, L., Pang, J., Zhong, B.-s., Zhang, G.-m., et al. (2017). Overexpression of stra8, boule, and dazl genes promotes goat bone marrow-derived mesenchymal stem cells in vitro transdifferentiation toward putative male germ cells. *Reproductive Sciences* 24, 300–312

Lin, D.-Y. and Zeng, D. (2010). On the relative efficiency of using summary statistics versus individual-level data in meta-analysis. *Biometrika* 97, 321–332

Medina-Gomez, C., Kemp, J. P., Dimou, N. L., Kreiner, E., Chesi, A., Zemel, B. S., et al. (2017). Bivariate genome-wide association meta-analysis of pediatric musculoskeletal traits reveals pleiotropic effects at the srebf1/tom1l2 locus. *Nature communications* 8, 121

Medina-Gomez, C., Kemp, J. P., Trajanoska, K., Luan, J., Chesi, A., Ahluwalia, T. S., et al. (2018). Life-course genome-wide association study meta-analysis of total body bmd and assessment of age-specific effects. *The American Journal of Human Genetics* 102, 88–102

Musgrave, D., Pruchnic, R., Bosch, P., Ziran, B., Whalen, J., and Huard, J. (2002). Human skeletal muscle cells in ex vivo gene therapy to deliver bone morphogenetic protein-2. *The Journal of Bone and Joint Surgery. British volume* 84, 120–127

Patel, J. K. and Read, C. B. (1996). *Handbook of the normal distribution* (New York: Marcel Dekker, INC.), 2nd edn.

Tachmazidou, I., Hatzikotoulas, K., Southam, L., Esparza-Gordillo, J., Haberland, V., Zheng, J., et al. (2019). Identification of new therapeutic targets for osteoarthritis through genome-wide analyses of uk biobank data. *Nature genetics* 51, 230–236

Trajanoska, K., Morris, J. A., Oei, L., Zheng, H.-F., Evans, D. M., Kiel, D. P., et al. (2018). Assessment of the genetic and clinical determinants of fracture risk: genome wide association and mendelian randomisation study. *bmj* 362, k3225

Trajanoska, K., Seppala, L. J., Medina-Gomez, C., Hsu, Y.-H., Zhou, S., van Schoor, N. M., et al. (2020). Genetic basis of falling risk susceptibility in the uk biobank study. *Communications biology* 3, 1–10

Uaesoontrachoon, K., Yoo, H.-J., Tudor, E. M., Pike, R. N., Mackie, E. J., and Pagel, C. N. (2008). Osteopontin and skeletal muscle myoblasts: association with muscle regeneration and regulation of myoblast function in vitro. *The international journal of biochemistry & cell biology* 40, 2303–2314

van der Klift, M., De Laet, C. E., Coebergh, J. W. W., Hofman, A., and Pols, H. A. (2003). Bone mineral density and the risk of breast cancer: the rotterdam study. *Bone* 32, 211–216

Ward, L. D. and Kellis, M. (2016). Haploreg v4: systematic mining of putative causal variants, cell types, regulators and target genes for human complex traits and disease. *Nucleic acids research* 44, D877–D881

Yu, K., Li, Q., Bergen, A. W., Pfeiffer, R. M., Rosenberg, P. S., Caporaso, N., et al. (2009). Pathway analysis by adaptive combination of P-values. *Genetic Epidemiology* 33, 700–709

Zheng, H.-F., Forgetta, V., Hsu, Y.-H., Estrada, K., Rosello-Diez, A., Leo, P. J., et al. (2015). Whole-genome sequencing identifies en1 as a determinant of bone density and fracture. *Nature* 526, 112–117
